# PDB2CD: a web-based application for the generation of circular dichroism spectra from protein atomic coordinates

**DOI:** 10.1093/bioinformatics/btw554

**Published:** 2016-09-20

**Authors:** Lazaros Mavridis, Robert W Janes

**Affiliations:** School of Biological and Chemical Sciences, Queen Mary University of London, London, UK

## Abstract

**Motivation:**

Circular dichroism (CD) spectroscopy is extensively utilized for determining the percentages of secondary structure content present in proteins. However, although a large contributor, secondary structure is not the only factor that influences the shape and magnitude of the CD spectrum produced. Other structural features can make contributions so an entire protein structural conformation can give rise to a CD spectrum. There is a need for an application capable of generating protein CD spectra from atomic coordinates. However, no empirically derived method to do this currently exists.

**Results:**

PDB2CD has been created as an empirical-based approach to the generation of protein CD spectra from atomic coordinates. The method utilizes a combination of structural features within the conformation of a protein; not only its percentage secondary structure content, but also the juxtaposition of these structural components relative to one another, and the overall structure similarity of the query protein to proteins in our dataset, the SP175 dataset, the ‘gold standard’ set obtained from the Protein Circular Dichroism Data Bank (PCDDB). A significant number of the CD spectra associated with the 71 proteins in this dataset have been produced with excellent accuracy using a leave-one-out cross-validation process. The method also creates spectra in good agreement with those of a test set of 14 proteins from the PCDDB. The PDB2CD package provides a web-based, user friendly approach to enable researchers to produce CD spectra from protein atomic coordinates.

**Availability and implementation:**

http://pdb2cd.cryst.bbk.ac.uk

**Supplementary information:**

Supplementary data are available at *Bioinformatics* online.

## 1 Introduction

Circular dichroism (CD) spectroscopy is a research technique used extensively worldwide. It is invaluable for providing structural information about proteins, particularly the secondary structure content. It is used for assessing whether a protein is correctly folded, is effective for monitoring structural changes induced through interactions either with other proteins or with ligands, and for determining protein stability through the application of temperature or pH changes. With the development and utilization of the technique, it was evident that a protein’s secondary structure content was the major contributor to its CD spectrum and many empirical methods were, and are still being created, to obtain this information from the spectrum (e.g. Brahms and Brahms, 1980; Chen and Yang, 1971; Hennessey and Johnson, 1981; Provencher and Glöckner, 1981; Sreerama *et al.*, 2000; Whitmore and Wallace, 2008; Wiedemann *et al.*, 2013). However, many of the papers reporting these methods added a cautionary warning that secondary structure content was not the only feature influencing the data, and that other factors needed to be considered. Newer methods have been developed to refine the secondary structure information obtained from spectra, for example the kinds of beta sheet arrangement present—parallel or anti-parallel (Micsonai *et al.*, 2015). It is clear from studying the spectra in the Protein Circular Dichroism Data Bank (PCDDB) (Whitmore *et al.*, 2011) that features from the whole protein structure contribute to the CD spectrum and this is even more evident when considering synchrotron radiation circular dichroism (SRCD) spectra which have an extended low-wavelength range expanding beyond that of conventional lab-based machines into the vacuum ultra-violet region.

The Protein Data Bank (PDB) (Berman *et al.*, 2000) is a resource that currently contains atomic coordinates of more than 100,000 protein structures. In comparison, the relatively new PCDDB currently contains just over 500 CD and SRCD spectra. Researchers might wish to compare the CD spectra obtained from proteins they are using in their research with those of proteins whose structures are found within the PDB, but whose spectra are not available in the PCDDB. In these cases other ways must be found to make such a comparison possible. An *ab initio* approach was developed, DichroCalc (Bulheller and Hirst, 2009) however whilst there are instances of reasonable agreements for the magnitudes of the CD spectra generated, especially for the peaks around 195 and 208 nm, it is clear that the important overall shapes are not accurate. This paper describes an alternative empirical method, PDB2CD, to enable the generation of CD (or SRCD) spectra from protein three-dimensional atomic coordinates as presented in a PDB file format. The input file can be either from a crystal structure, NMR structure (where the first model structure listed—usually the lowest energy one—will be used), molecular dynamics study, or a model protein structure.

Comparing protein atomic structures is often important but this may not be possible when one of those structures has not been determined. However, many laboratories have the capacity to collect CD spectra, thereby enabling some structural information to be gained about a protein. Inevitably the situation will arise where the structure of one protein exists while for the comparison protein only its CD spectrum is available. By generating CD spectra from protein atomic coordinates, PDB2CD facilitates structure comparisons. Many uses exist for such comparisons: confirmation of proposed or actual homology between two proteins, establishing that an expressed mutant protein has folded correctly and in a similar way to its wild-type protein, observing if structural differences arise upon the binding of different ligands, monitoring the effects of different environmental factors on conformation, and determining if structures calculated by molecular dynamics have CD spectra similar to experimental spectra. With the added information content that is offered by SRCD spectroscopy it may be possible in the future to determine if there are differences between the spectrum of a protein complex and the summed spectra of the individual components.

PDB2CD was developed using the atomic structures associated with the SRCD SP175 ‘gold standard’ spectral dataset comprising 71 entries found in the PCDDB (Lees *et al.*, 2006). PDB2CD employs a combination of three complementary structure-based approaches at different structural levels of detail to create a spectrum from a given protein structure. The first is based on the percentages of secondary structure present as alpha helix content, beta strand content and ‘other’ content (ABO), derived from Dictionary of Secondary Structure of Proteins (DSSP) values (Kabsch and Sander, 1983) calculated from the PDB files. The second compares the local near-space angular topologies of secondary structure components one with another defined by helix axis and strand vectors; notably alpha to alpha, beta to beta and alpha to beta (TOP). The last approach compares the overall structural similarities of the proteins themselves, providing a longer range global approach and a structural similarity score (ZSC) in each case. Utilizing these three approaches, weightings were produced for each of the methods developed to optimize the results of a leave-one-out approach for generating the CD spectra of the members of the SP175 dataset from the remaining members. This optimization minimized the overall differences between the experimental spectra and those generated. The combination of these three approaches was then used to produce spectra from a test set of proteins not used to create the method, as an independent validation of the procedure.

## 2 Methods

**Fig. 1.**
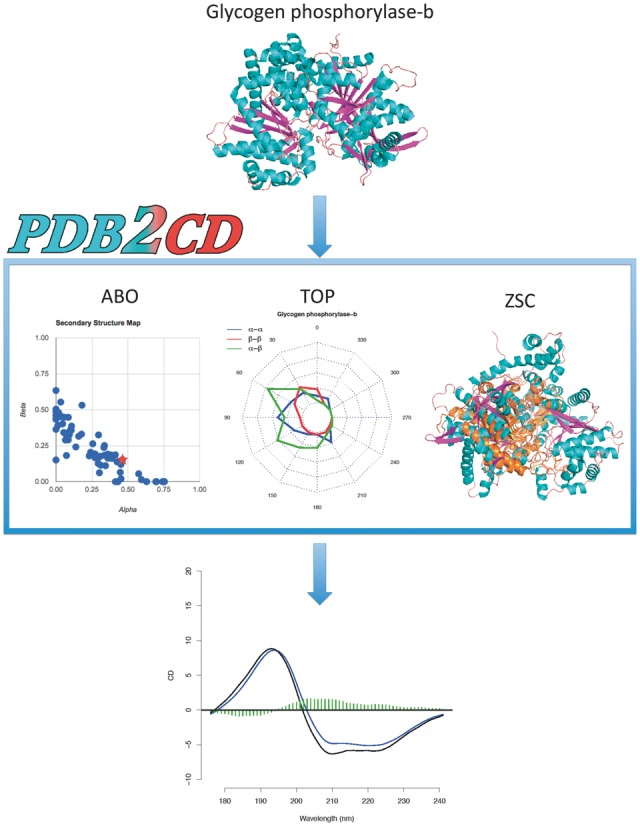
PDB2CD workflow based on the input structure of the query glycogen phosphorylase-b protein (PDB code 1gpb). This figure illustrates the results from the leave-one-out cross-validation test for this protein

The PDB2CD algorithm uses three different levels of structure-based information to generate a CD spectrum. These are the secondary structure content (ABO), the localized topological features of these secondary structure components (TOP), and the overall structural similarity between the query structure and the proteins within the basis dataset used (ZSC). The results from all three levels define proteins in the dataset with similar characteristics to the query protein. Finally the algorithm uses a refinement method to remove proteins from those selected whose CD spectra could be considered as outliers relative to the rest of the spectra, thereby leaving a set of proteins with CD spectra that can be used to create the resulting CD spectrum. Figure 1 shows the basic overview of the processes used in PDB2CD for generating a CD spectrum from a query protein structure. All protein structure images in this and subsequent figures are from the Rutgers Protein Data Bank website.

### 2.1 Dataset

The SP175 dataset (Lees *et al.*, 2006) was selected as the training set for the development as well as the initial validation of the method. The dataset consists of 71 protein spectra with solved crystallographic structures that cover secondary structural space (Fig. 1), plus a range of folds and different spectral shapes. This dataset provided an excellent challenge for the cross-validation as it only had a minimal number of structurally similar proteins: only 24 out of the 71 share a CATH classification (Cuff *et al.*, 2009) at the topology level.

### 2.2 Protein descriptors

The way that the structural neighbors for a given query protein are selected is based on the three different descriptors.

• **ABO**

For the ABO method the secondary structure content as defined by DSSP was calculated for each protein (Kabsch and Sander, 1983). The percentage of alpha helix (H in the DSSP notation) was used for the ‘A’ values, the percentage beta strand (E in DSSP) was used for the ‘B’ term, and all remaining contents were combined to become the percentage of other (the ‘O’ term).

• **TOP**

The TOP method utilizes vectors defined for each of the alpha and beta secondary structure components comprised of three or more consecutive residues found in proteins (Krissinel and Henrick, 2004). The angles between these vectors for three groups, alpha-to-alpha, beta-to-beta and alpha-to-beta interactions were calculated. These angle data were then used to derive a descriptor defining the overall topology around each of the secondary structure features for each protein giving their juxtaposition one with another. Data were binned into 12 equal 30° segments for each group, creating 36 bins in total, as shown in Figure 1. Only vectors less than or equal to 13 Å apart along their entire lengths were used to determine these angular profiles for each protein.

• **ZSC**

The ZSC is a measure of how similar the fold of two proteins is and is based on two components obtained from using the combinatorial extension (CE) protein–protein structural alignment method (Shindyalov and Bourne, 1998). The first is their Root Mean Square Deviation (RMSD) score defined as:

(1) $$RMSD=\sqrt{\frac{1}{N}\sum_{i=1}^{N}\delta_{i}^{2}}$$

where δ is the distance between *N* pairs of equivalent Cα atoms. The second is the number of residues that are aligned in the process. These terms have been combined to obtain the ZSC calculated value between the query protein and all the proteins of the SP175 dataset.

### 2.3 Matching

In order to identify the closest-matching structural neighbors of each query protein, all three descriptors are utilized.

**• ABO**

For the ABO score (ABOq,i) the Euclidean distance between the query protein and the rest of the proteins in the basis set is defined as:

(2) $$ABO_{q,i}=\sqrt{{\left(\alpha_{q}-\alpha_{i}\right)}^{2}+{\left(\beta_{q}-\beta_{i}\right)}^{2}+{\left(o_{q}-o_{i}\right)}^{2}}$$

where *q* is the query protein and *i* the *i*th protein in the basis set, and α, β and ο are the percentages of alpha helix, beta strand and other components, respectively. The three proteins with the closest scores are selected that satisfy the rule in Equation 3:

(3) $$\alpha_{i}<\alpha_{q}<\alpha_{p}\;(or\;\beta_{i}<\beta_{q}<\beta_{p})$$

where α_i_ and α_p_ represent the values for two of the three top matches of the ABO scores (in the case of mainly helical proteins; otherwise the β values are used for the rule). Application of this rule ensures that the query protein data are encompassed by the basis set data to limit potential bias to the generated final resulting spectrum. In cases where no match to Equation 3 can be satisfied, then the top three matches to Equation 2 are returned.

**• TOP**

The TOP score (TOP_q,d_) is again calculated using the Euclidean distance between the query protein and the rest of the proteins in the basis set as:

(4) $$TOP_{q,d}=\sqrt{\;{\left(\sum_{i=1}^{36}q_{i}-d_{i}\right)}^{2}\;}$$

where *q* is the query protein, *d* is one of the proteins in the basis set, and *q_i_* and *d_i_* represent the TOP descriptors as identified above. The top three matches are retained for production of the final spectrum.

**• ZSC**

The ZSC calculation arises from implementation of CE in Java (Prlic *et al.*, 2012). This is the method used by the PDB for comparing structures. First, a structural alignment between the query and itself is recorded as a normalizing factor. All basis set proteins are then aligned against the query protein and their pair-wise scores are divided by the normalizing factor. For instances where the query and/or the basis set protein is comprised of more than one polypeptide chain, once the normalization step has been performed (each chain being compared against itself for the query protein), then pairwise scores are obtained for each chain of one protein against those of the other. The maximum overlap between structures can then be determined by summing the highest pairwise scores between each chain where no chains are used more than once in the calculations.

### 2.4 Refinement

The last step before generation of the spectrum is to identify and remove spectral matches which are significantly different from the majority of the retrieved spectra; these are potential outliers that might bias an overall result. This refinement process is performed by repeating the following steps until all remaining protein spectra fall within the applied constraints:

(1) Calculate a mean spectrum CD_µ_ as:

(5) $$CD_{µ}=\frac{1}{n}\left(\sum CD_{A_{1.3}}+\sum CD_{T_{1.3}}+\sum CD_{Z_{1.3}}\right)$$

where *n* is the number of spectra remaining, and CD_A(1.3)_, CD_T(1.3)_ and CD_Z(1.3)_ represent any of the top three CD spectra for the ABO, TOP and ZSC methods, respectively.

(2) Calculate the standard deviation σ, at each wavelength point l, between the mean and the remaining spectra.

(3) For each of the remaining spectra, determine the number Pi of wavelength positions where the following is true:

(6) $$P_{i}=\sum_{l=175}^{240}I(\left|CD_{i,l}\right|>\left|CD_{µ,l}\right|*2\sigma_{l})$$

(4) If max(P) for any remaining protein is more than one sixth of the total number of wavelengths, then that protein is identified as significantly different from the rest and is removed.

(5) This process is repeated if a protein is removed.

The remaining proteins are then considered to have spectra that can be used to create the final CD spectrum.

### 2.5 Final generation of the CD spectrum

The remaining proteins with the native scores from the three different approaches are used to produce the final CD spectrum as:

(7) $$CD_{F}=\frac{1}{n_{A}+n_{T}+n_{Z}}\left(\sum_{i=1}^{n_{A}}A_{i}CD_{i}+\sum_{i=1}^{n_{T}}T_{i}CD_{i}+\sum_{i=1}^{n_{Z}}Z_{i}CD_{i}\right)$$

where CD_F_ is the generated CD spectrum, *n*_A_, *n*_T_ and *n*_Z_ are the numbers of remaining proteins from the ABO, TOP and ZSC matches, respectively, after refinement. A_*i*_, T_*i*_ and Z_*i*_ are the ABO, TOP and ZSC scores between the query and the retained protein *i*, respectively, and CD_*i*_ is the CD spectrum from the *i*th protein of the basis set.

### 2.6 Cross-validation

In order to validate PDB2CD, a leave-one-out cross-validation was performed using the SP175 dataset, and comparison was made between the generated spectra and those calculated using DichroCalc (Bulheller and Hirst, 2009). Each dataset member was removed in turn and its structure was then used as the query with the remaining 70 proteins being used to create its spectrum from the spectra obtained from the structural matches. As DichroCalc did not need training, the 71 protein structures of the SP175 dataset were individually submitted to the server (accessed between 3rd and 5th June 2015). Included in the calculations were the backbone charge-transfer transitions, the aromatic (Phe, Tyr and Trp) side chain transitions and side chain transitions of Asn, Asp, Gln and Glu amino acids. The NRMSDs were calculated according to the following equation for each of the 71 proteins for both PDB2CD and DichroCalc to compare their results.

(8) $$NRMSD=\sum_{l=175}^{240}\sqrt{\frac{{\left(e_{l}-o_{l}\right)}^{2}}{M}}$$

where *e_l_* is the generated and *o_l_* the experimentally observed magnitude at wavelength *l*, respectively, and *M* is the square root of the sum of squares at the wavelength for the maximum observed difference between spectra in the basis set. This is similar to the NRMSD term used in the DichroWeb secondary structure analysis server (Whitmore and Wallace, 2008), but with a modified normalization term.

### 2.7 Optimization of parameters

For all optimization strategies, the measure of improvement (or not) was determined by obtaining the overall NRMSD values together with the numbers of ‘good’ and ‘poor’ spectra identified in each case. Here, NRMSD values less than or equal to 0.1 were defined as ‘good’, while those greater than or equal to 0.2 were defined as ‘poor’. For the ABO descriptor it was established that adding in 3_10_ helix as a component of ‘other’ gave better results than when it was a component of the A term. For the topology descriptor (TOP) the magnitude of gap between the vectors associated with the alpha helices, the beta strands and alpha and beta components was varied during method development across a 10–99 Å range in 1 Ångstrom steps. It was identified that 13 Å was the optimum for defining this characteristic (data not shown).

Three to ten closest matches for each of the three structure descriptors were tested for retention during optimization of the refinement strategies. The results clearly demonstrated that, for the significant majority of the SP175 validation dataset, any number greater than three retained for each descriptor raised the overall NRMSD value and introduced noise into the derived spectrum, shifting it away from the optimal answer. Three was therefore chosen as the retention value.

### 2.8 Test data

Although the PCDDB has just over 500 CD entries many of these are either thermal scans, membrane proteins (Abdul-Gader *et al.*, 2011), or (predominantly), entries for which there is no structure available in the PDB. Fourteen soluble proteins were identified in the PCDDB which satisfied the criteria of having well-determined CD spectra, as shown by their ValiDichro reports (Woollett *et al.*, 2013) and having associated PDB structures. These were used as a wholly independent further test set for the PDB2CD method.

## 3 Results

### 3.1 Final generation of the CD spectrum

The majority of CD spectra are generated using the data from seven or more proteins where seven is the most common number employed. However these might either be as seven unique proteins or as seven proteins containing both unique proteins and multiples of one or more proteins where more than one of the methods has picked the same protein to be used. This acts as one way by which the PDB2CD approach weights the member proteins being used in the set to generate a CD spectrum. It is clear that if two or more of the approaches pick the same protein then there must be a distinct similarity between the query protein and that data base entry which would likely mean that there is a similarity between their CD spectra.

### 3.2 Cross-validation

**Fig. 2.**
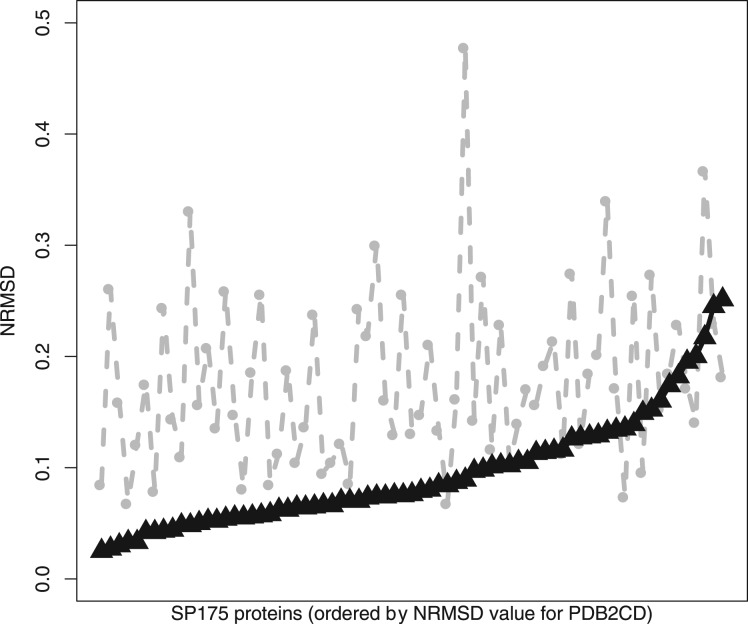
The NRMSDs (from smallest values to largest values) between the experimental CD spectra and PDB2CD-generated spectra (black triangles), and for comparison, with the DichroCalc-predicted spectra (grey dots) are plotted for all 71 proteins in the SP175 dataset

The spectra generated by PDB2CD for each protein were compared with DichroCalc-predicted spectra for the same 71 proteins, shown in Figure 2. They are ordered on the plot from left to right according to the smallest to largest NRMSD differences between the experimental spectra and those generated by the PDB2CD method. These results represent the best obtained following the optimization of the parameters employed in PDB2CD. Table 1 shows that using the individual structure methods, ABO, TOP and ZSC separately, produces results which are better than DichroCalc but are less good than the results obtained when using the collective approach. From Table 1, the combination of all three methods in PDB2CD performs better than DichroCalc in all categories: notably the overall average NRMSD for all cases is 0.09 for PDB2CD and 0.18 for DichroCalc. Applying the NRMSD threshold of 0.1 and below to define a ‘good’ prediction and a value of 0.2 and above to define a ‘poor’ prediction, PDB2CD has 44 good cases (62%) and only 4 (6%) poor ones. In contrast DichroCalc is good only in 10 cases (14%) and poor in 23 (32%).

**Table 1.** Comparison of goodness-of-fit (NRMSD) for the individual structure methods in PDB2CD, for the combination of these methods used in PDB2CD and for the DichroCalc method

	**SP175**					**14 Proteins**	
Category	*ABO*	*TOP*	*ZSC*	PDB2CD	DichroCalc	PDB2CD	DichroCalc
Overall NRMSD	0.11	0.12	0.14	**0.09**	0.18	**0.10**	0.17
Worst NRMSD	**0.25**	0.39	0.41	**0.25**	0.48	**0.18**	0.46
Best NRMSD	0.03	**0.02**	0.04	0.03	0.07	**0.04**	0.06
No. of ‘Good’ Cases	34	29	28	**44**	10	**7**	6
No. of ‘Poor’ Cases	**3**	7	15	4	23	**0**	3

Left: Cross-validation (SP175) and Right: Test (14 Protein) dataset. Results in bold are the methods that perform the best in the given category.

**Fig. 3.**
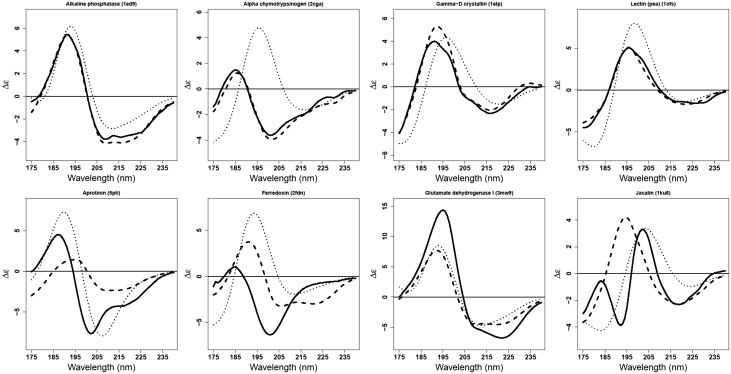
On the top row, results for the four best proteins: alkaline phosphatase (1ed9), α-chymotrypsinogen (2cga), γ-D crystallin (1elp) and lectin (pea) (1ofs) and on the bottom row, results for the four poorest proteins: aprotinin (5pti), ferrodoxin (2fdn), glutamate dehydrogenase I (3mw9) and jacalin (1ku8). In black is the measured CD spectrum; in dashed the spectrum produced by PDB2CD; in dotted the calculated spectrum from DichroCalc

Figure 3 gives examples of the four best and four poorest spectra from PDB2CD overlaid on the corresponding experimental and DichroCalc-predicted spectra. In the cases of alkaline phosphatase and γ-D crystallin, DichroCalc also performs well but not as good as PDB2CD. In contrast, where PDB2CD generates a very close match to the experimentally measured CD spectra of α-chymotrypsinogen and lectin (pea), DichroCalc fails to properly calculate these spectra. Four proteins aprotinin, ferrodoxin, glutamate dehydrogenase I and jacalin prove challenging cases for both PDB2CD and DichroCalc; both methodologies fail to produce an accurate result for these spectra although it is interesting to note that they produce comparable results for glutamate dehydrogenase I.

**Fig. 4:**
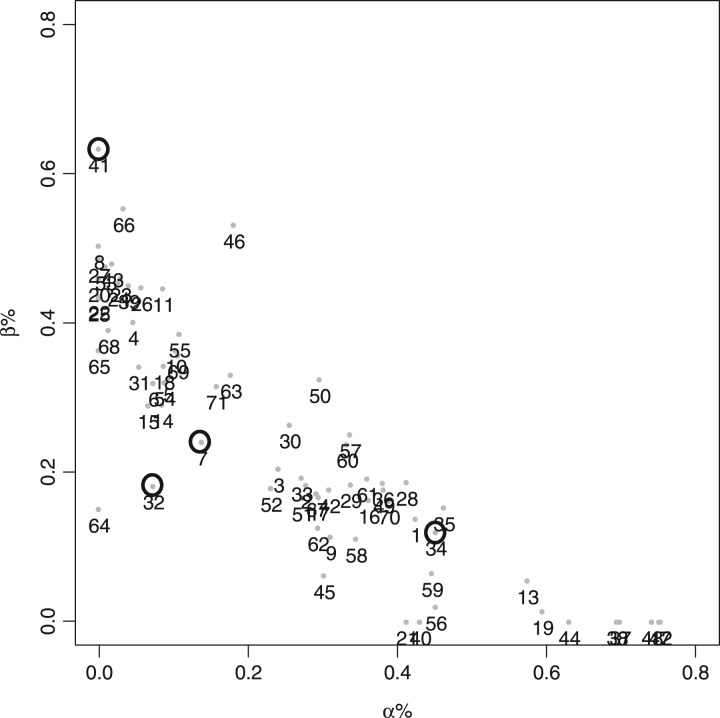
The normalized percentage alpha against beta secondary structure map of the 71 proteins of the SP175 as defined by the ABO term. The circled points indicate the positions for the four poor PDB2CD spectra generated in the leave-one-out cross-validation procedure. The numbers refer to the order of proteins in the SP175 dataset listed in the order of their PCDDB codes

Figure 4 plots the secondary structure, helix (α) versus strand (β) content, for the SP175 dataset with these four proteins identified (circled). These reside in sparsely populated areas of the plot which means that there are limited structural neighbors in these regions from which to calculate an appropriate spectrum. It is notable that glutamate dehydrogenase I (no. 34 in Fig. 4) with slightly more neighbors has a similar generated shape to that of the experimentally determined spectrum although the magnitudes are substantially different.

### 3.3 Test data

**Fig. 5:**
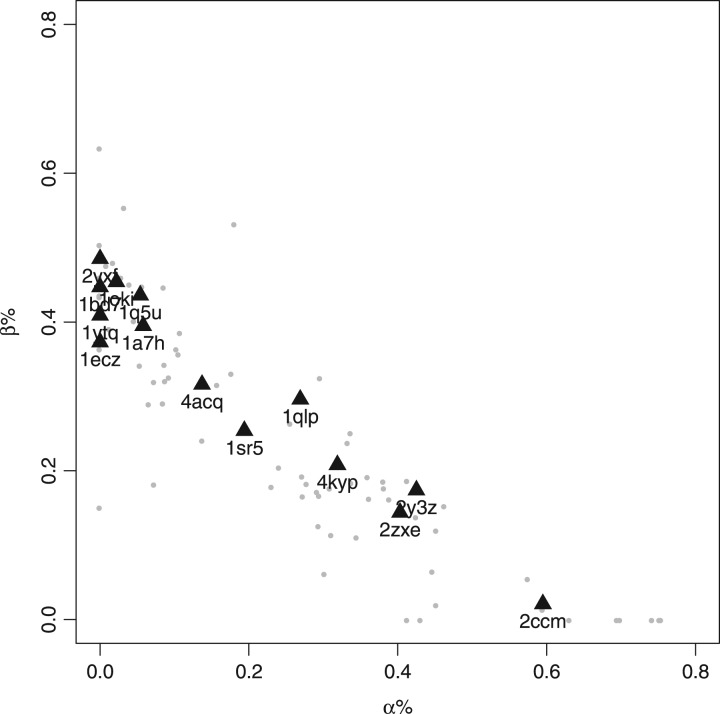
The normalized percentage alpha against beta secondary structure map of the 71 proteins of the SP175 dataset showing the additional 14 test proteins used (black triangles)

**Fig. 6:**
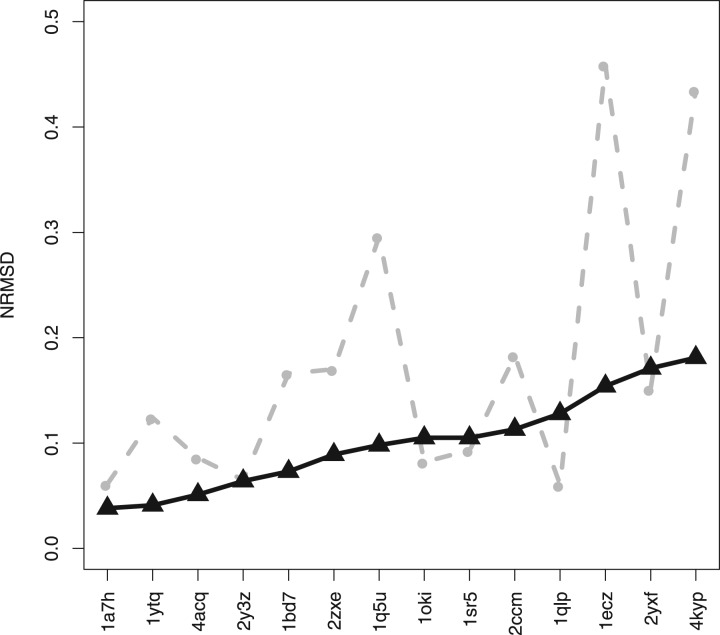
The NRMSDs (ordered from smallest values to largest values for the PDB2CD method) between the experimental CD spectra and PDB2CD-generated spectra (black triangles) and DichroCalc predicted spectra (grey dots) plotted for the set of 14 test proteins

PDB2CD was used to produce spectra for fourteen soluble proteins from the PCDDB employed as a further independent test set. These proteins are diverse and cover most of secondary structure space as can be seen in Figure 5. It is notable that a number of them have high beta strand with low alpha helix content, as proteins of this kind often have greater spectral shape diversity in comparison to those proteins with a high alpha helical content (Miles and Wallace, 2006). Similar to the cross-validation study, the 14 proteins were run using both methods and the results are shown in Figure 6 and summarized in Table 1.

**Fig. 7:**
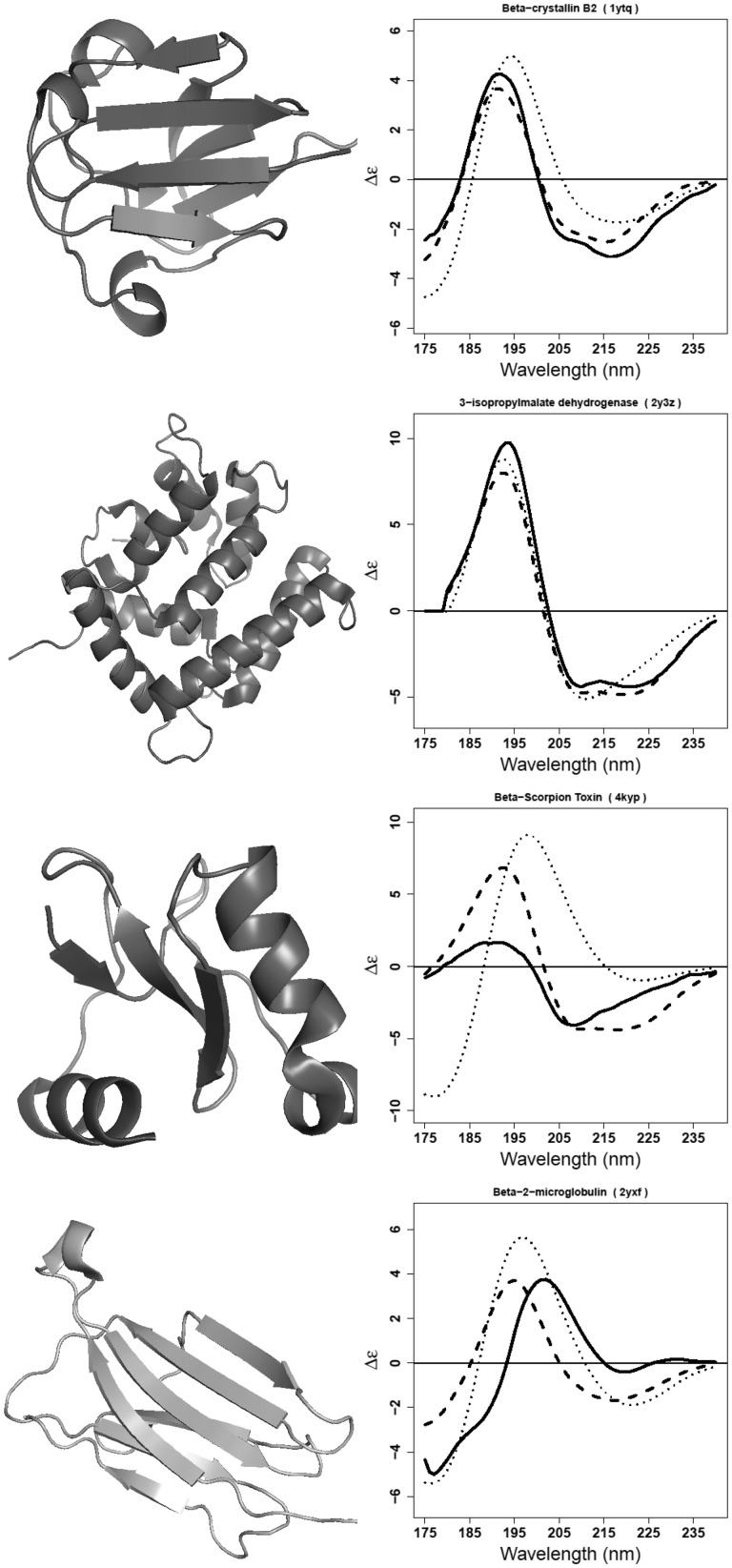
Spectra for β-crystallin B2 (1ytq), 3-isopropylmalate dehydrogenase (2y3z), β-scorpion toxin (4kyp) and β2-microglobulin (2yxf). In black, the experimental CD spectra; in dashed the generated spectra from PDB2CD; in dotted the calculated spectra from DichroCalc

Two representative good results and two poorer results from the PDB2CD generated spectra for these test proteins are shown in Figure 7. For β-crystallin B2 (1ytq) it can be seen that PDB2CD has reasonably produced both the peak magnitudes and shapes in comparison to the experimental spectrum. In contrast, DichroCalc poorly calculates both peak magnitudes and overall shapes. For 3-isopropylmalate dehydrogenase (2y3z) the NRMSD values as shown in Figure 6 are very comparable for this protein for the two methods (0.064 and 0.065 for PDB2CD and DichroCalc respectively). However, when looking at the resultant spectra presented by the two methods for 2y3z it is clear to see that the overall shape is significantly better for PDB2CD because it successfully produces the peak at around 222 nm whereas DichroCalc, has failed to do this. The other two selected proteins, are the β-scorpion toxin (4kyp) and β2-microglobulin (2yxf); for this pair of proteins PDB2CD does not perform as well. It is worth noting that the 4kyp protein is small in size, and contains a number of disulfide bonds which can contribute minor characteristics to CD spectra. Being small there are a limited number of secondary structure features within this protein and they are well-separated from each other, consequently for the TOP method there will be a limited number of interactions which in turn will limit the number of parameters available for use within the PDB2CD method. However, the calculated spectrum for DichroCalc is even further away from the experimental spectrum (Fig. **7**). β2-Microglobulin (2yxf) clearly represents a difficult case for both approaches; while PDB2CD produces better relative magnitudes comparable to the experimental spectrum and DichroCalc produces better relative peak positions; neither method accurately combines these features to give a close result.

The results from the leave-one-out cross-validation agree well with the results of the independent test set of 14 proteins taken from the PCDDB when comparing the data in Table 1.

### 3.4 Potential uses of PDB2CD

**Fig. 8:**
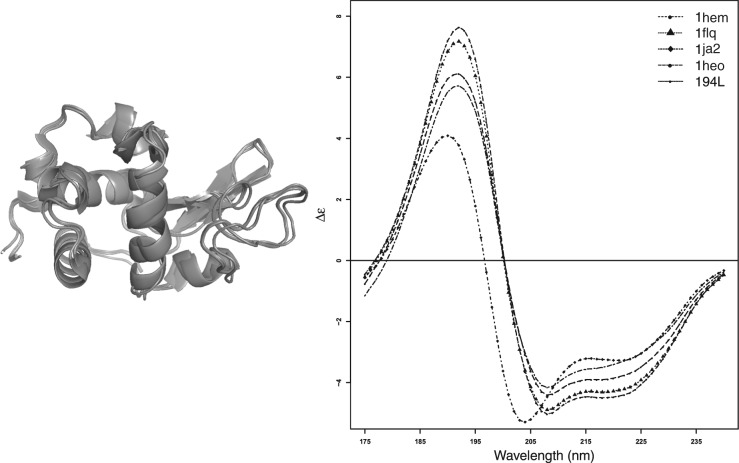
Lysozyme test data: A structural overlay of the five different crystal structures of lysozyme (1HEM, 1FLQ, 1JA2, 1HEO and 194l this one being in SP175 dataset) and their matching generated CD spectra obtained using PDB2CD, except for that for 194l which is the experimental spectrum from the PCDDB entry

Circular dichroism can provide spectra that can highlight subtle, small differences between protein conformations. To illustrate what PDB2CD might be able to show in this respect, CD spectra were generated for a limited group of related lysozyme structures where sequence mutations from the wild-type protein, given by PDB code 194l, or a powder diffraction study, given by 1ja2, gave rise to small differences in their secondary and tertiary structure contents. These results are shown in Figure 8. Although the experimentally determined spectra of these lysozyme structures (other than the 194l, whose spectrum is shown from the SP175 dataset) do not exist, nevertheless these generated spectra illustrate one of the potential uses for PDB2CD and shows subtle differences in structure do give rise to comparable differences in generated CD spectra (further illustrated in Supplementary data). PDB2CD therefore offers a way to gain information about structural differences between proteins where none would otherwise be possible and hence it should be a useful tool for generating CD spectra from protein atomic coordinates.

## 4 Discussion and conclusions

In general it can be seen from the results presented here that the PDB2CD methodology offers a significantly more accurate and improved approach to the current (*ab initio*) method, DichroCalc, for generating CD spectra from PDB-formatted protein structure files. However, some note should be made regarding cases where the method does not accurately match known experimental CD spectra, and the potential reasons why. The simplest reason is that some of these instances result from a lack of structures in the data base set with similar features to that of the query protein, which can restrict the accuracy of the comparisons used within the PDB2CD method. However there are other more fundamental possible reasons for poor matches: First, a crystal structure reflects the conformation of a protein in a constrained ‘solid state’, which may differ from its structure in solution. For many structures these two states may be very similar, if not the same, but for others, they differ considerably. Second, some proteins are very flexible, being able to adopt a multitude of different conformational states within the time frame of a CD measurement. The recorded CD spectrum will be the average of these states, and this average may well differ from that of a single crystal structure, which is a ‘snapshot’ view that may or may not represent an energy minimum conformation of that protein. Furthermore, in some crystals some areas may be so flexible that they are not observable in the electron density map, and hence do not appear in the coordinate file. They would thus not contribute to the calculated spectrum, although the measured spectrum will have contributions from all of the conformations that exist in a solution. Interestingly, where there are differences between the calculated CD spectrum and the measured spectrum, this may be a flag of interest as an indication of the conformational flexibility of the protein. Third, comparisons of PDB files of the same protein prepared under different crystallization conditions or in different crystal forms indicate that whilst many proteins retain similar structures in different environments, there are also many examples of proteins where crystal structures of the same protein are very different. This, too, could be an explanation for some of the ‘poor’ fits; hence the user might be advised to test the similarity of PDB2CD-calculated spectra from different PDB files of the same protein, as an indication of this variability.

These crystal-dependent effects might suggest that for some proteins NMR solution structures might better be used to generate CD spectra using PDB2CD, as this method measures the range of structures in the solution. There are also inherent issues here, however, as the solution structure is dynamic and as a result a highly flexible protein will have a greater degree of imprecision in the NMR results. An ensemble of structures is usually generated from NMR data, but for a flexible protein this could represent an even broader set of possible conformations. Typically the first model in an NMR file of an ensemble of structures is either the lowest energy structure or the energy minimized average structure of the ensemble. The CD spectrum generated from either protein model can differ considerably, but again could offer valuable insight from these differences. Of course, all of the above precautions are not exclusive to our PDB2CD methodology, but would also be an issue for DichroCalc or any other method which seeks to characterize dynamic structures in solution. Finally, PDB2CD uses three different structure-based levels of information to generate the far UV CD spectra of a protein from its PDB coordinates. The accuracy of the method is predicated on the fact that for the majority of proteins, the peptide chromophores are the only significant contributors to the spectrum produced. However, there can be some contributions to a CD spectrum in the wavelength range usually attributable to transitions from the peptide bonds (from ∼190 to 230 nm) which can arise from the side chains of specific residues, most notably aromatic residues (Manning and Woody, 1999; Ohmae *et al.*, 2015). Disulfide bonds can also contribute to the shape of a CD spectrum in wavelength regions towards the upper ends of this range, if they comprise a significant proportion of the residues in the protein (e.g. in small toxins). In addition to their own small spectral contributions, disulfide groups can also constrain structures into non-canonical phi, psi angles and less flexible conformations, which in themselves may contribute further unique features to a CD spectrum. Just what contributions specific aromatic residues and disulfide bonds would make to an individual CD spectrum are unclear as they are very much dependent upon the local environment and high resolution details of the protein conformation. Given the limited size of the SP175 dataset used to create PDB2CD there are insufficient numbers of protein examples to develop protocols to establish the contributions of side chains to the overall CD spectrum. In the future, as the number of high quality CD spectra in the PCDDB (Whitmore *et al.*, 2011) grows, they can be added to the PDB2CD reference dataset and should improve such calculations.

In conclusion, the PDB2CD method provides a novel empirical means of generating a CD spectrum based on a protein's atomic coordinate file. The PDB2CD approach has been cross-validated with one of the most complete and well known datasets of CD spectra with related crystal structures, SP175, and tested on an additional 14 proteins. It has been found to do consistently better than the existing *ab initio* DichroCalc method at replicating known protein spectra. The algorithm is stable in the quality of its predictions with an average NRMSD of less than 0.095, with a significant number of ‘good’ matching cases (62%) and with a very low percentage of ‘poor’ cases (∼6%). PDB2CD is publicly available (http://pdb2cd.cryst.bbk.ac.uk) without requirement for user login.

## Supplementary Material

Supplementary Data

## References

[btw554-B1] Abdul-Gader A. et al (2011) A reference data set for the analyses of membrane protein secondary structures and transmembrane residues using circular dichroism spectroscopy. Bioinformatics, 27, 1630–1636. 21505036 10.1093/bioinformatics/btr234

[btw554-B2] Berman H.M. et al (2000) The protein data bank. Nucleic Acids Res., 28, 235–242. 10592235 10.1093/nar/28.1.235 PMC102472

[btw554-B3] Brahms S., Brahms J. (1980) Determination of protein secondary structure in solution by vacuum ultraviolet circular dichroism. J. Mol. Biol., 138, 149–178. 7411608 10.1016/0022-2836(80)90282-x

[btw554-B4] Bulheller B.M., Hirst J.D. (2009) DichroCalc – circular and linear dichroism online. Bioinformatics, 25, 539–540. 19129206 10.1093/bioinformatics/btp016

[btw554-B5] Chen Y.H., Yang J.T. (1971) A new approach to the calculation of secondary structures of globular proteins by optical rotatory dispersion and circular dichroism. Biochem. Biophys. Res. Commun., 44, 1285–1291. 5168596 10.1016/s0006-291x(71)80225-5

[btw554-B6] Cuff A.L. et al (2009) The CATH classification revisited: architectures reviewed and new ways to characterize structural divergence in superfamilies. Nucleic Acids Res., 37, D310–D314. 18996897 10.1093/nar/gkn877 PMC2686597

[btw554-B7] Hennessey J.P. Jr, Johnson W.C. Jr (1981) Information content in the circular dichroism of proteins. Biochemistry, 20, 1085–1094. 7225319 10.1021/bi00508a007

[btw554-B8] Kabsch W., Sander C. (1983) Dictionary of protein secondary structure: pattern recognition of hydrogen-bonded and geometrical features. Biopolymers, 22, 2577–2637. 6667333 10.1002/bip.360221211

[btw554-B9] Krissinel E., Henrick K. (2004) Secondary structure matching (SSM), a new tool for fast protein structure alignment in three dimensions. Acta Crystallogr., D60, 2256–2268. 10.1107/S0907444904026460 15572779

[btw554-B10] Lees J.G. et al (2006) A reference database for circular dichroism spectroscopy covering fold and secondary structure space. Bioinformatics, 22, 1955–1962. 16787970 10.1093/bioinformatics/btl327

[btw554-B11] Manning M.C., Woody R.W. (1989) Theoretical study of the contribution of aromatic side chains to the circular dichroism of basic bovine pancreatic trypsin inhibitor. 28, 8609–8613. 10.1021/bi00447a051 2481497

[btw554-B12] Micsonai A. et al (2015) Accurate secondary structure prediction and fold recognition for circular dichroism spectroscopy. Proc. Natl. Acad. Sci. U. S. A., 112, E3095–E3103. 26038575 10.1073/pnas.1500851112 PMC4475991

[btw554-B13] Miles A.J., Wallace B.A. (2006) Synchrotron radiation circular dichroism spectroscopy of proteins and applications in structural and functional genomics. Chem. Soc. Revs., 35, 39–51. 16365641 10.1039/b316168b

[btw554-B14] Ohmae E. et al (2015) Vacuum-ultraviolet circular dichroism spectra of *Escherichia coli* dihydrofolate reductase and its mutants: contributions of phenylalanine and tyrosine side chains and exciton coupling of two tryptophan side chains. J. Phys. Chem. B, 119, 13002–13008. 26407224 10.1021/acs.jpcb.5b07480

[btw554-B15] Prlic A. et al (2012) BioJava: an open-source framework for bioinformatics in 2012. Bioinformatics, 28, 2693–2695. 22877863 10.1093/bioinformatics/bts494 PMC3467744

[btw554-B16] Provencher S.W., Glöckner J. (1981) Estimation of globular protein secondary structure from circular dichroism. Biochemistry, 20, 33–37. 7470476 10.1021/bi00504a006

[btw554-B17] Shindyalov I.N., Bourne P.E. (1998) Protein structure alignment by incremental combinatorial extension (CE) of the optimum path. Protein Eng., 11, 739–747. 9796821 10.1093/protein/11.9.739

[btw554-B18] Sreerama N. et al (2000) Estimation of protein secondary structure from circular dichroism spectra: inclusion of denatured proteins with native proteins in the analysis. Anal. Biochem., 287, 243–251. 11112270 10.1006/abio.2000.4879

[btw554-B19] Whitmore L. et al (2011) PCDDB: the protein circular dichroism data bank, a repository for circular dichroism spectral and metadata. Nucleic Acids Res., 39, D480–D486. 21071417 10.1093/nar/gkq1026 PMC3013654

[btw554-B20] Whitmore L., Wallace B.A. (2008) Protein secondary structure analyses from circular dichroism spectroscopy: methods and reference databases. Biopolymers, 89, 392–400. 17896349 10.1002/bip.20853

[btw554-B21] Wiedemann C. et al (2013) CAPITO – a web server-based analysis and plotting tool for circular dichroism data. Bioinformatics, 29, 1750–1757. 23681122 10.1093/bioinformatics/btt278

[btw554-B22] Woollett B. et al (2013) ValiDichro: a website for validating and quality control of protein circular dichroism spectra. Nucleic Acids Res., 41, W417–W421. 23625965 10.1093/nar/gkt287 PMC3977657

