# Supplementary material for: PDB2CD: a web-based application for the generation of circular dichroism spectra from protein atomic coordinates
Source: Bioinformatics. 2016 Sep 20;33(1):56–63. doi: 10.1093/bioinformatics/btw554 (PMC5408769; doi:10.1093/bioinformatics/btw554)
Supplement: Supplementary Data [file btw554_supp.zip › 14_B_proteins.pdf]

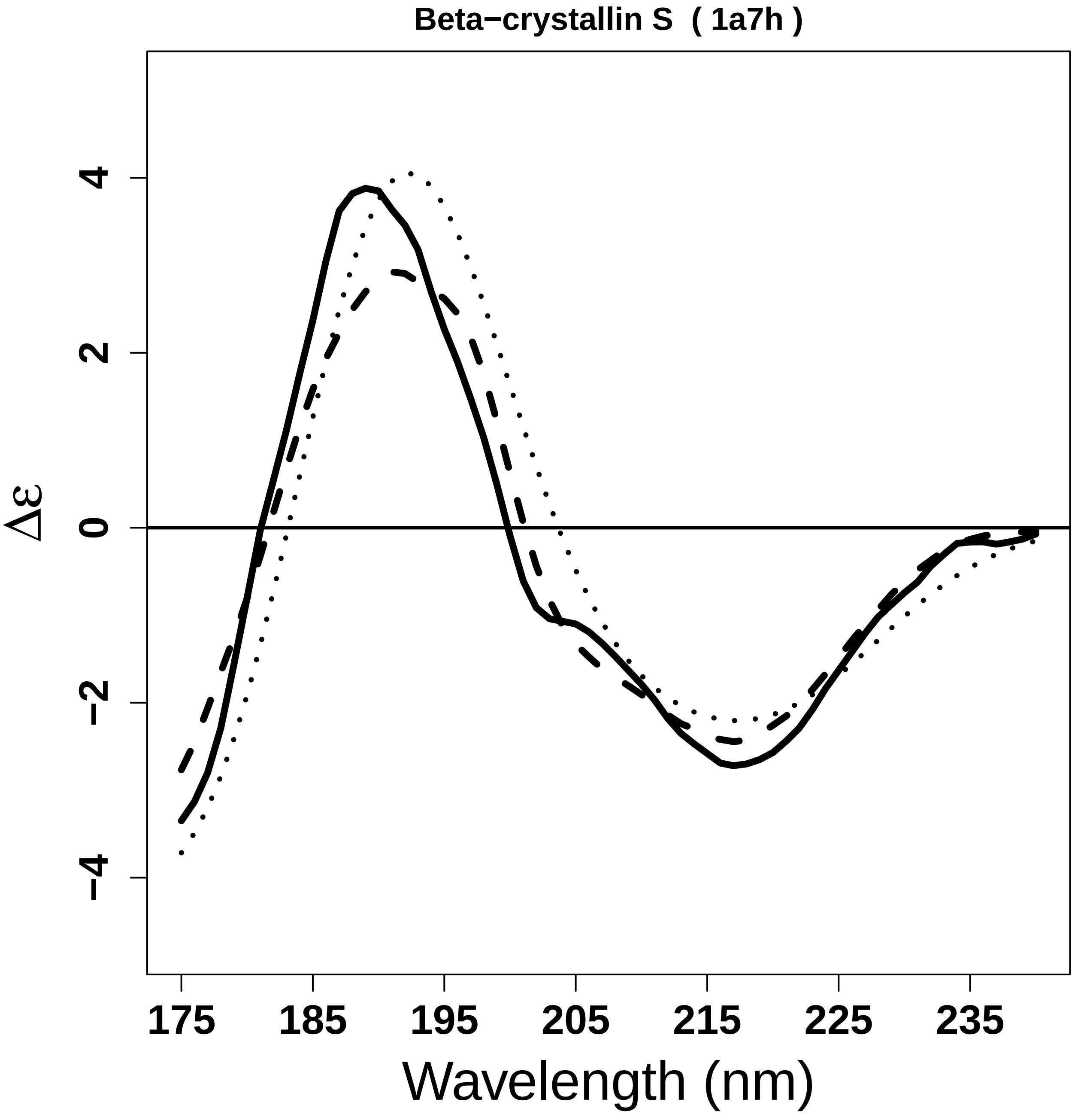

Beta−crystallin S (1a7h)
4
2
0
−2
−4
Δε
175
185
195
205
215
225
235
Wavelength (nm)

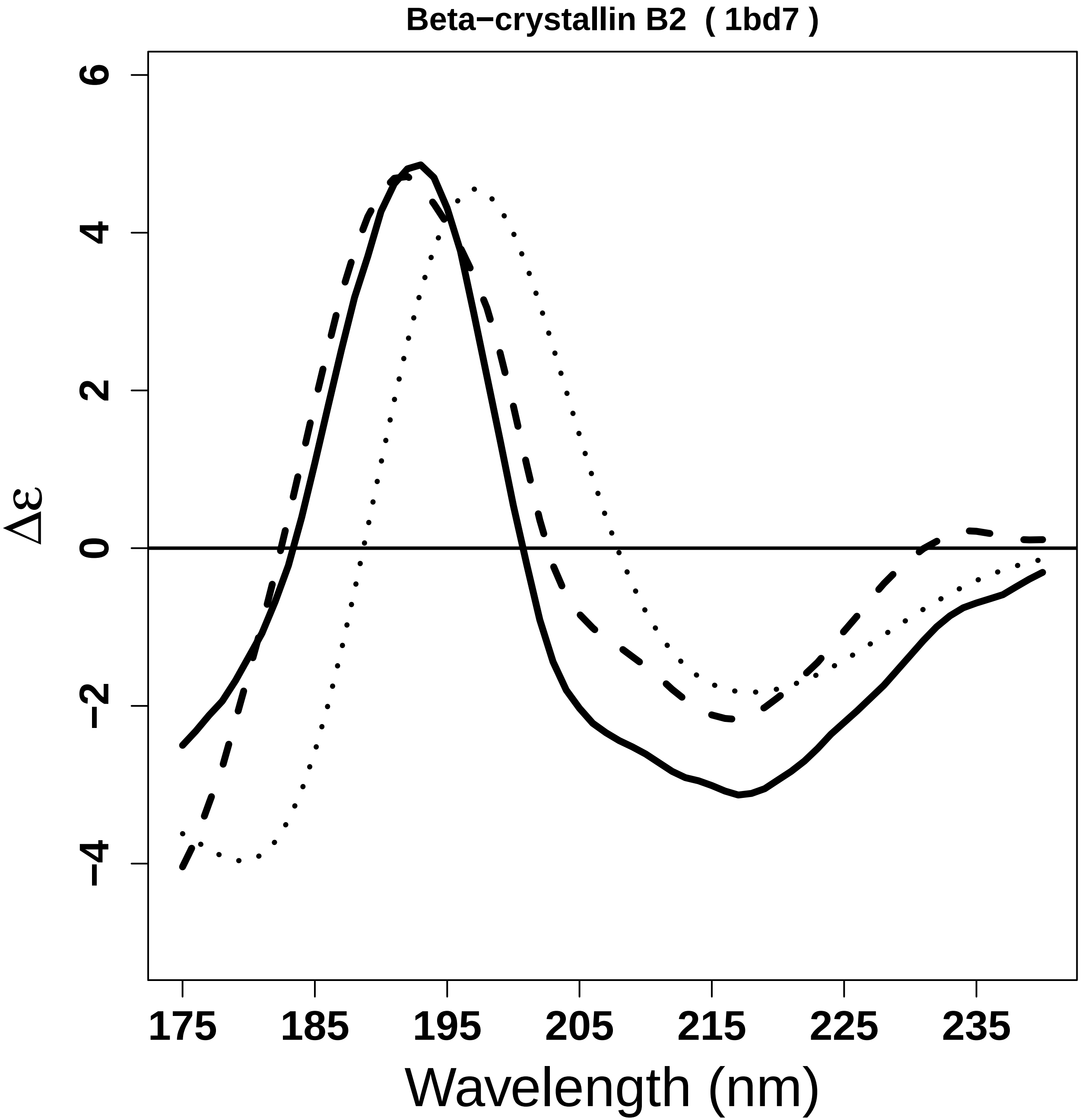

Beta−crystallin B2 (1bd7)
6
4
2
0
−2
−4
Δε
175
185
195
205
215
225
235
Wavelength (nm)

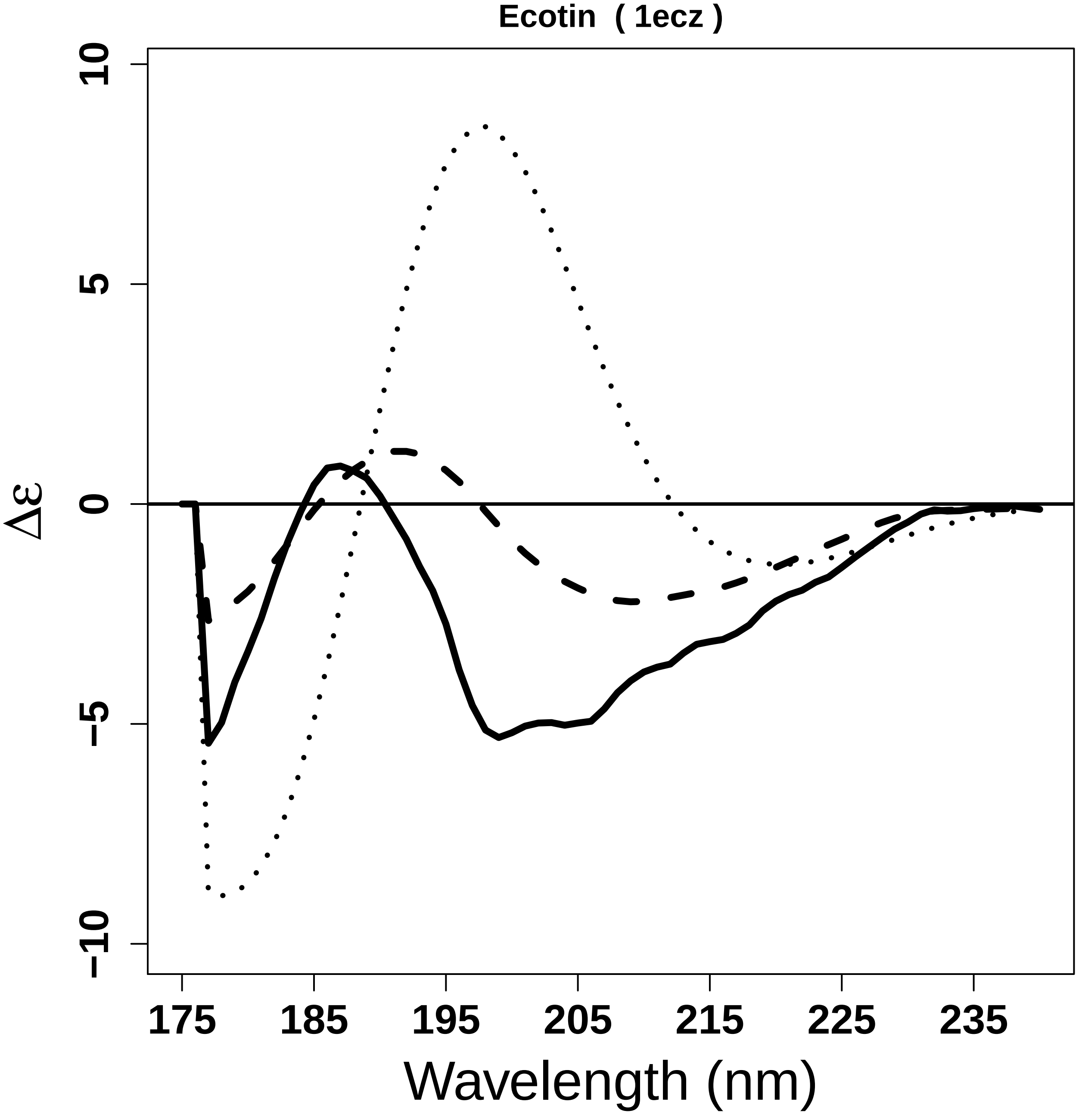

Ecotin (1ecz)
10
5
0
−5
−10
Δε
175
185
195
205
215
225
235
Wavelength (nm)

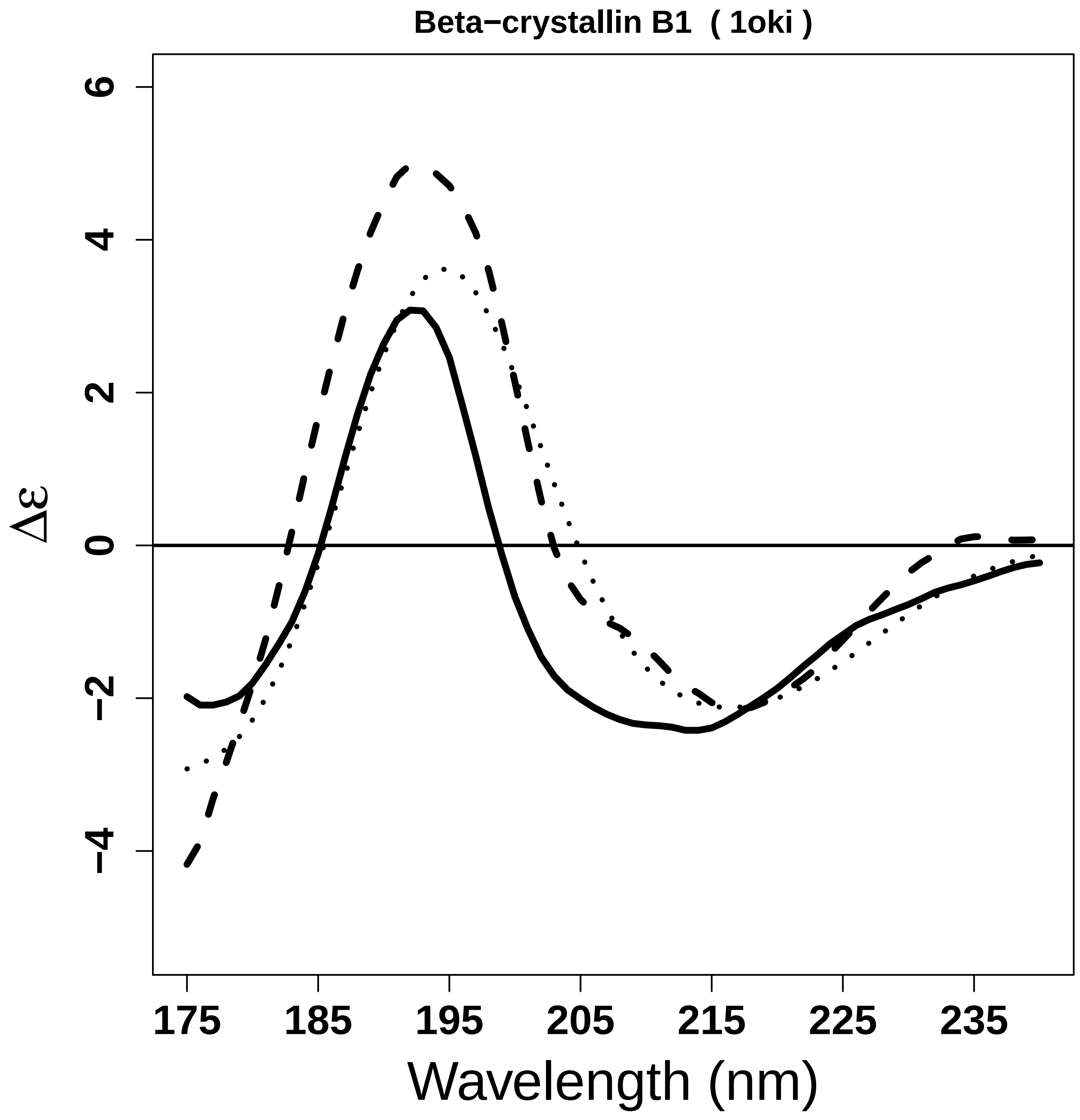

Beta−crystallin B1 (1oki)
Δε
6
4
2
0
−2
−4
175
185
195
205
215
225
235
Wavelength (nm)

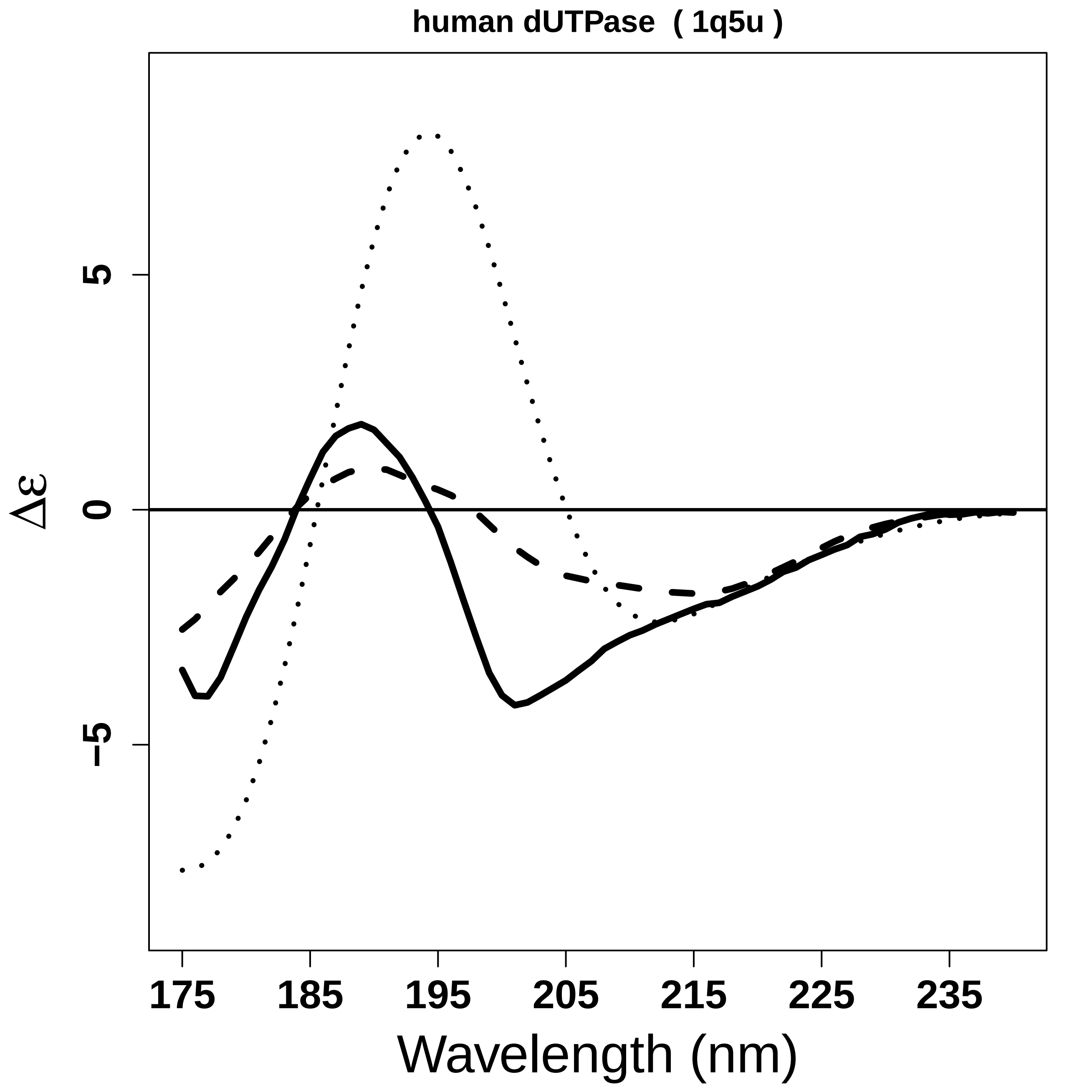
human dUTPase (1q5u)
Δε
5
0
−5
175
185
195
205
215
225
235
Wavelength (nm)

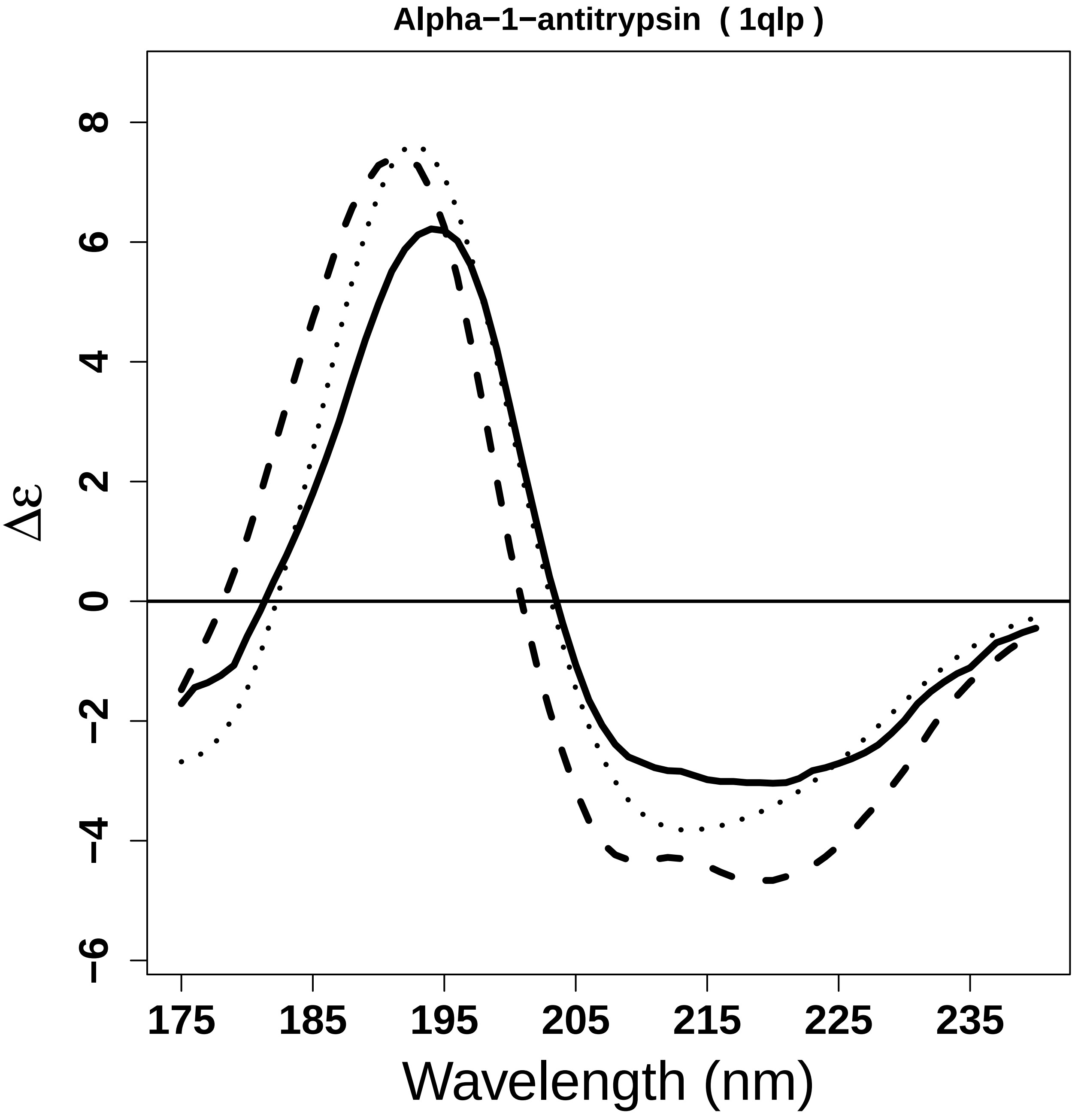

Alpha−1−antitrypsin (1qlp)
Δε
8
6
4
2
0
−2
−4
−6
175
185
195
205
215
225
235
Wavelength (nm)

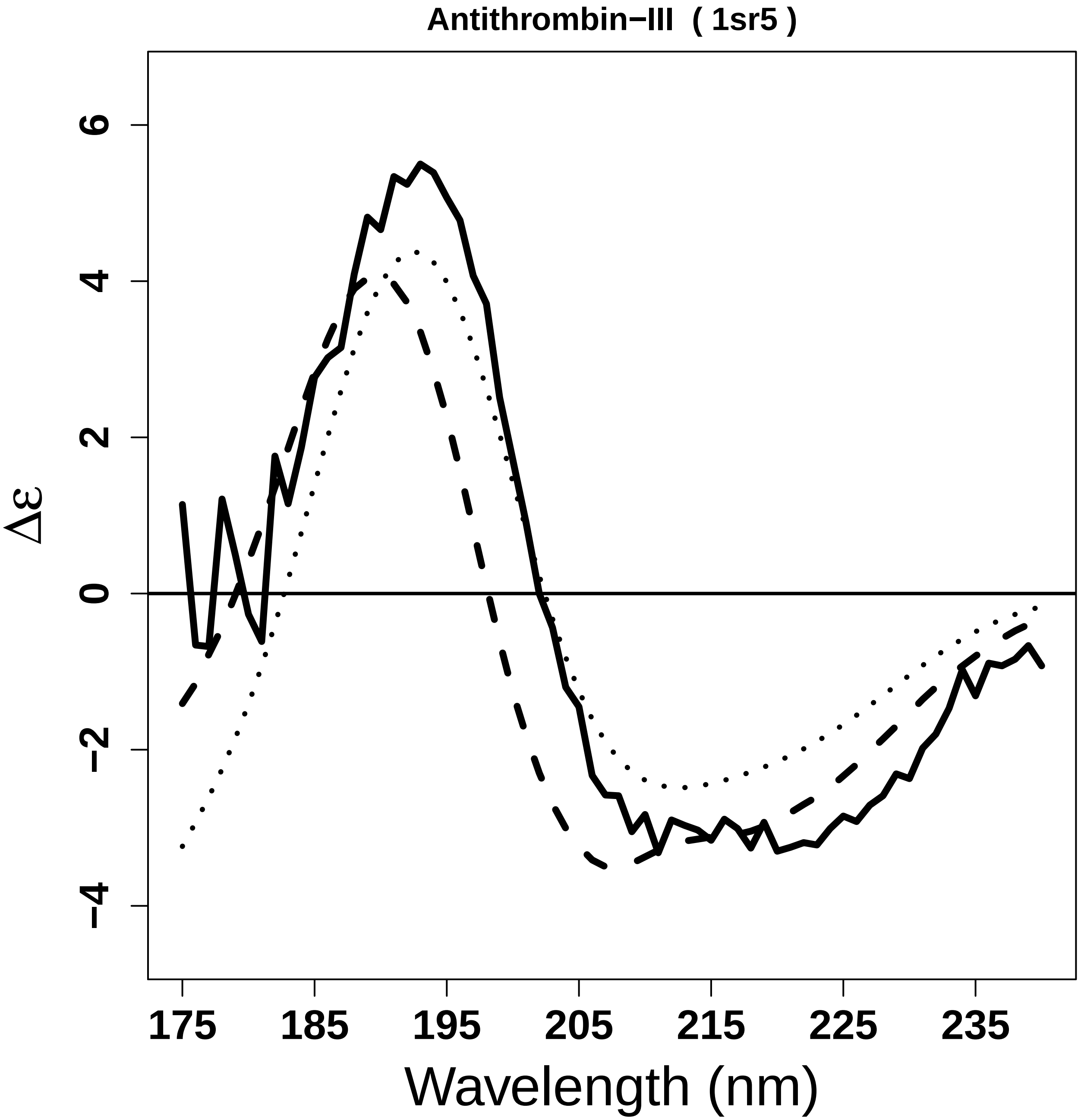

Antithrombin−III (1sr5)
Δε
6
4
2
0
−2
−4
175
185
195
205
215
225
235
Wavelength (nm)

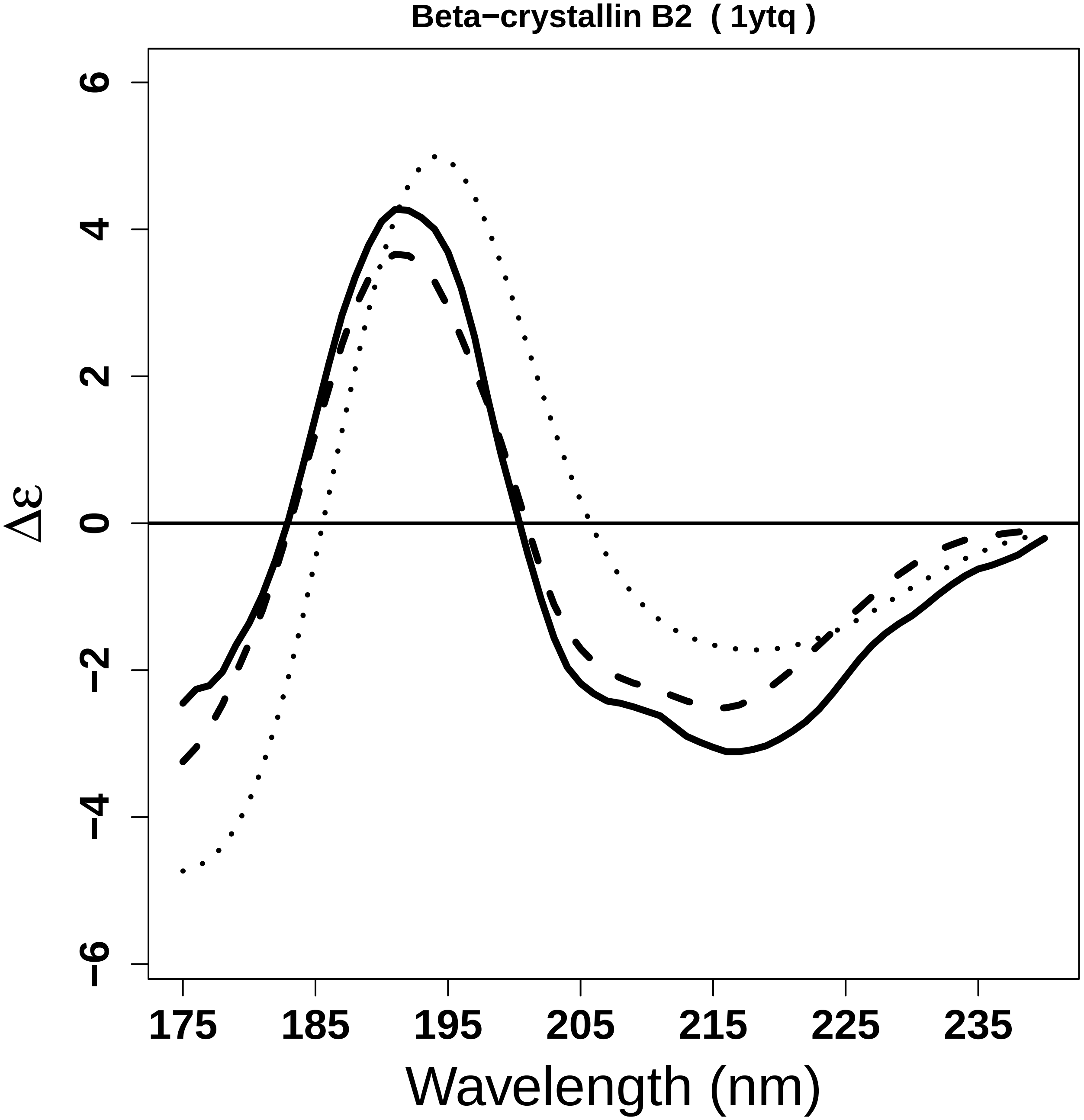
Beta−crystallin B2 (1ytq)
Δε
6
4
2
0
−2
−4
−6
175
185
195
205
215
225
235
Wavelength (nm)

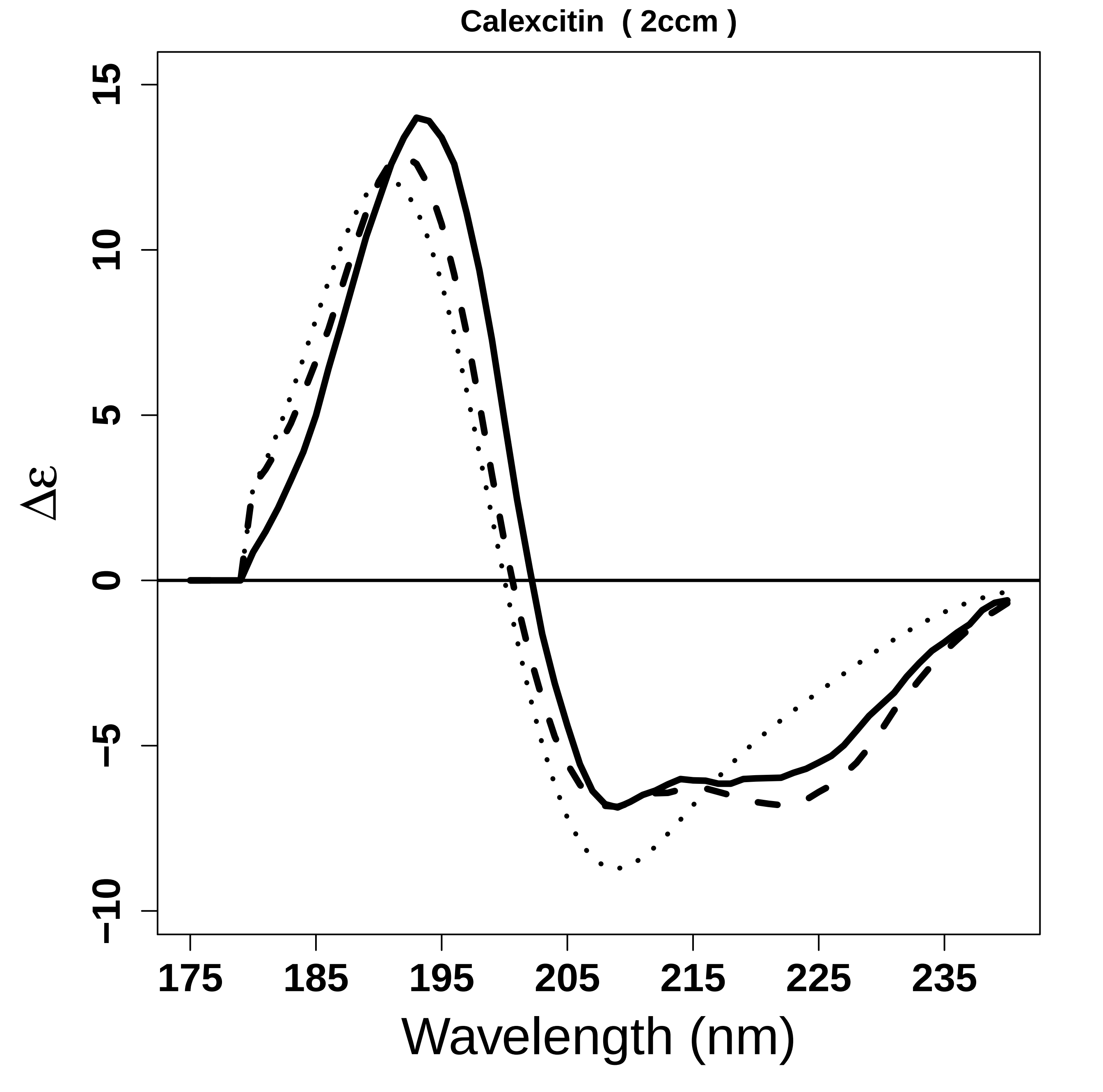

Calexcitin (2ccm)
15
10
5
0
−5
−10
Δε
175
185
195
205
215
225
235
Wavelength (nm)

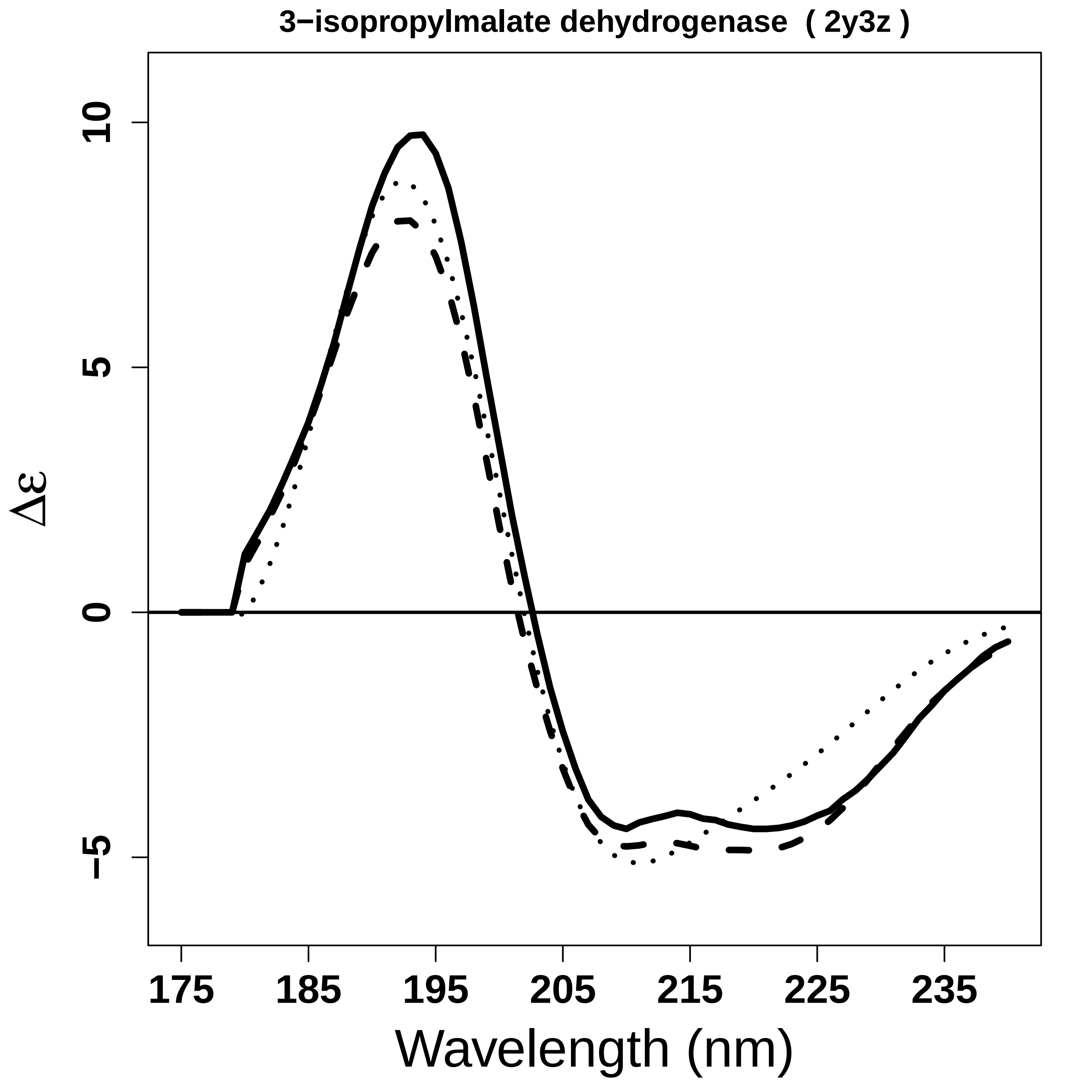

3−isopropylmalate dehydrogenase (2y3z)
10
5
0
−5
Δε
175
185
195
205
215
225
235
Wavelength (nm)

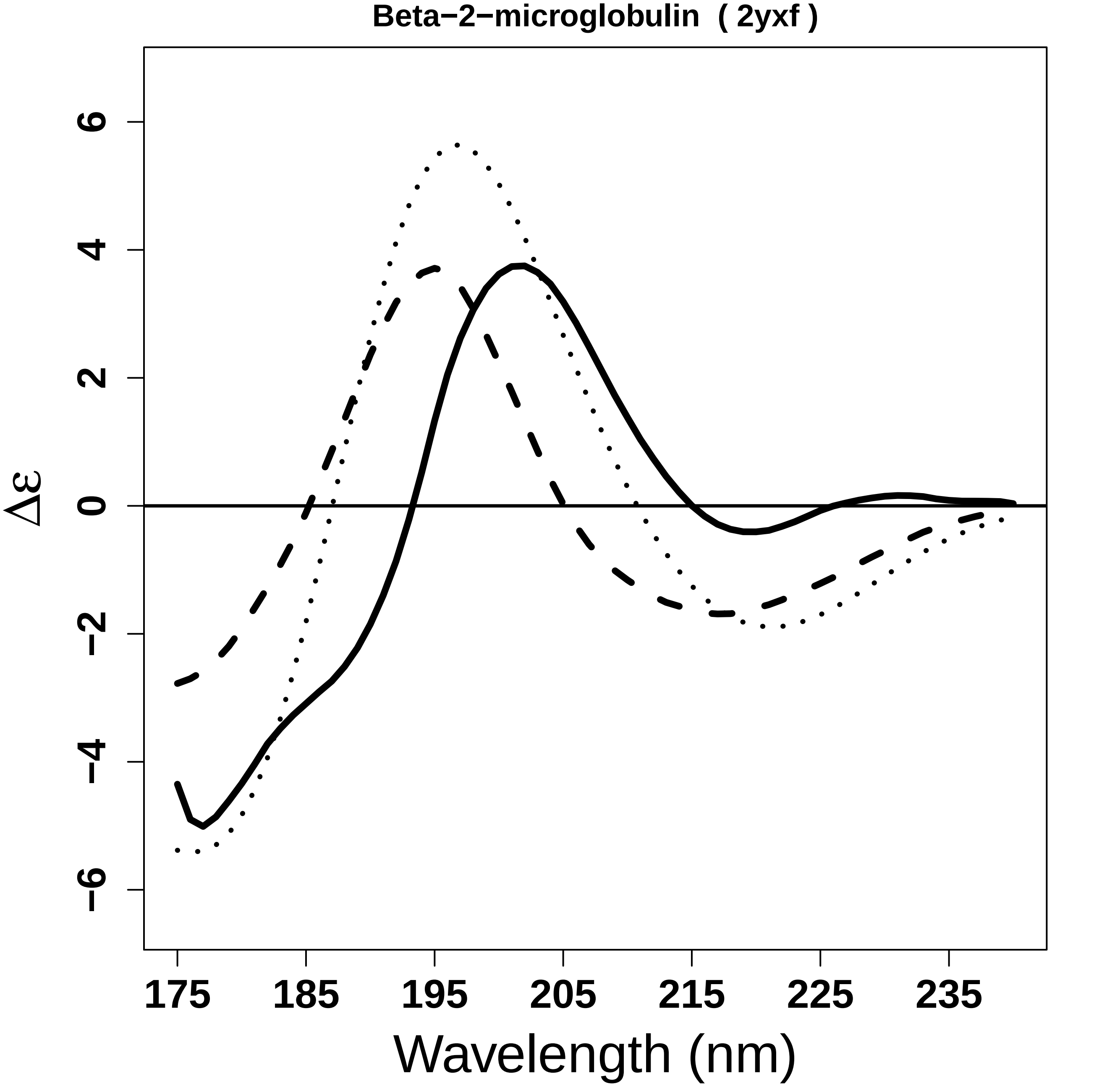

Beta−2−microglobulin (2yxf)
Δε
6
4
2
0
−2
−4
−6
175
185
195
205
215
225
235
Wavelength (nm)

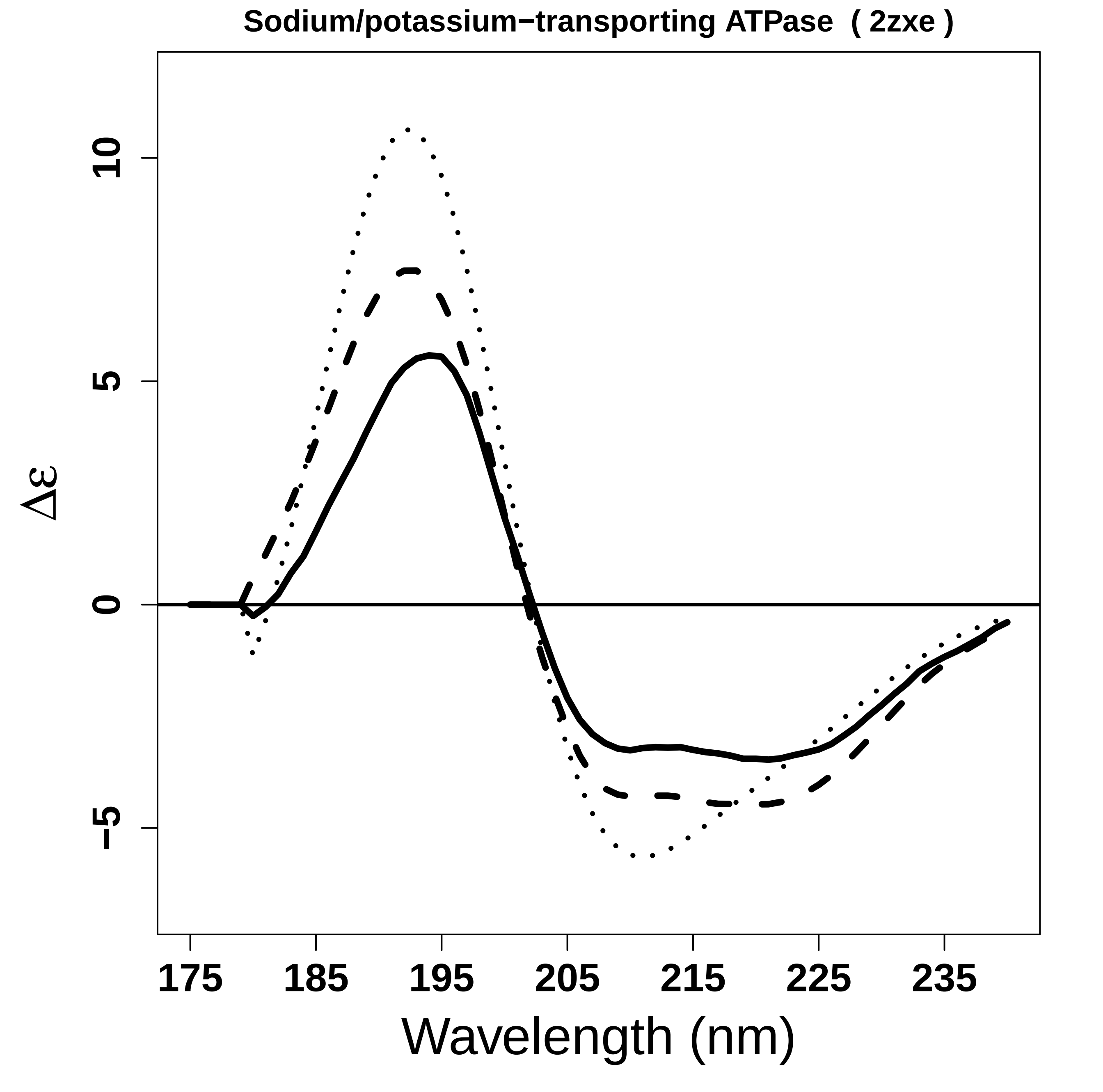
Sodium/potassium−transporting ATPase (2zxe)
10
5
0
−5
Δε
175
185
195
205
215
225
235
Wavelength (nm)

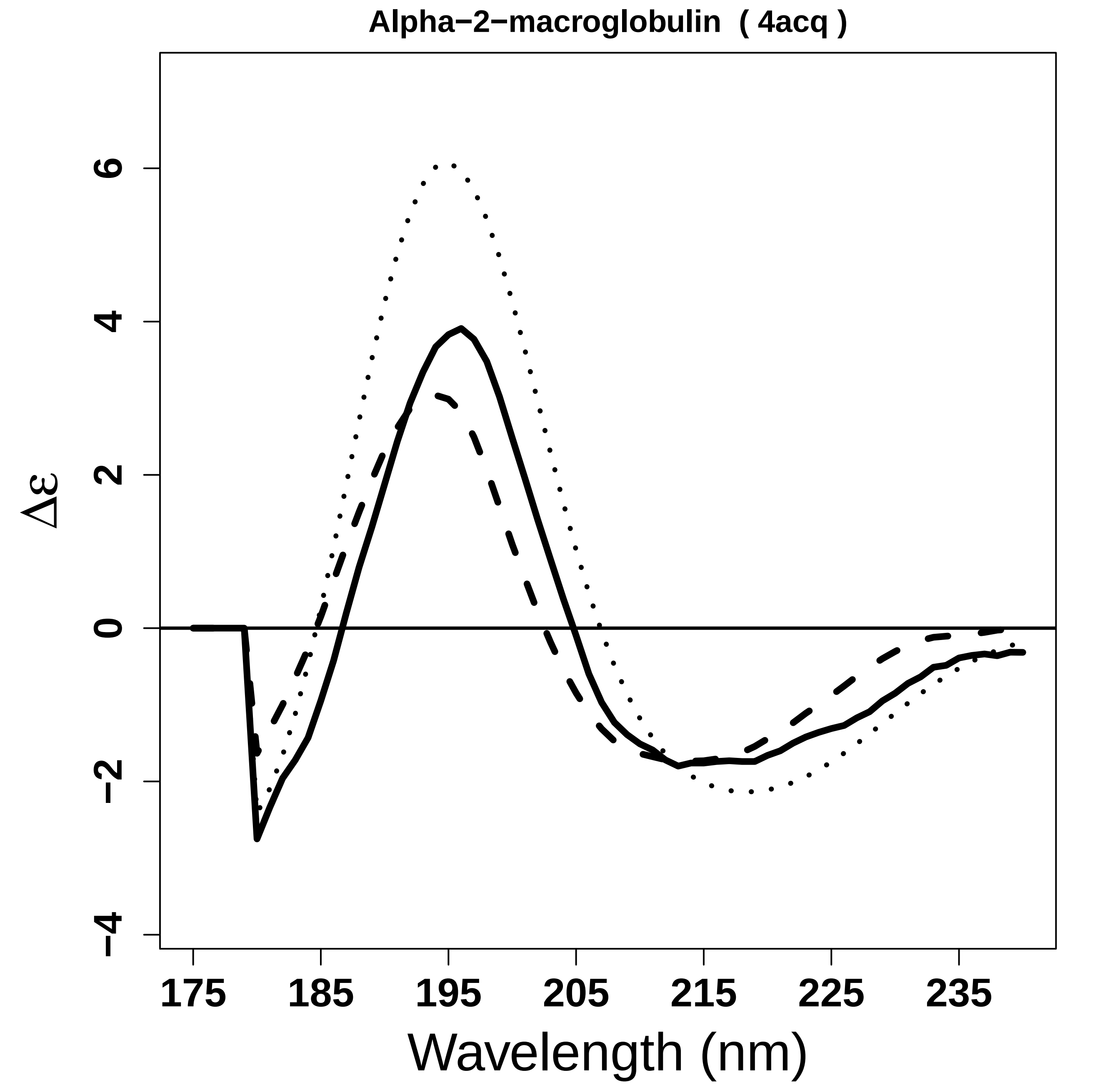

Alpha−2−macroglobulin (4acq)
Δε
−4
−2
0
2
4
6
175
185
195
205
215
225
235
Wavelength (nm)

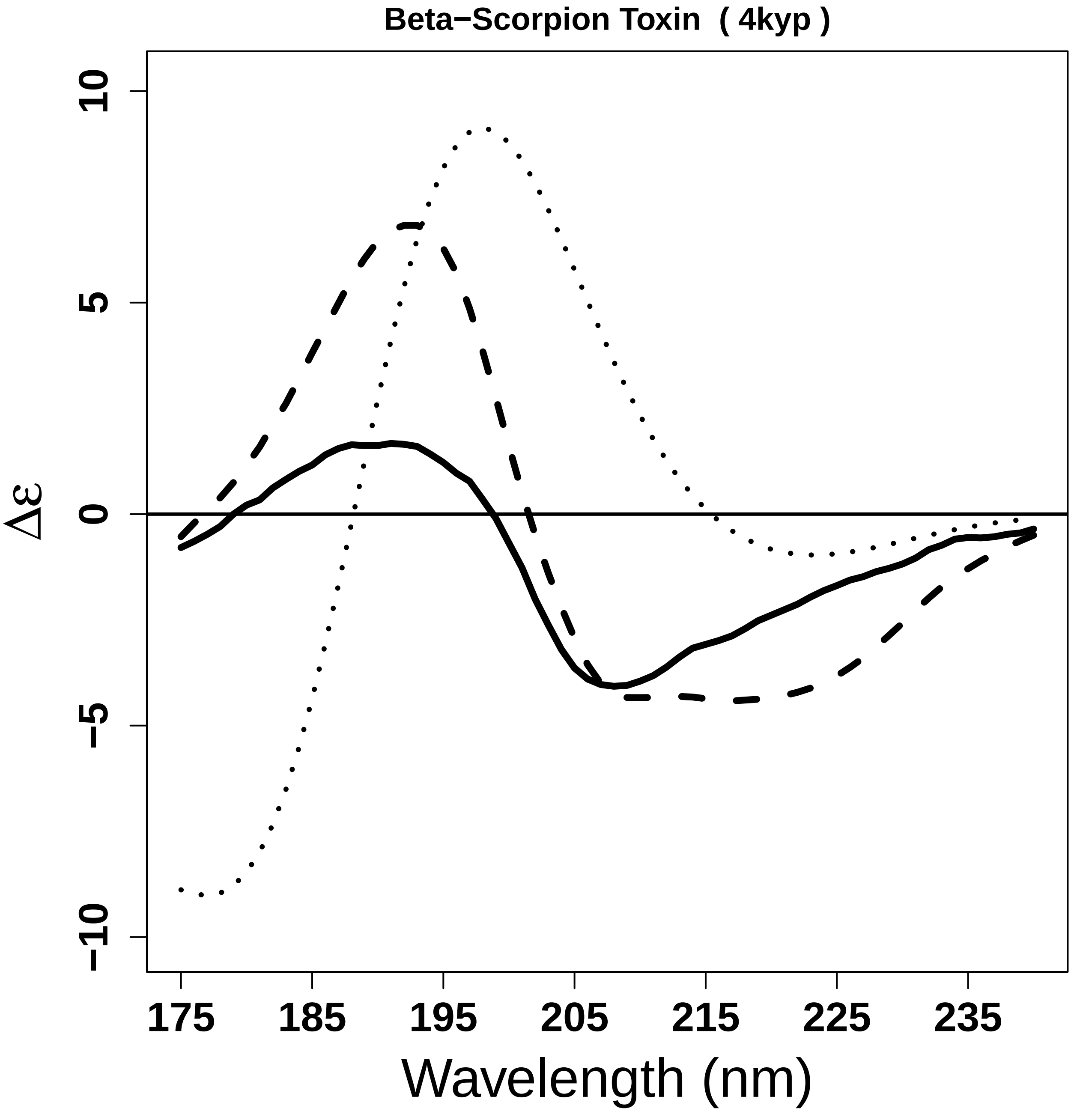

Beta−Scorpion Toxin (4kyp)
10
5
0
−5
−10
Δε
175
185
195
205
215
225
235
Wavelength (nm)
