# Supplementary material for: PDB2CD: a web-based application for the generation of circular dichroism spectra from protein atomic coordinates
Source: Bioinformatics. 2016 Sep 20;33(1):56–63. doi: 10.1093/bioinformatics/btw554 (PMC5408769; doi:10.1093/bioinformatics/btw554)
Supplement: Supplementary Data [file btw554_supp.zip › 71_B_proteins.pdf]

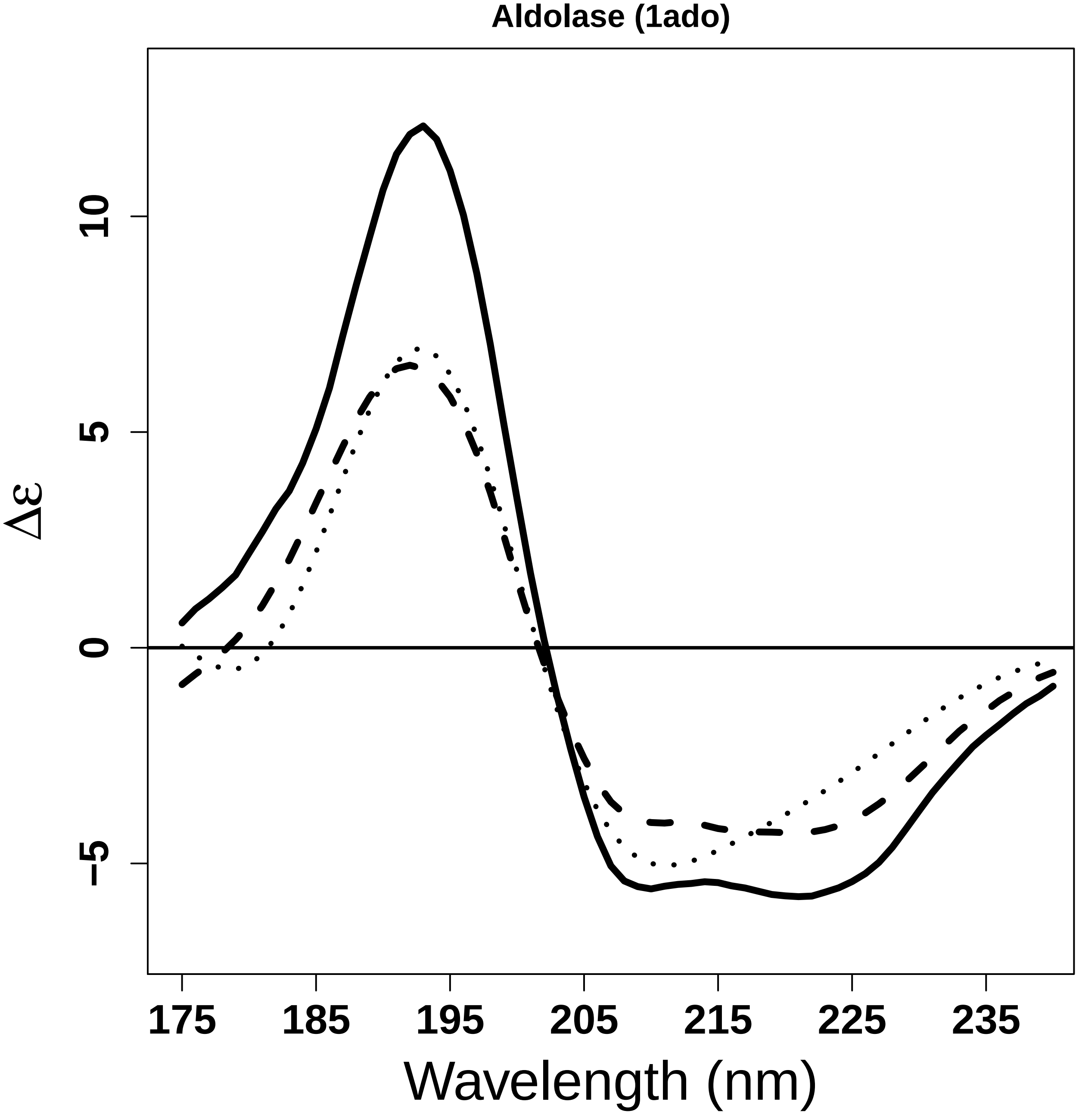

Aldolase (1ado)
10
5
0
−5
Δε
175
185
195
205
215
225
235
Wavelength (nm)

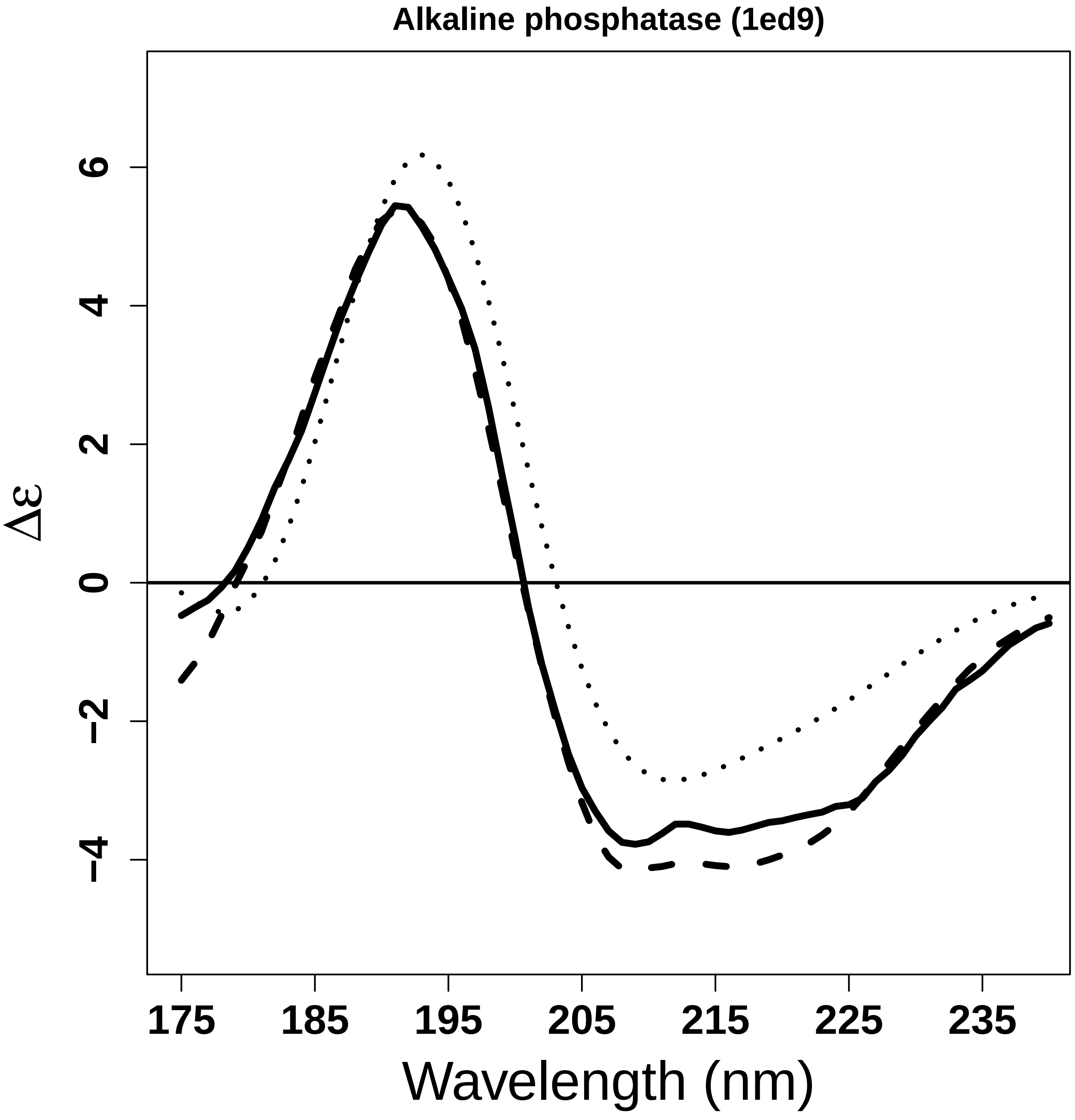

Alkaline phosphatase (1ed9)
Δε
6
4
2
0
−2
−4
175
185
195
205
215
225
235
Wavelength (nm)

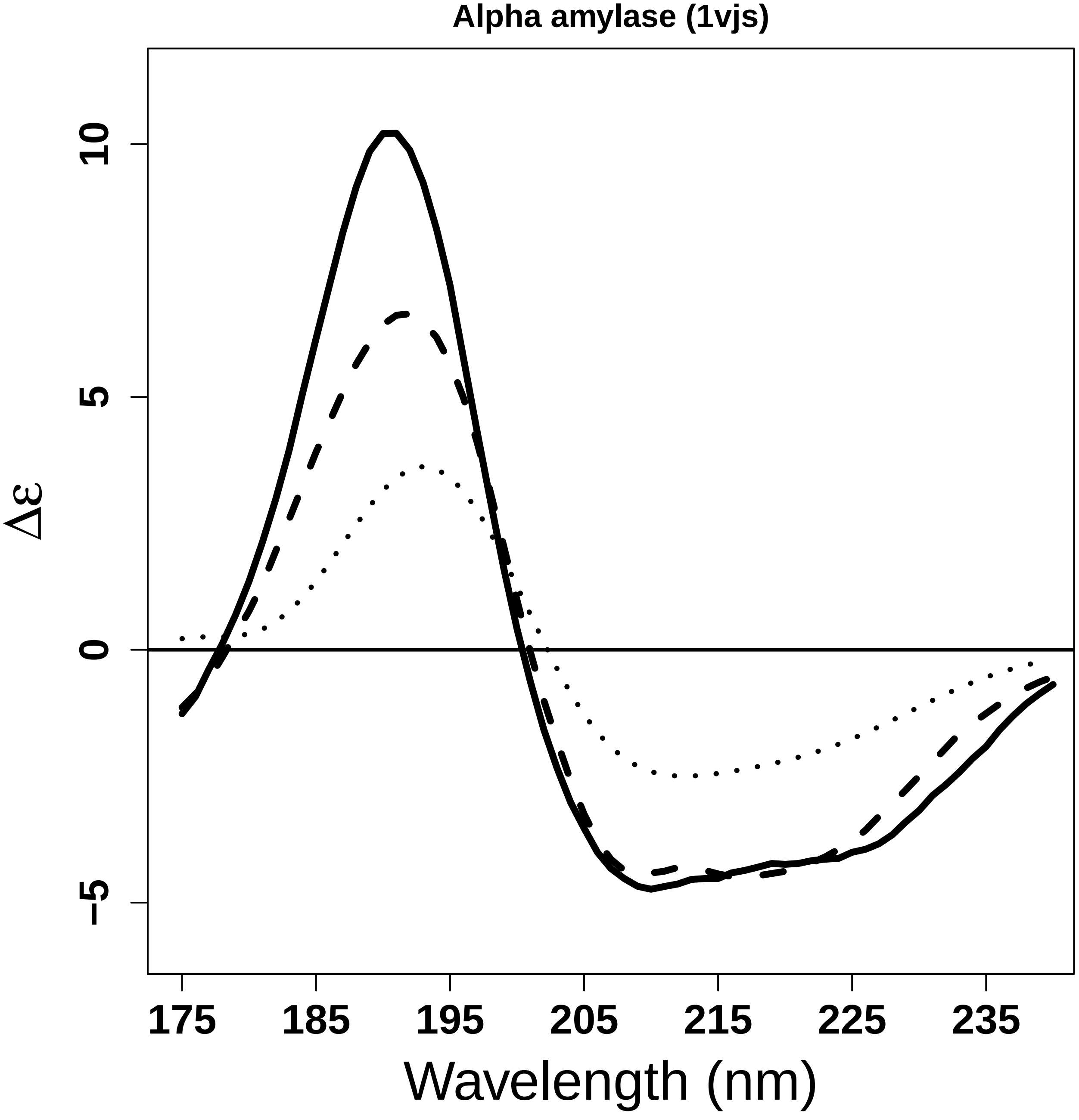

Alpha amylase (1vjs)
10
5
0
−5
Δε
175
185
195
205
215
225
235
Wavelength (nm)

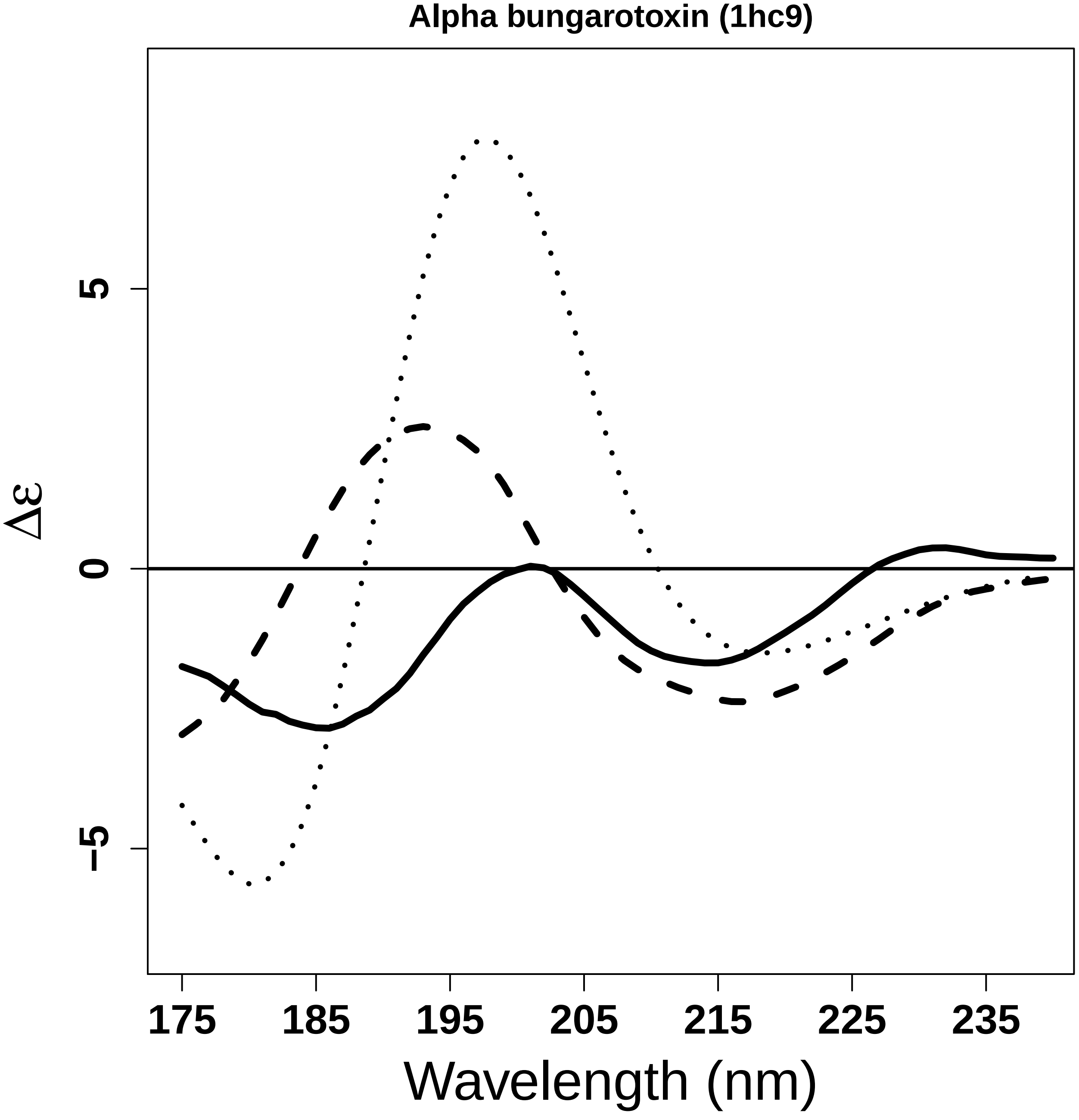

Alpha bungarotoxin (1hc9)
5
0
−5
Δε
175
185
195
205
215
225
235
Wavelength (nm)

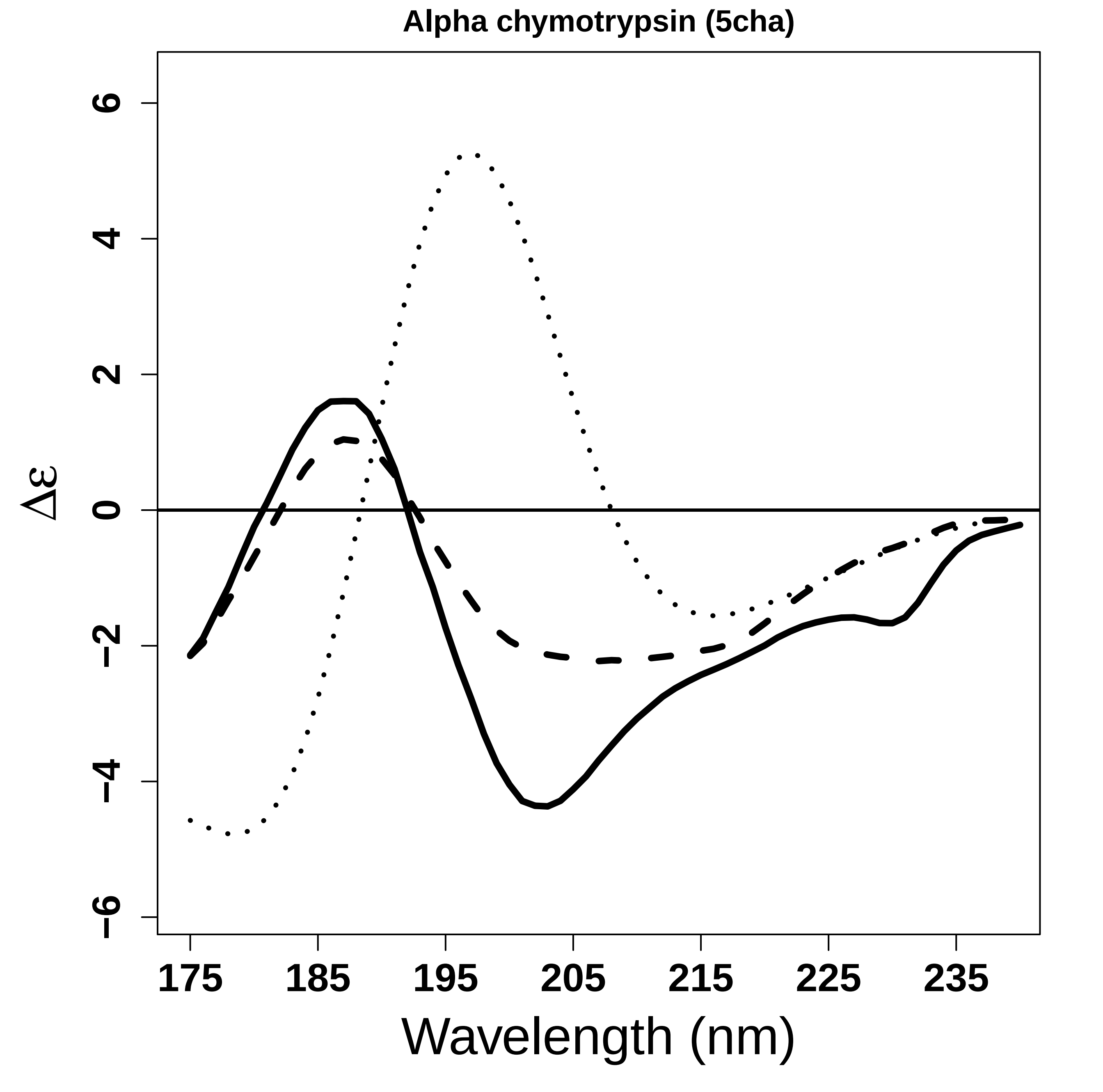

Alpha chymotrypsin (5cha)
Δε
6
4
2
0
−2
−4
−6
175
185
195
205
215
225
235
Wavelength (nm)

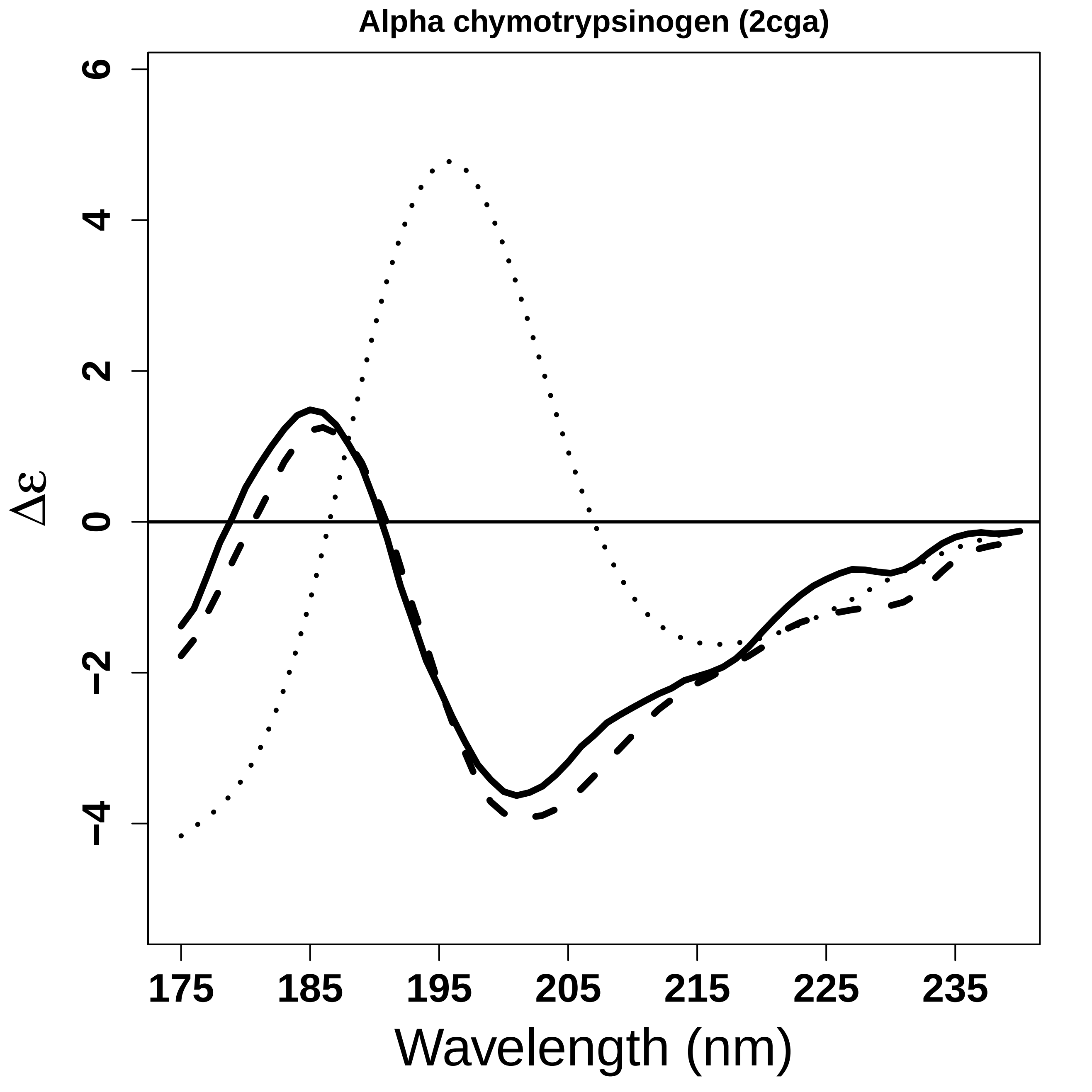

Alpha chymotrypsinogen (2cga)
6
4
2
0
−2
−4
Δε
175
185
195
205
215
225
235
Wavelength (nm)

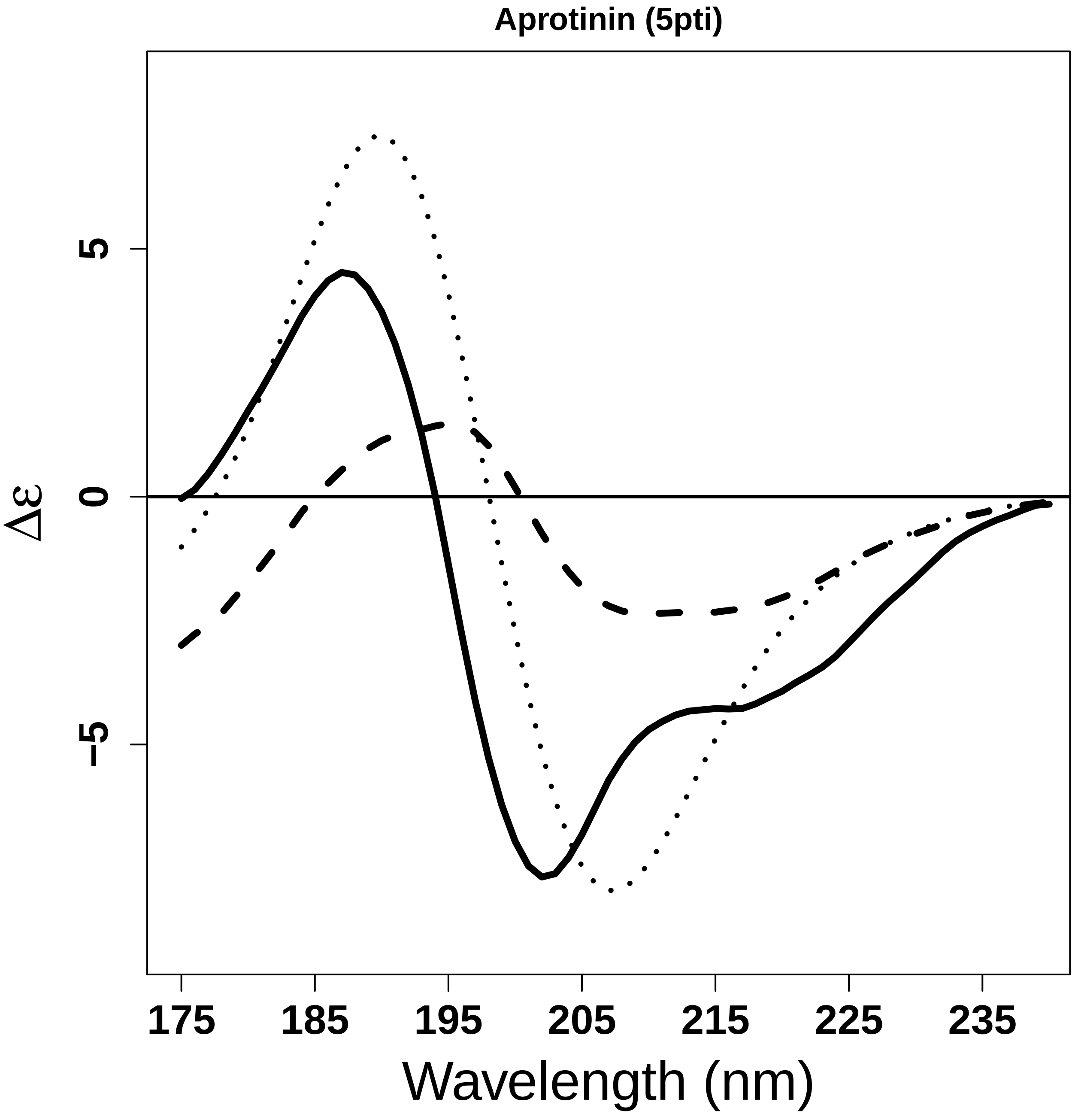

Aprotinin (5pti)
Δε
5
0
−5
175
185
195
205
215
225
235
Wavelength (nm)

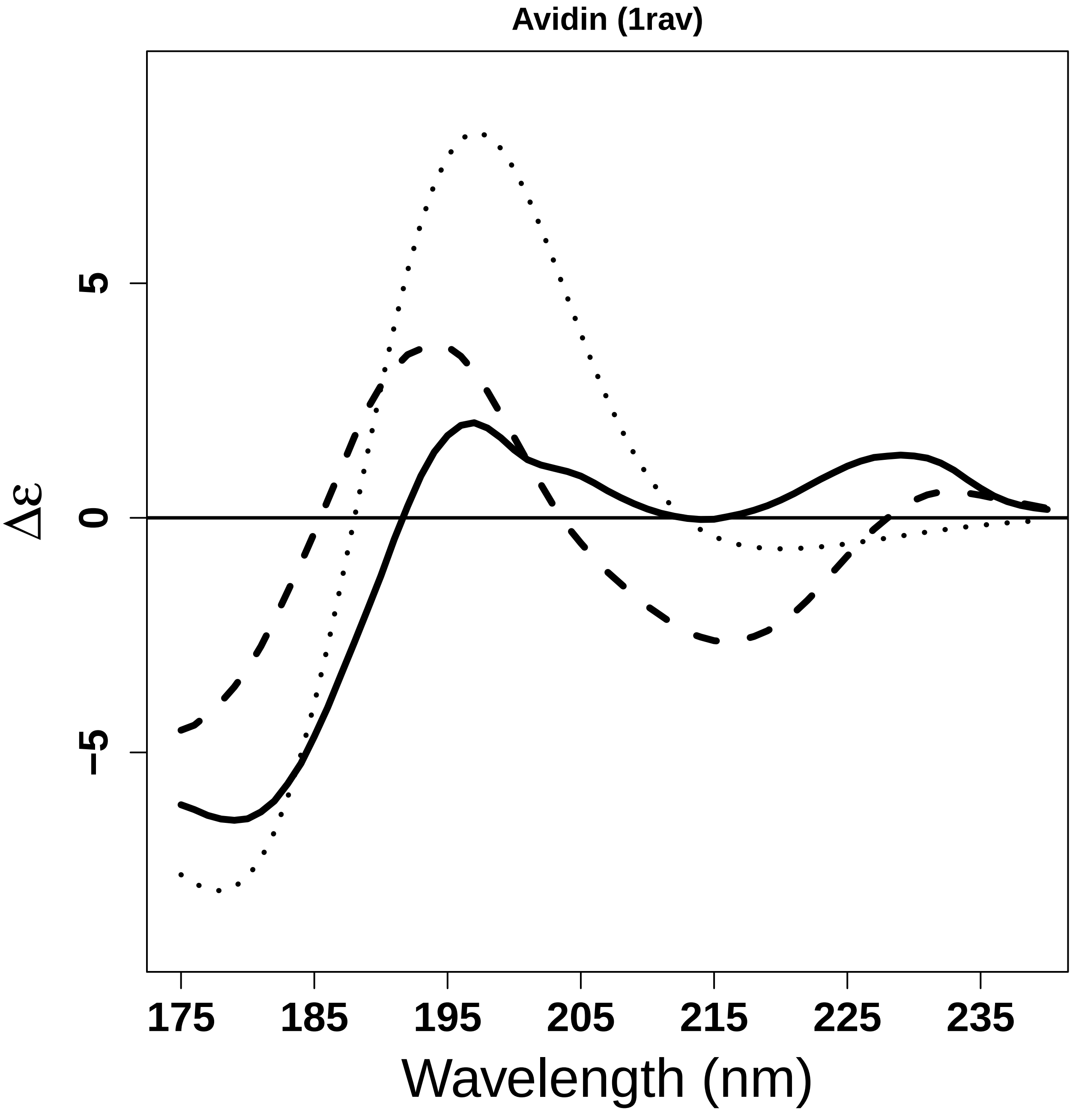

Avidin (1rav)
5
0
−5
Δε
175
185
195
205
215
225
235
Wavelength (nm)

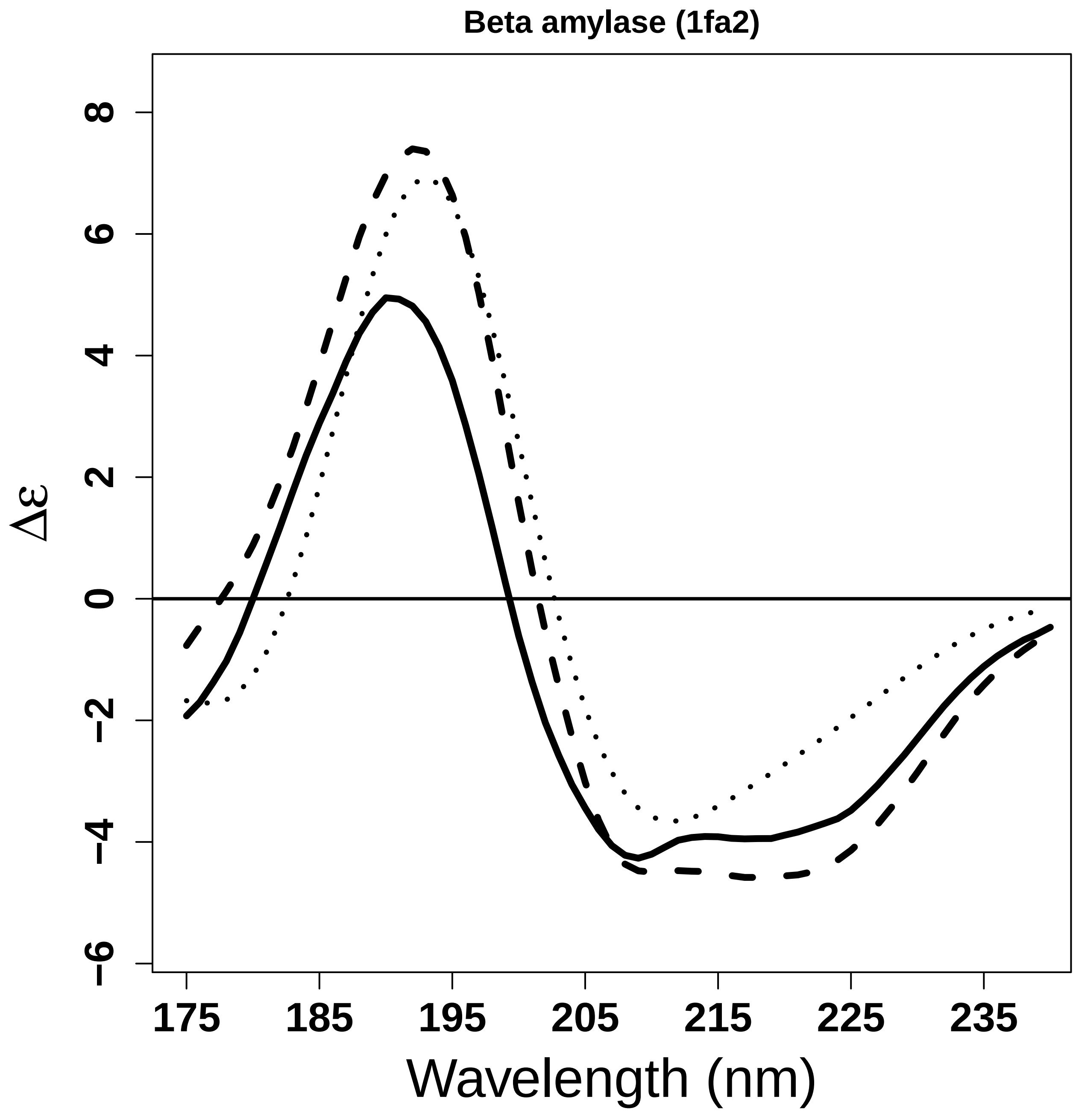

Beta amylase (1fa2)
Δε
8
6
4
2
0
−2
−4
−6
175
185
195
205
215
225
235
Wavelength (nm)

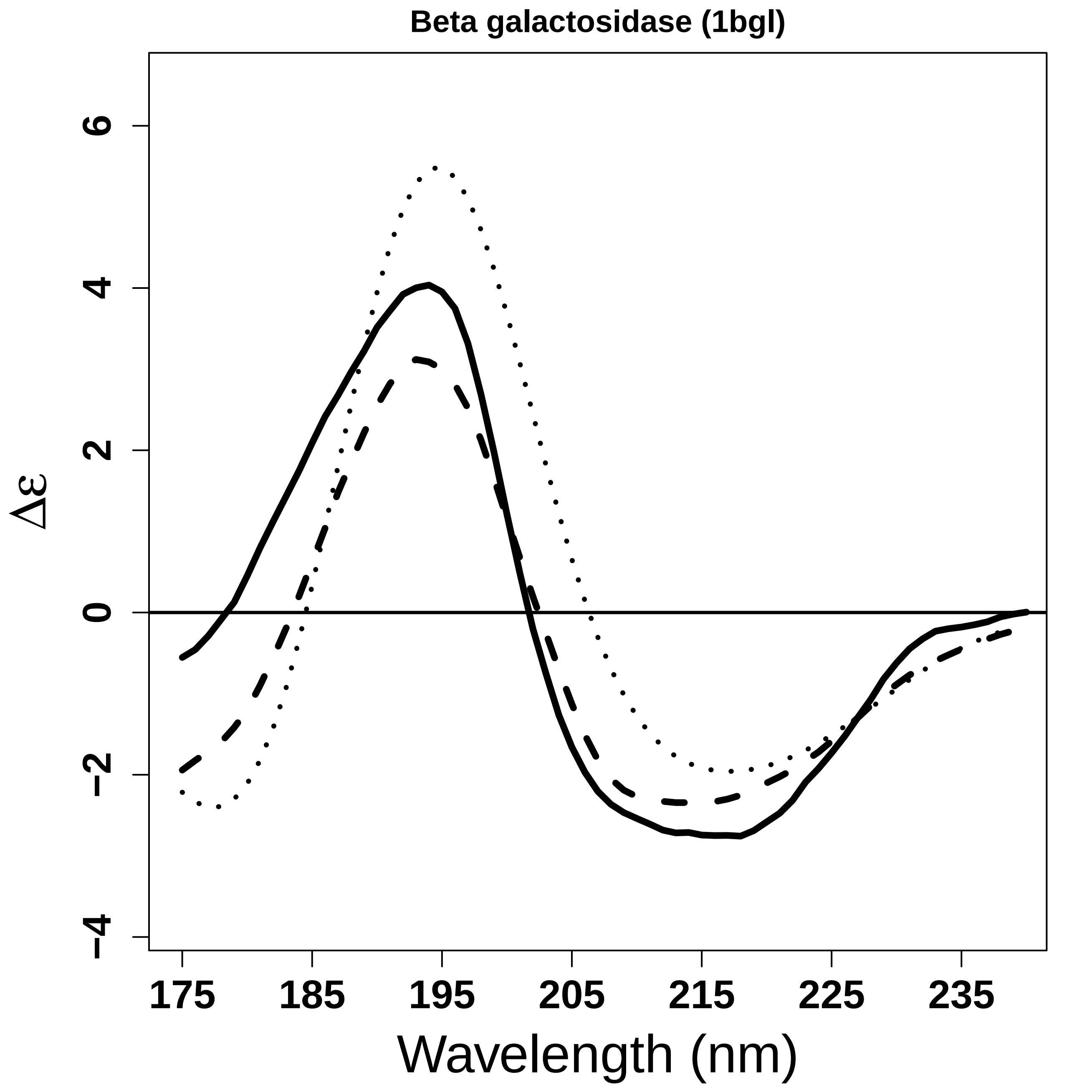

Beta galactosidase (1bgl)
Δε
6
4
2
0
−2
−4
175
185
195
205
215
225
235
Wavelength (nm)

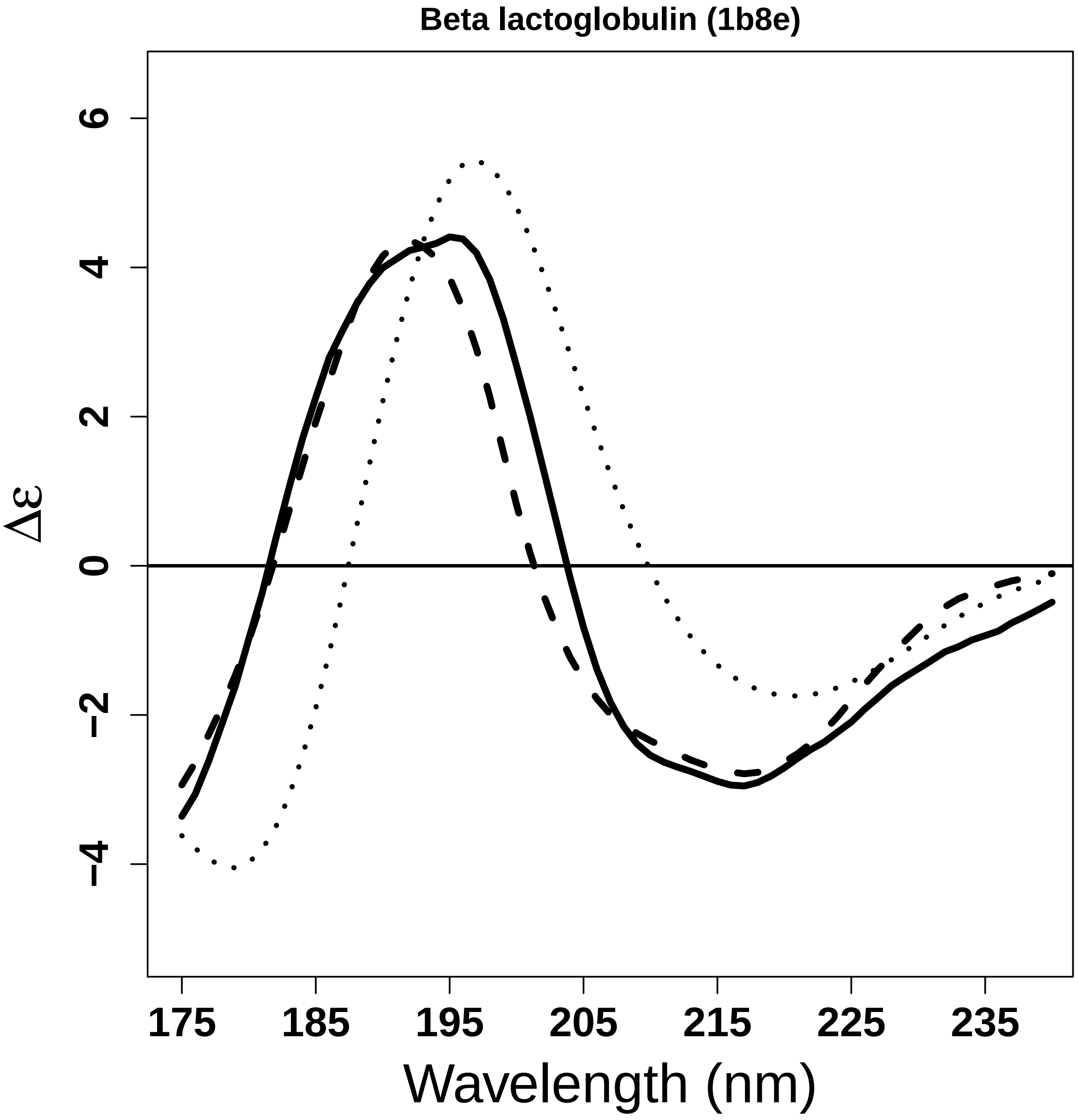

Beta lactoglobulin (1b8e)
Δε
6
4
2
0
−2
−4
175
185
195
205
215
225
235
Wavelength (nm)

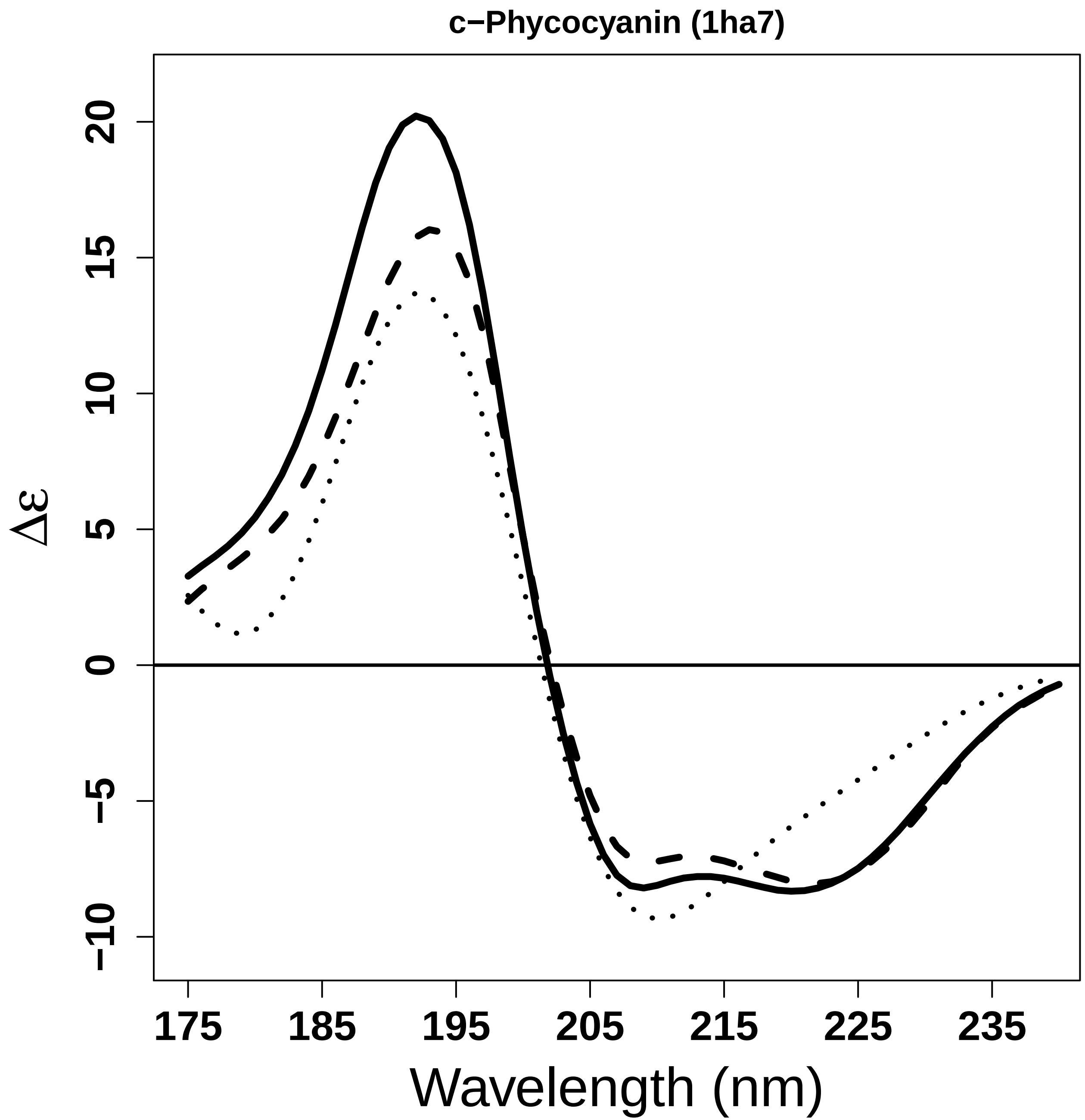

c−Phycocyanin (1ha7)
Δε
20
15
10
5
0
−5
−10
175
185
195
205
215
225
235
Wavelength (nm)

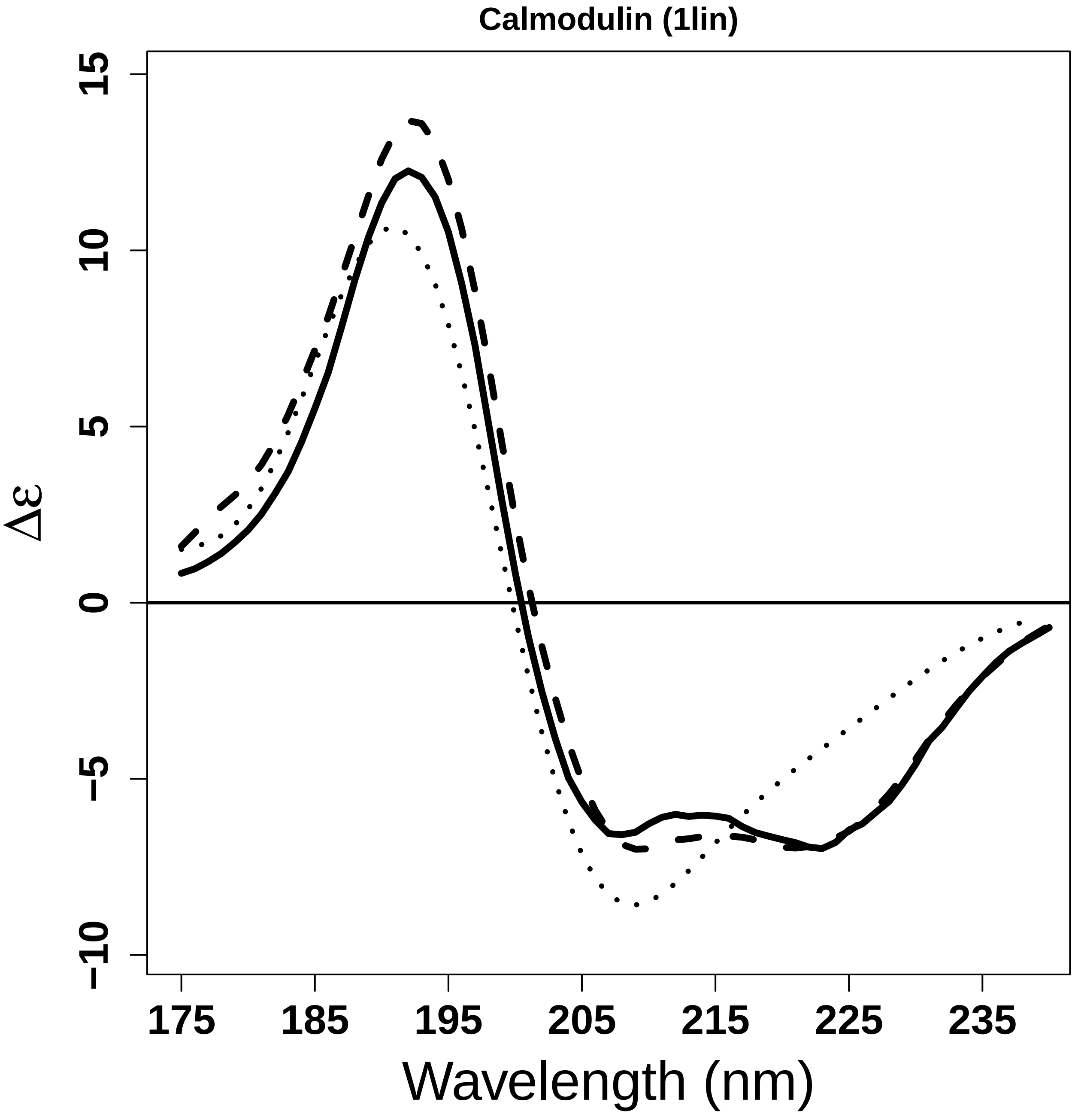
Calmodulin (1lin)
Δε
15
10
5
0
−5
−10
175
185
195
205
215
225
235
Wavelength (nm)

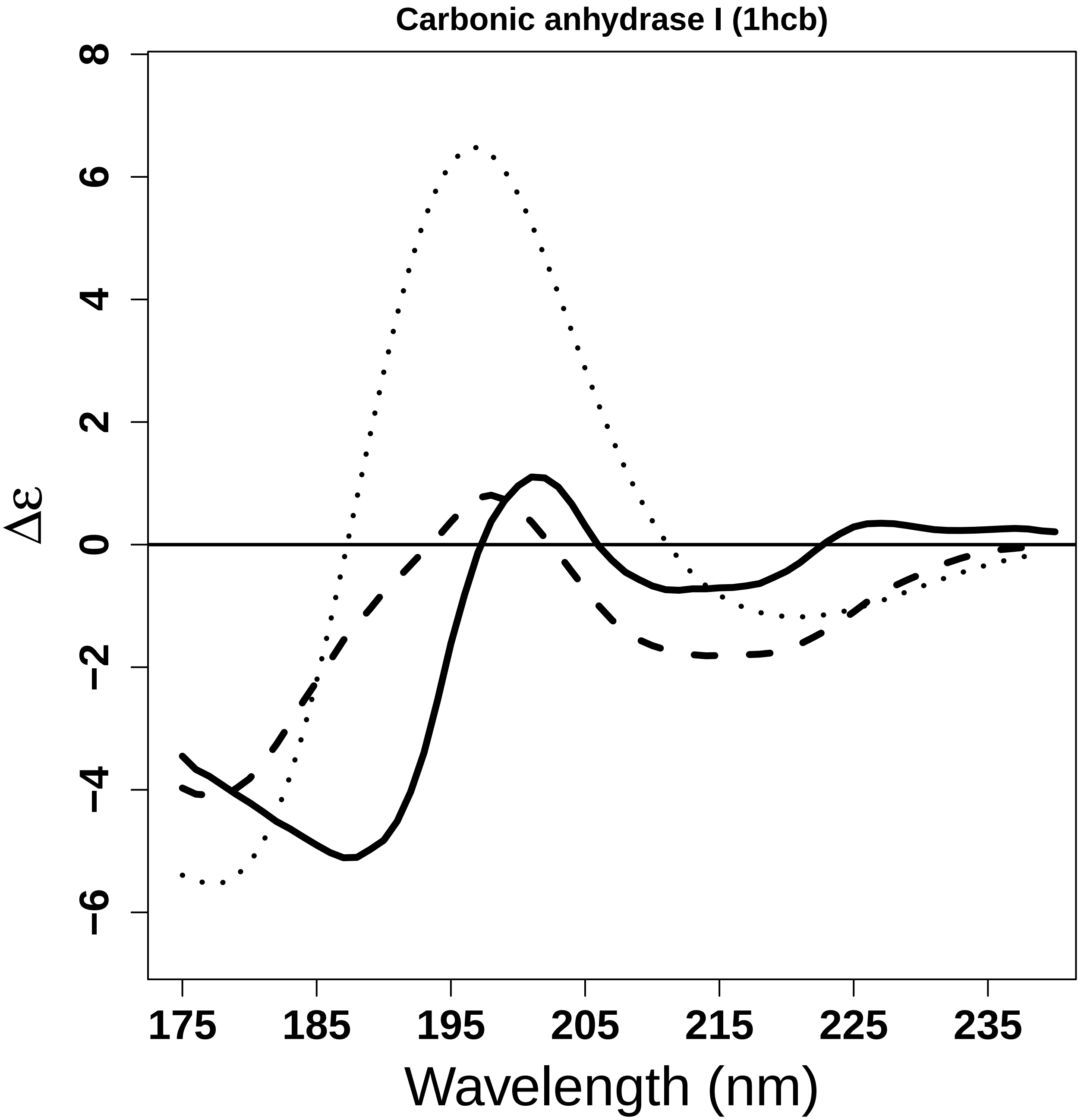

Carbonic anhydrase I (1hcb)
8
6
4
2
0
−2
−4
−6
Δε
175
185
195
205
215
225
235
Wavelength (nm)

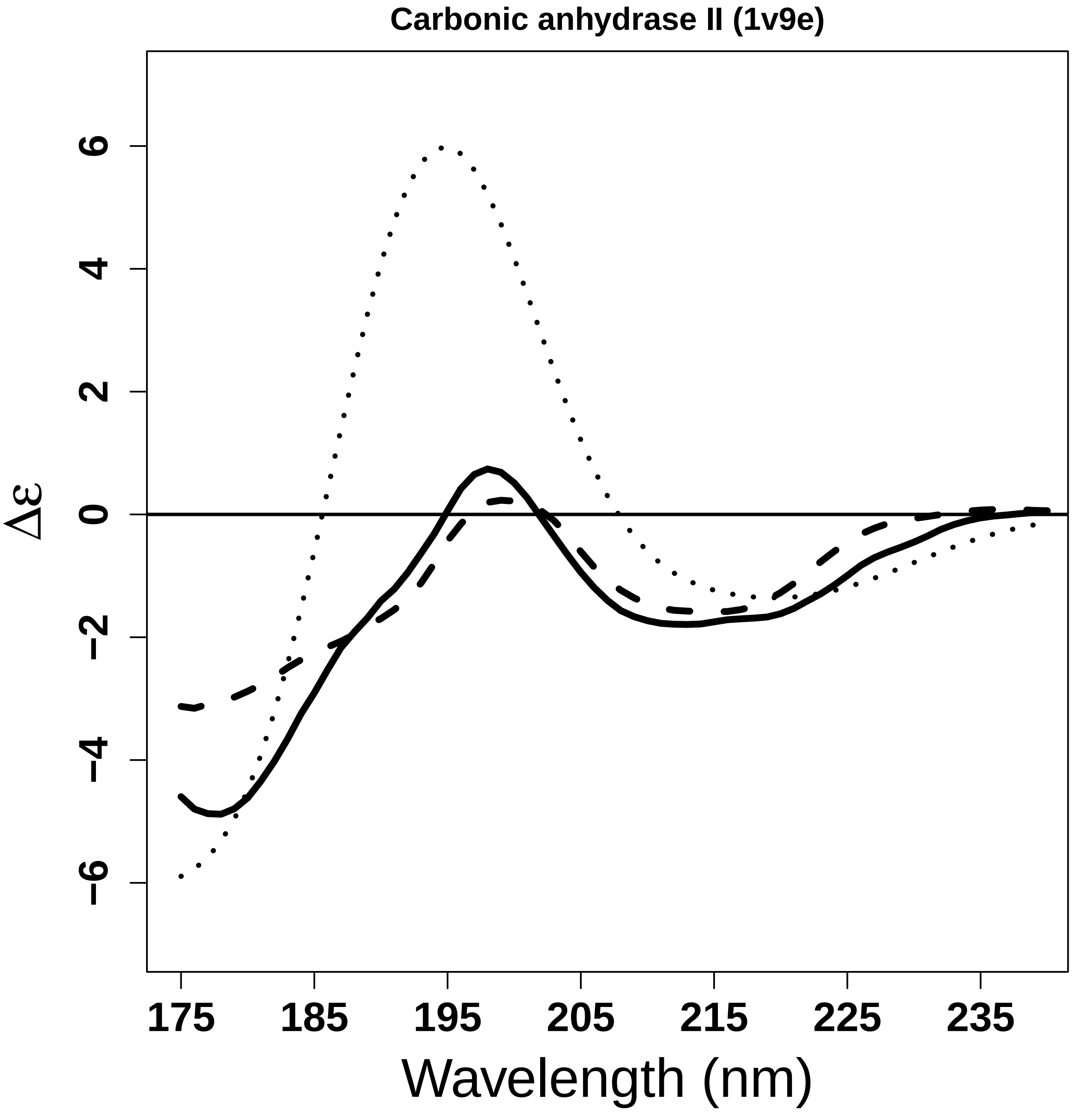

Carbonic anhydrase II (1v9e)
Δε
6
4
2
0
−2
−4
−6
175
185
195
205
215
225
235
Wavelength (nm)

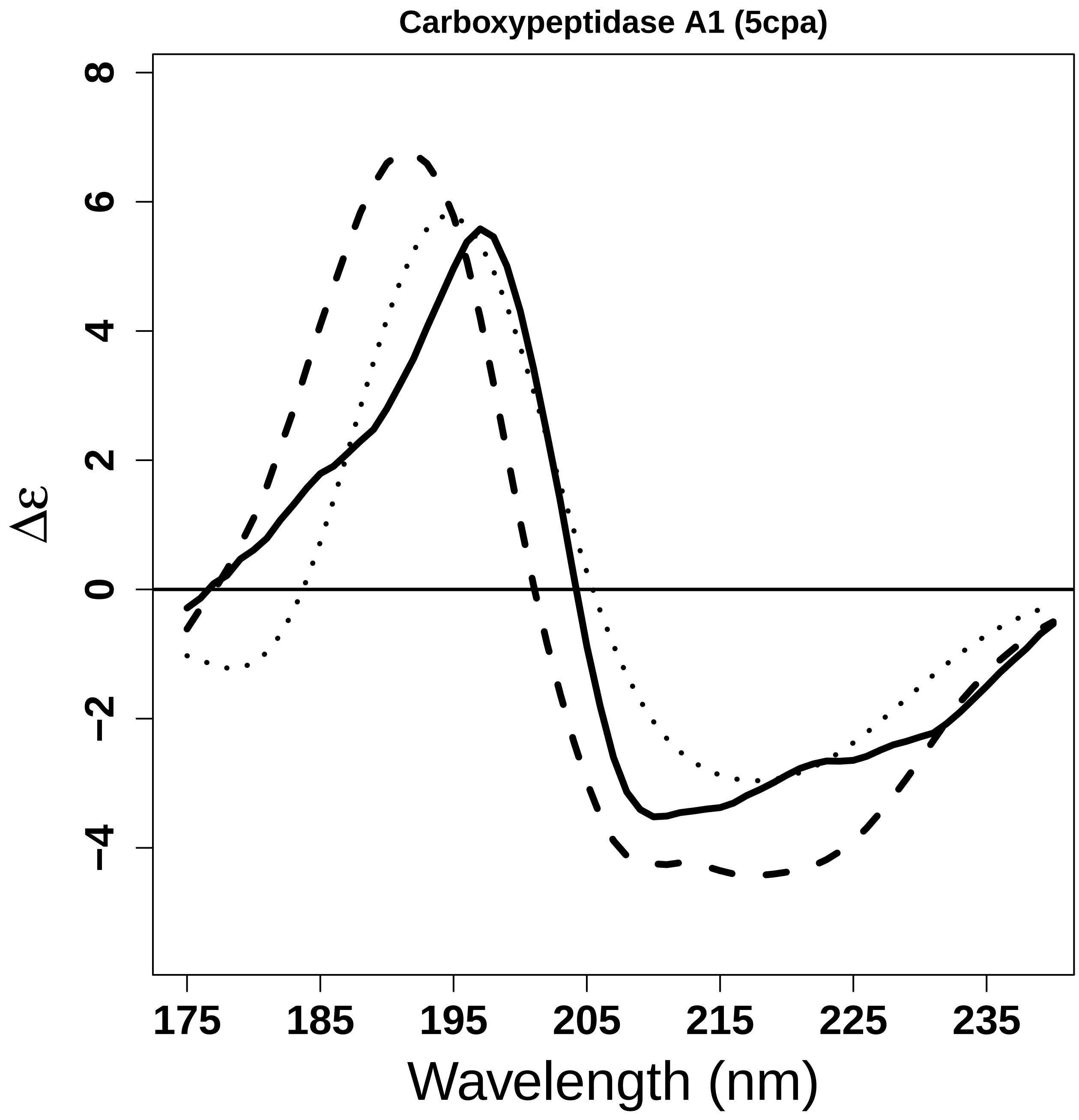

Carboxypeptidase A1 (5cpa)
Δε
8
6
4
2
0
−2
−4
175
185
195
205
215
225
235
Wavelength (nm)

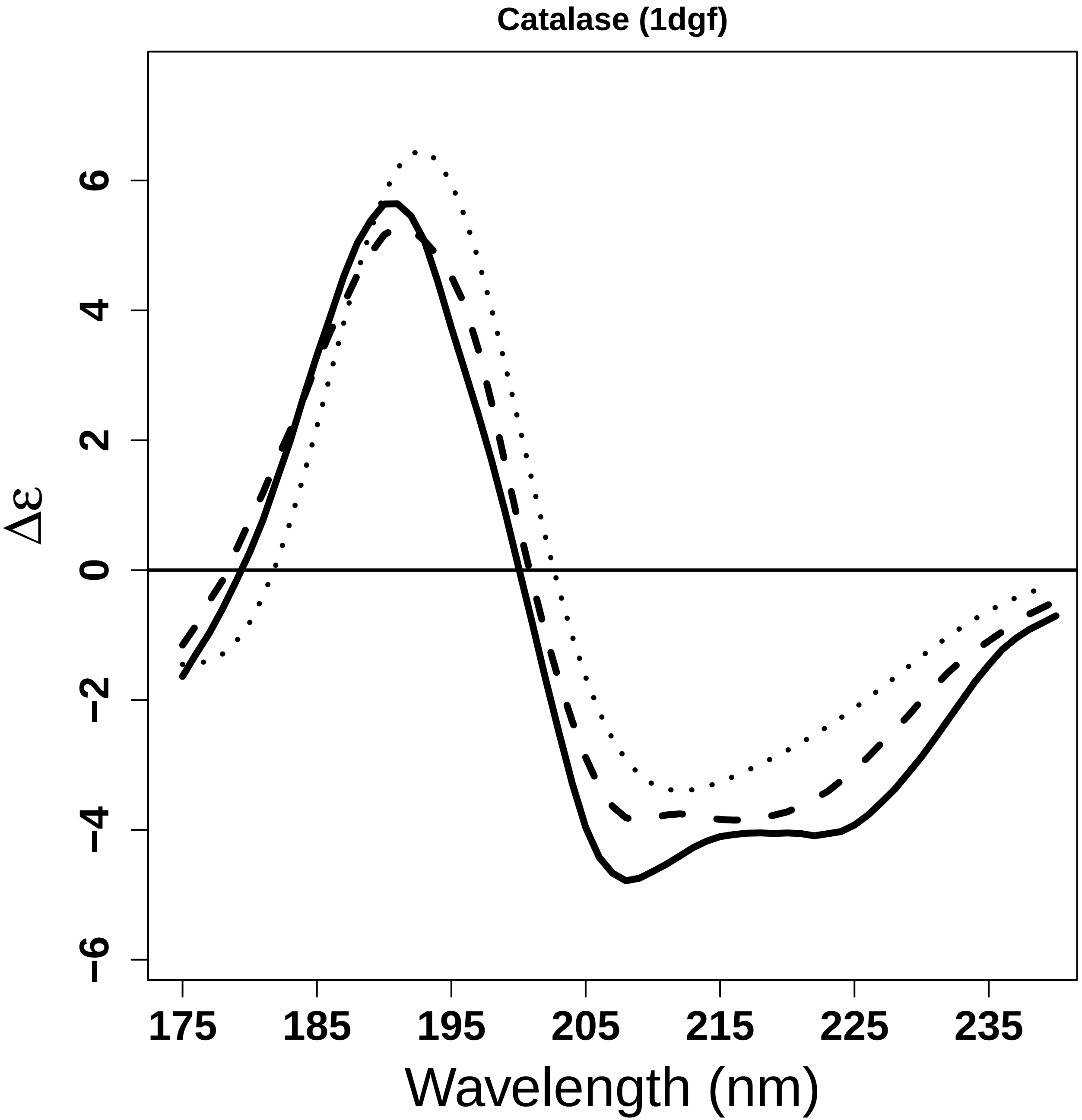
Catalase (1dgf)
Δε
6
4
2
0
−2
−4
−6
175
185
195
205
215
225
235
Wavelength (nm)

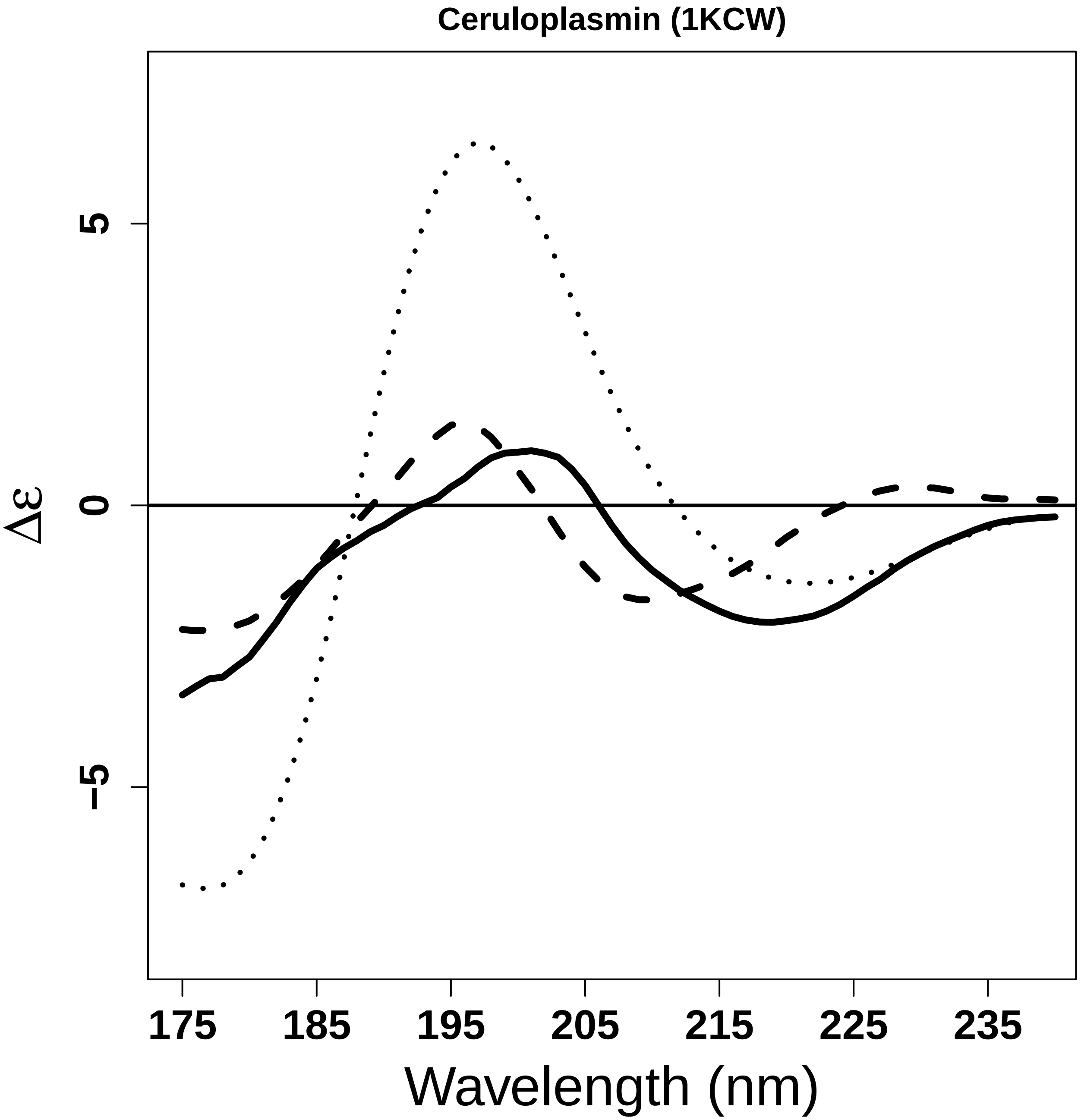

Ceruloplasmin (1KCW)
5
0
−5
Δε
175
185
195
205
215
225
235
Wavelength (nm)

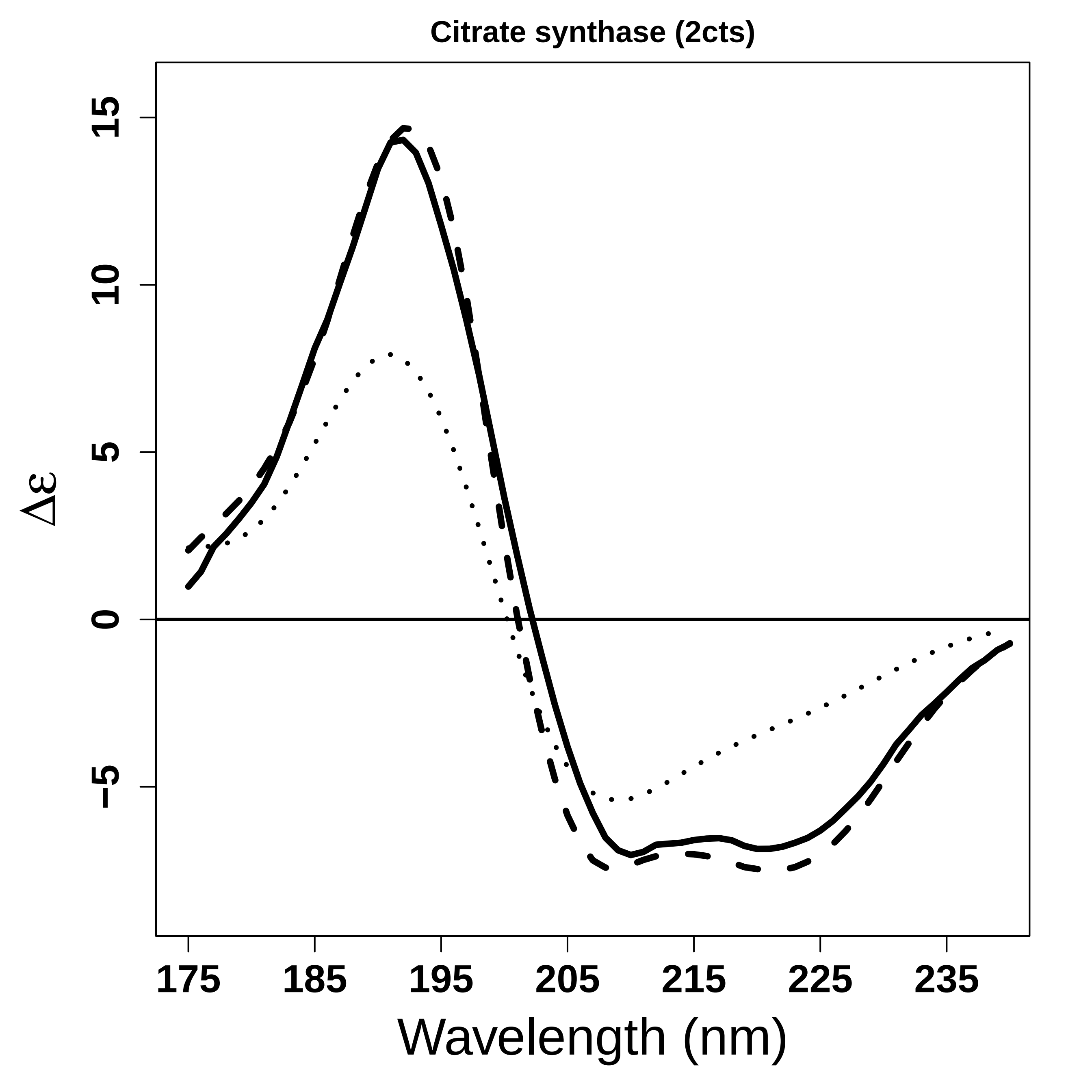

Citrate synthase (2cts)
15
10
5
0
−5
Δε
175
185
195
205
215
225
235
Wavelength (nm)

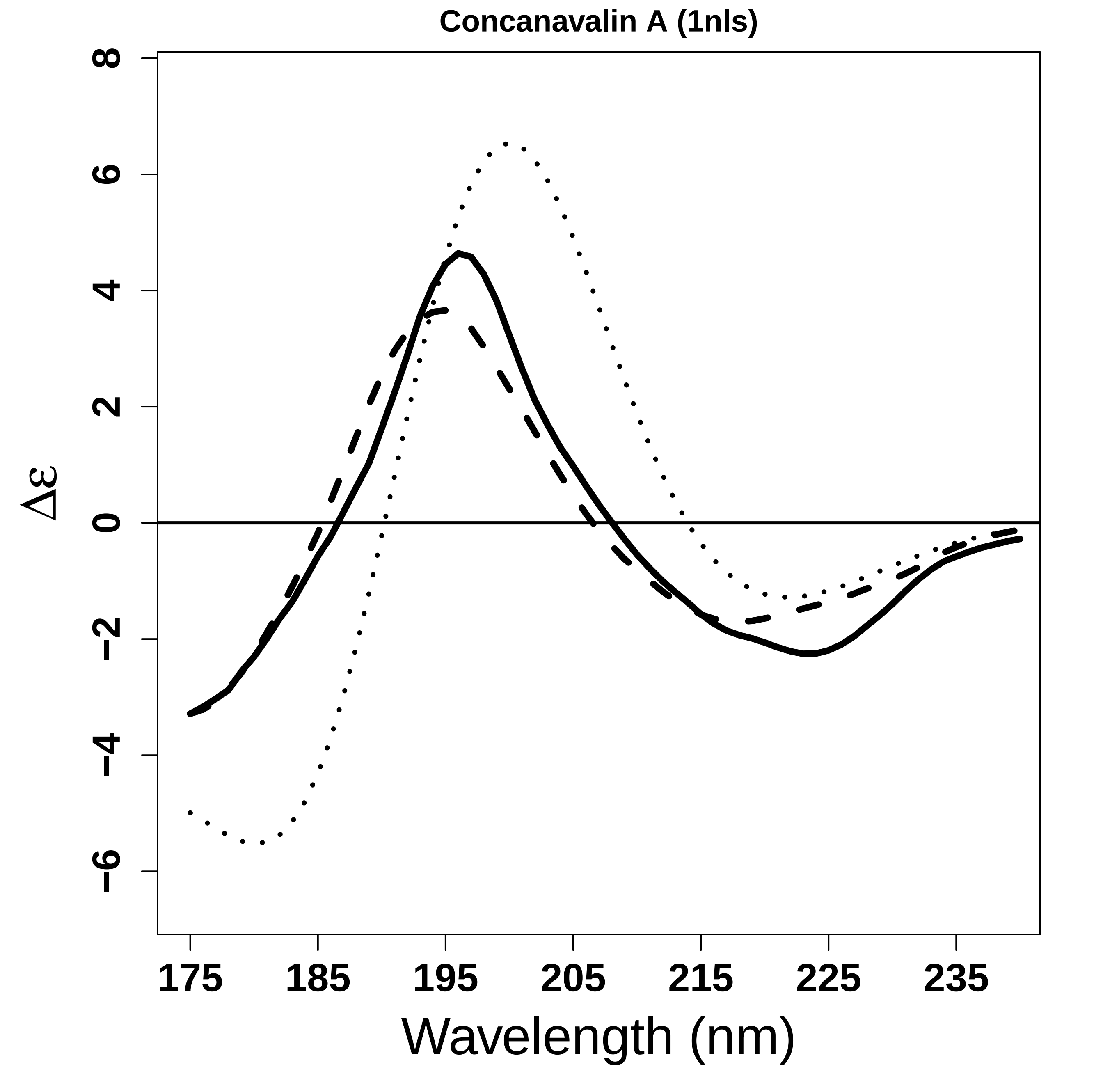

Concanavalin A (1nls)
Δε
8
6
4
2
0
−2
−4
−6
175
185
195
205
215
225
235
Wavelength (nm)

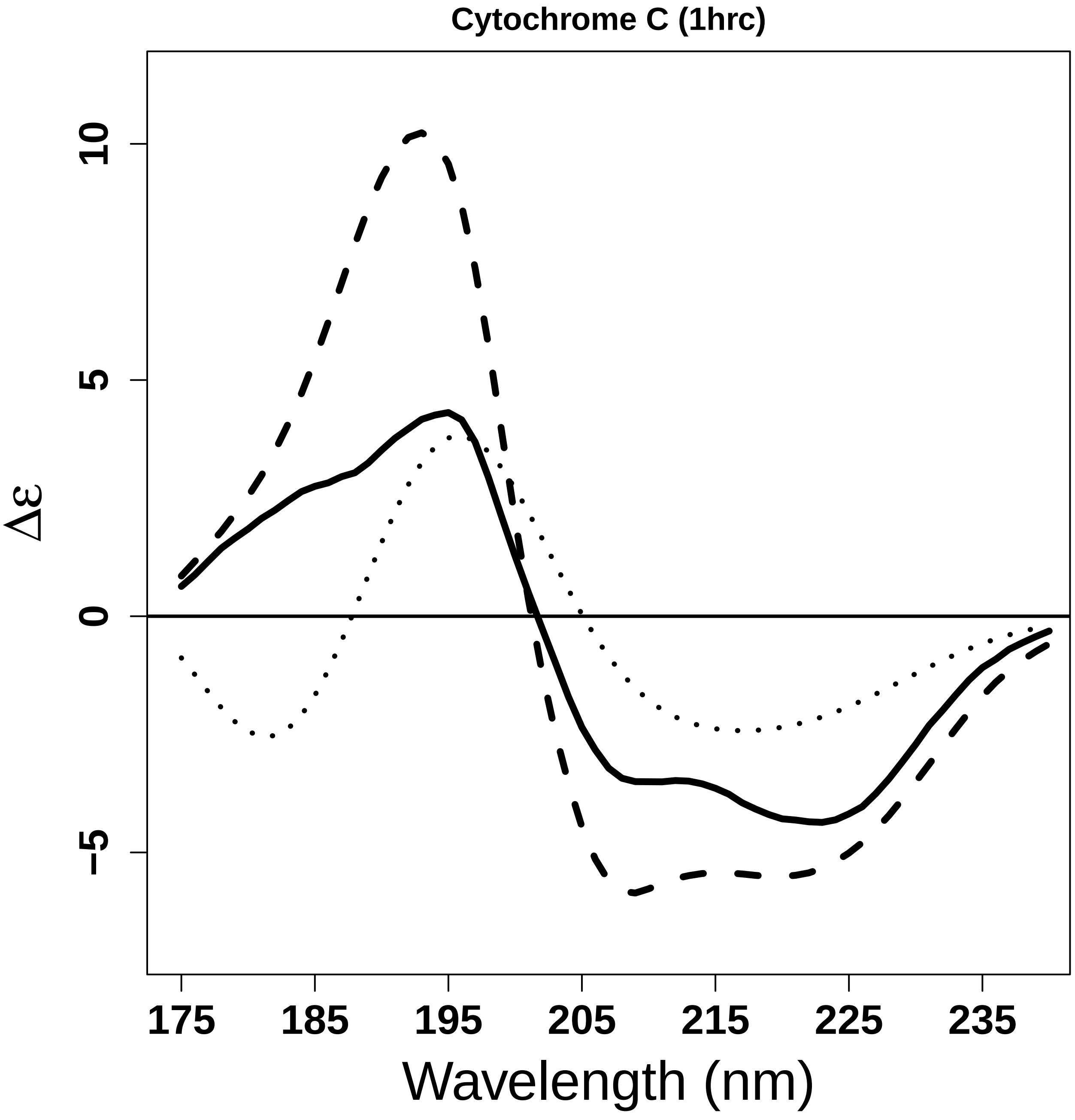

Cytochrome C (1hrc)
Δε
10
5
0
−5
175
185
195
205
215
225
235
Wavelength (nm)

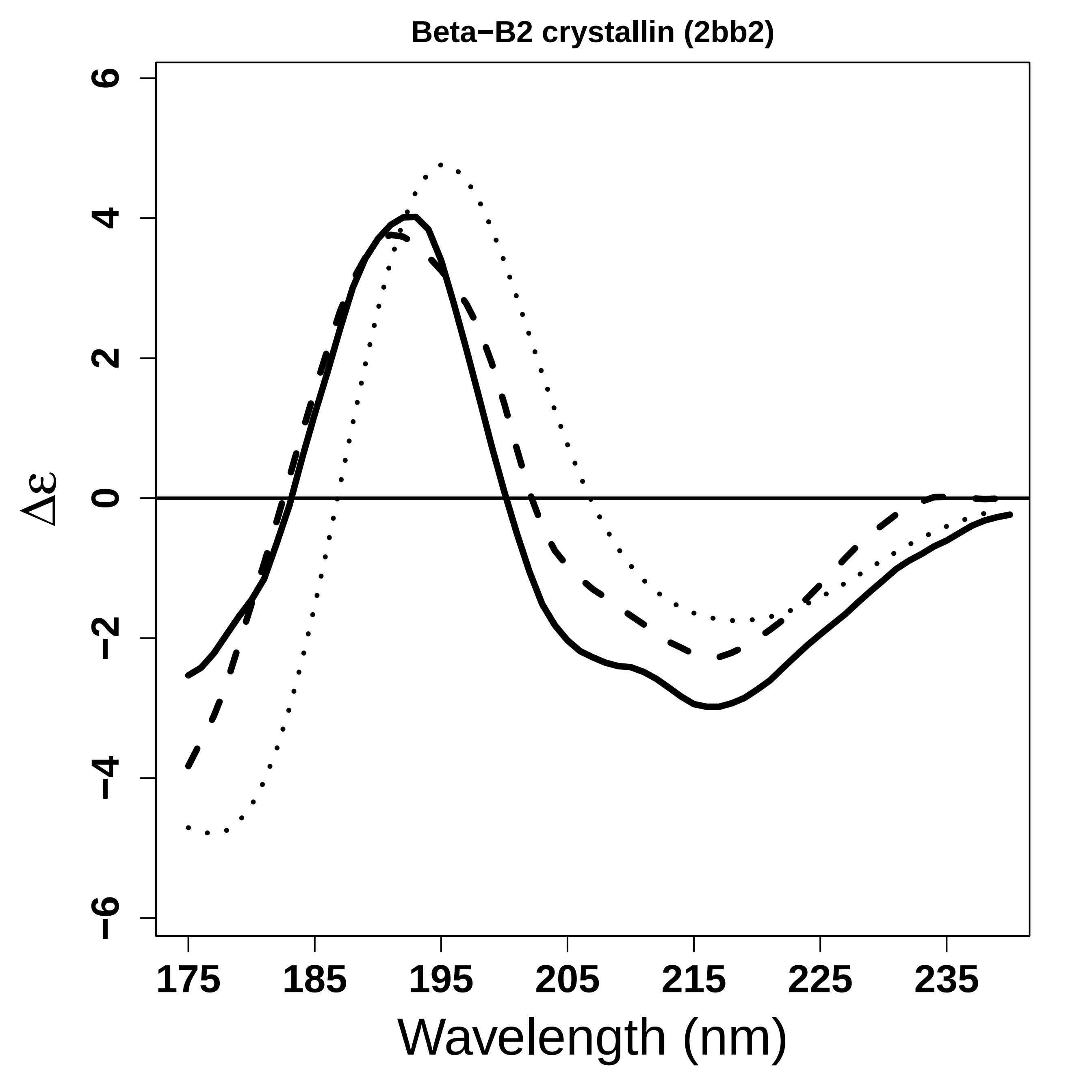
Beta−B2 crystallin (2bb2)
Δε
6
4
2
0
−2
−4
−6
175
185
195
205
215
225
235
Wavelength (nm)

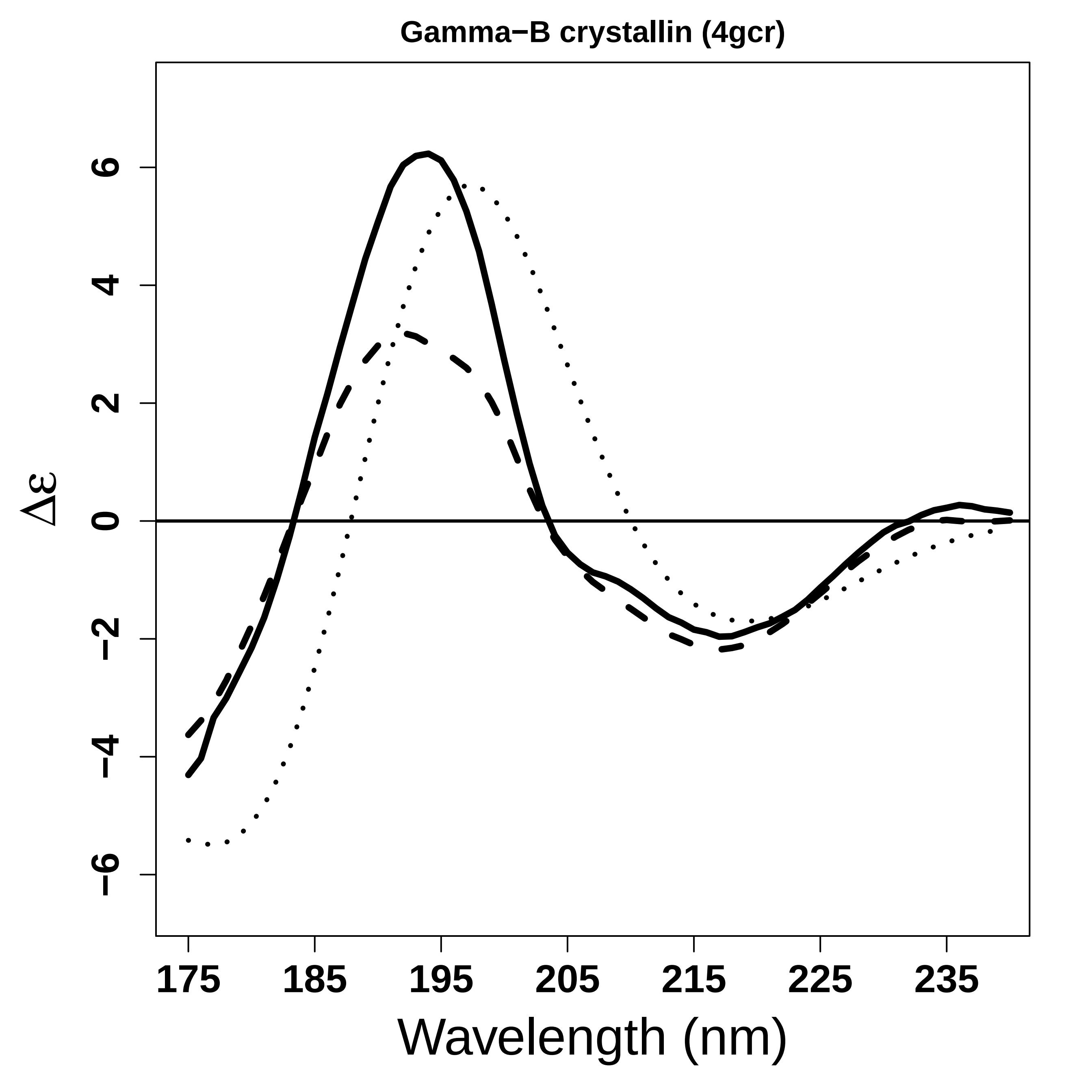
Gamma−B crystallin (4gcr)
Δε
6
4
2
0
−2
−4
−6
175
185
195
205
215
225
235
Wavelength (nm)

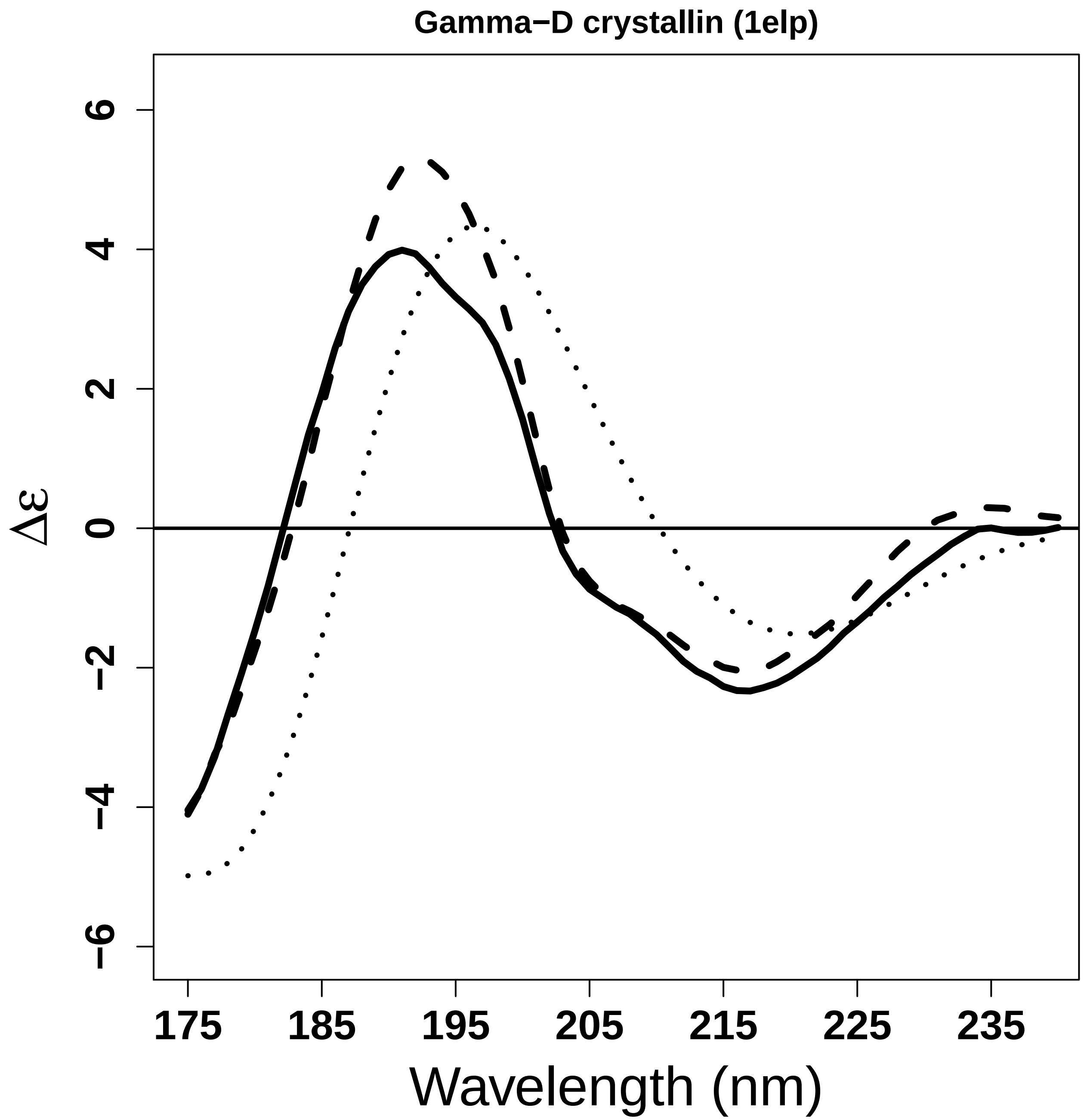
Gamma−D crystallin (1elp)
Δε
6
4
2
0
−2
−4
−6
175
185
195
205
215
225
235
Wavelength (nm)

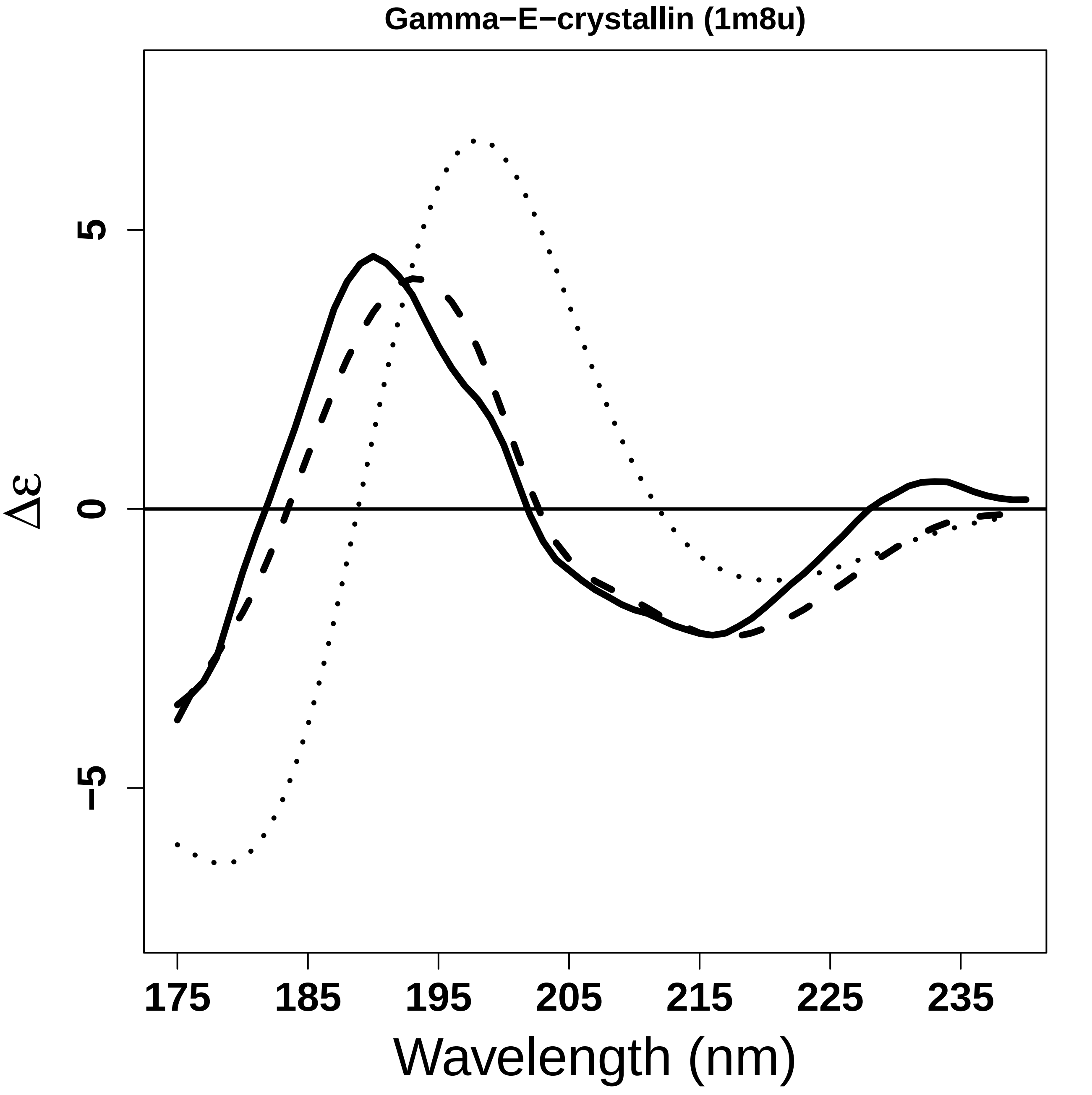

Gamma−E−crystallin (1m8u)
Δε
5
0
−5
175
185
195
205
215
225
235
Wavelength (nm)

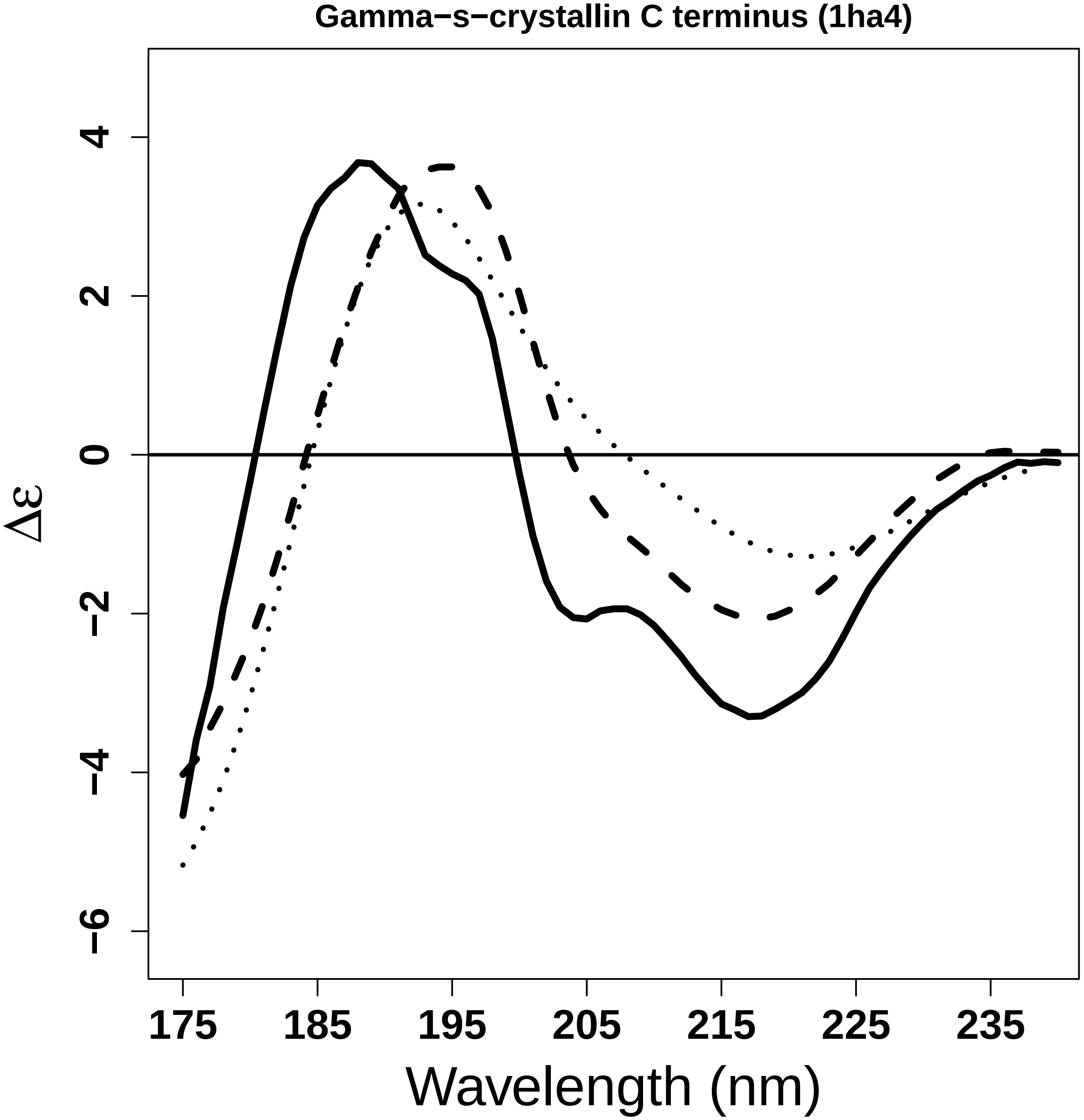

Gamma−s−crystallin C terminus (1ha4)
4
2
0
−2
−4
−6
Δε
175
185
195
205
215
225
235
Wavelength (nm)

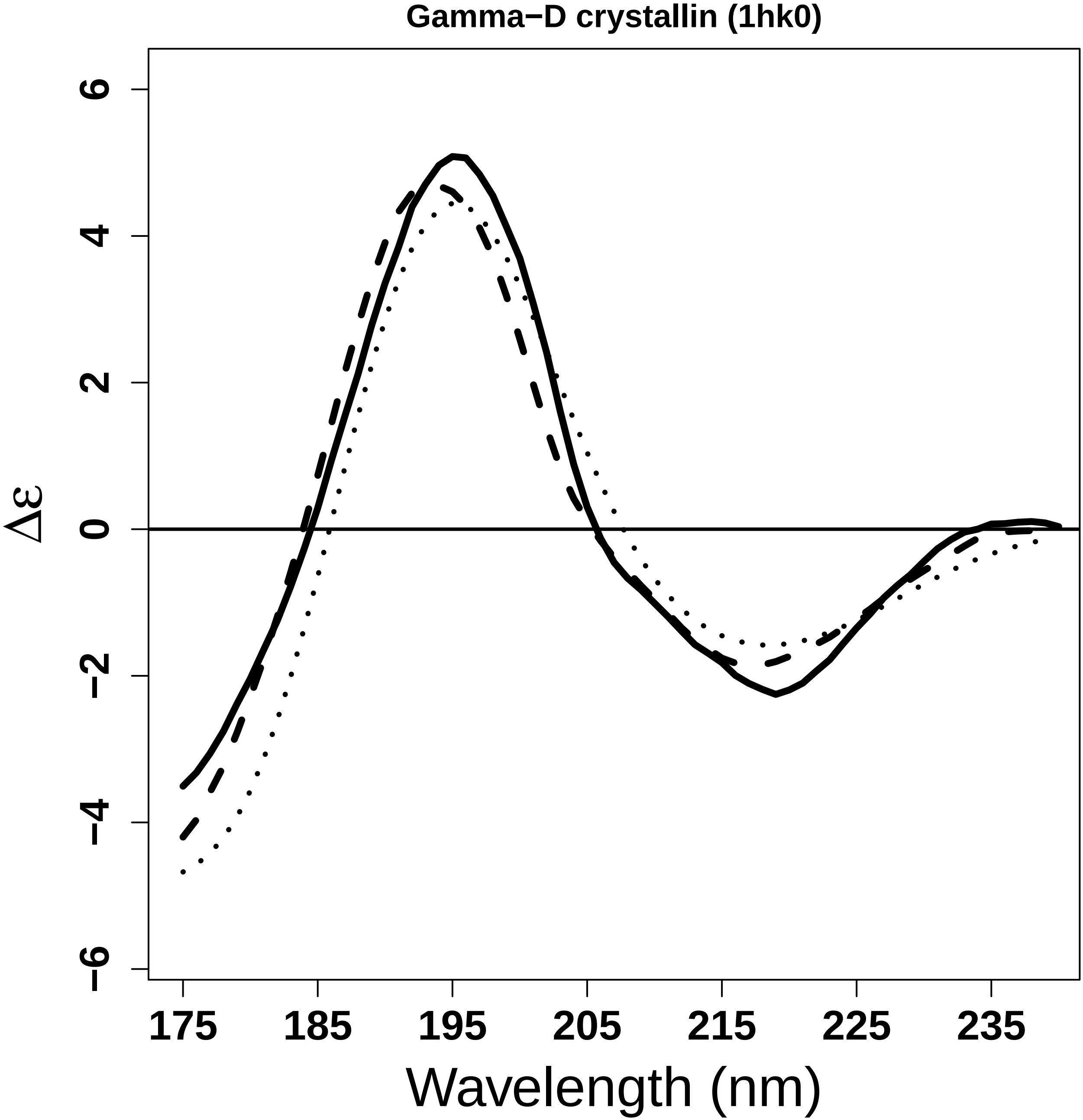

Gamma−D crystallin (1hk0)
Δε
6
4
2
0
−2
−4
−6
175
185
195
205
215
225
235
Wavelength (nm)

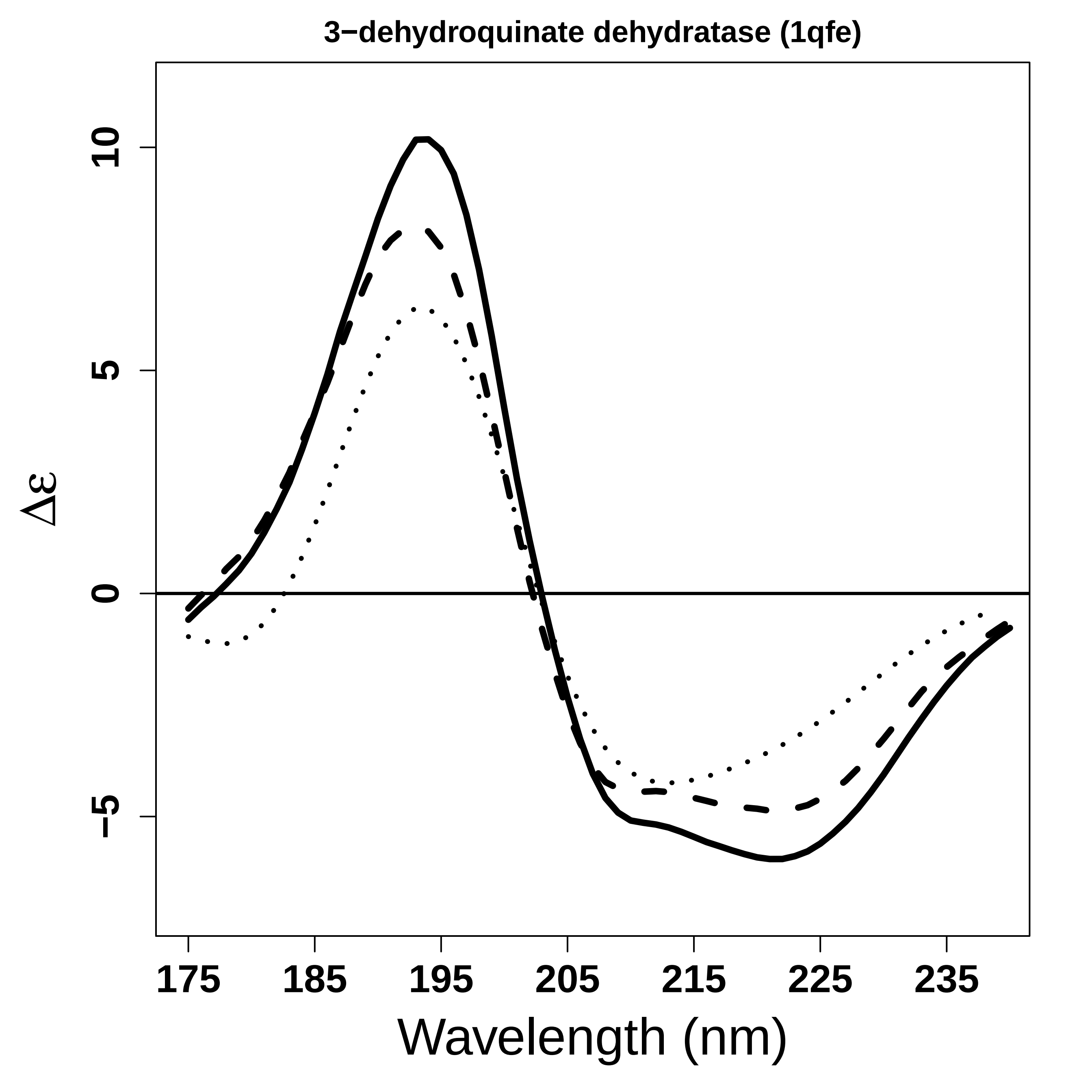
3−dehydroquinate dehydratase (1qfe)
Δε
10
5
0
−5
175
185
195
205
215
225
235
Wavelength (nm)

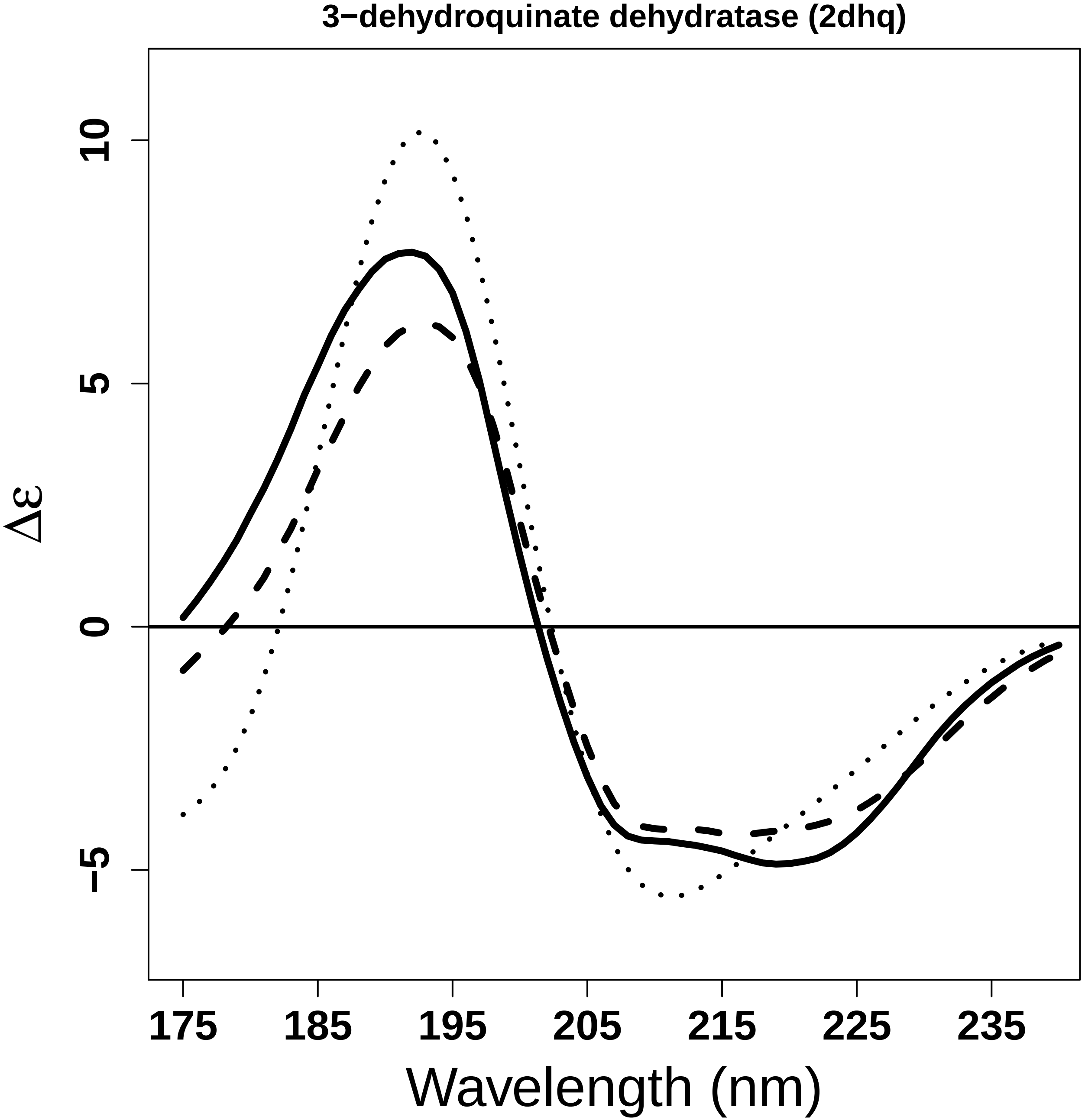

3−dehydroquinate dehydratase (2dhq)
Δε
10
5
0
−5
175
185
195
205
215
225
235
Wavelength (nm)

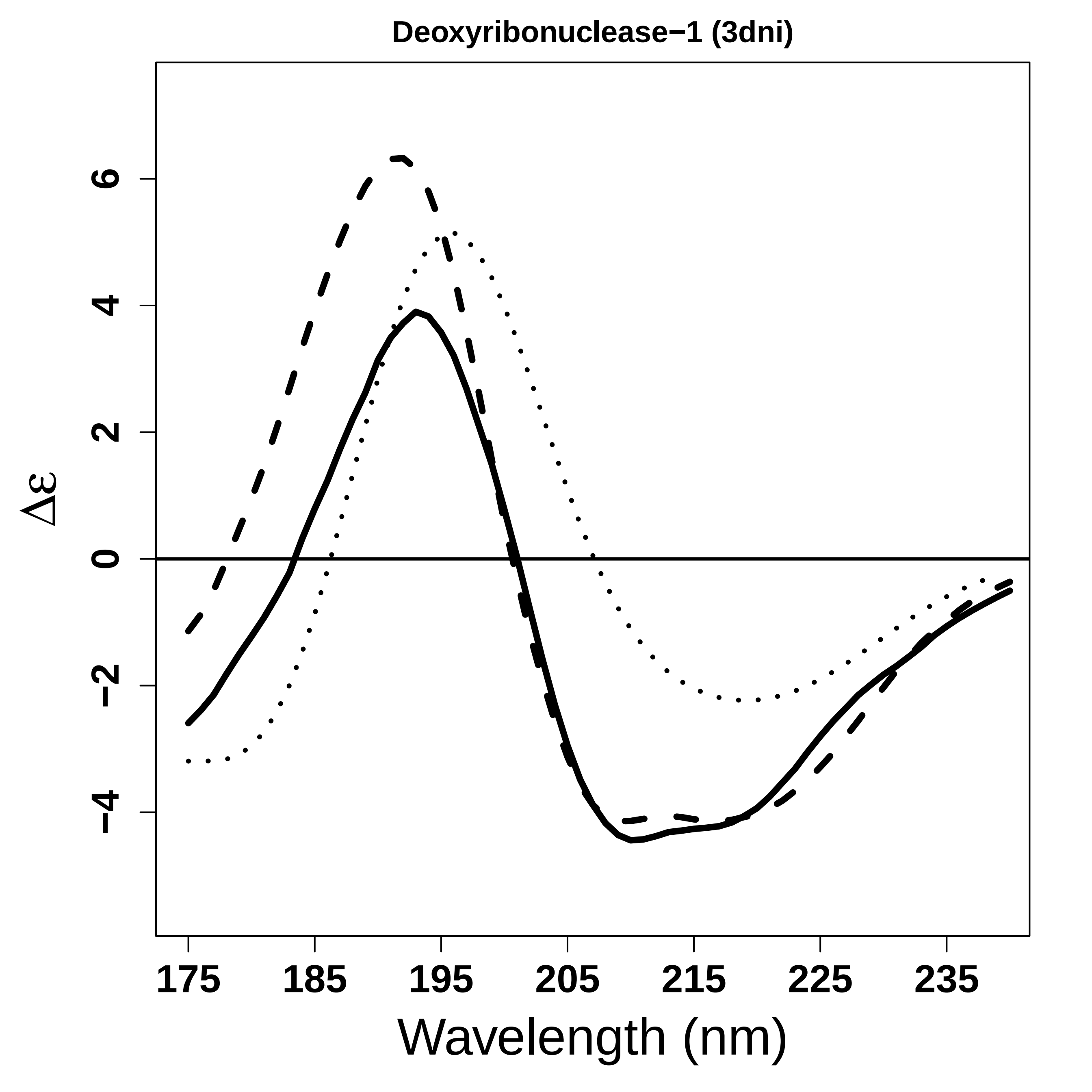

Deoxyribonuclease−1 (3dni)
6
4
2
0
−2
−4
Δε
175
185
195
205
215
225
235
Wavelength (nm)

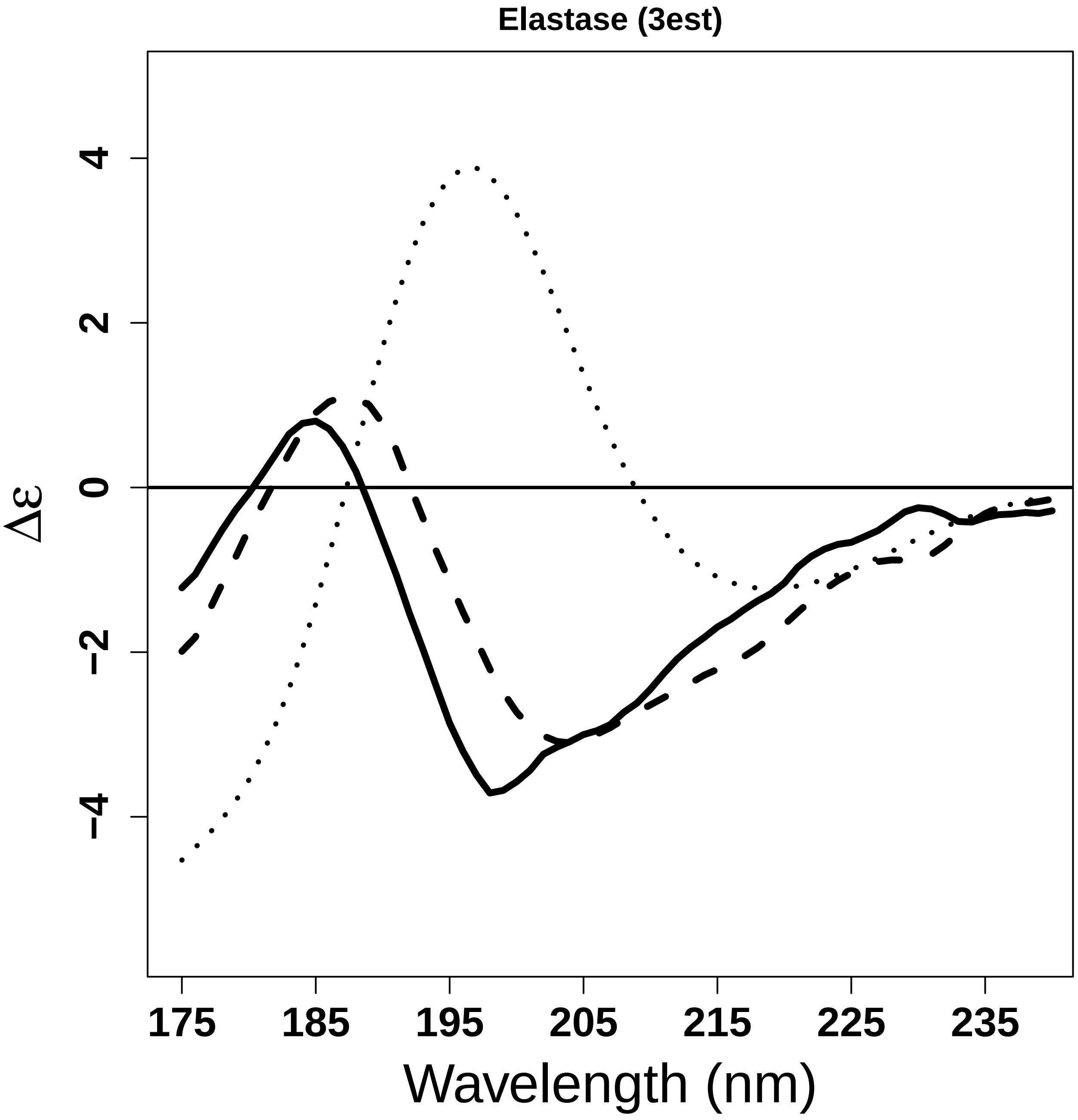

Elastase (3est)
4
2
0
−2
−4
Δε
175
185
195
205
215
225
235
Wavelength (nm)

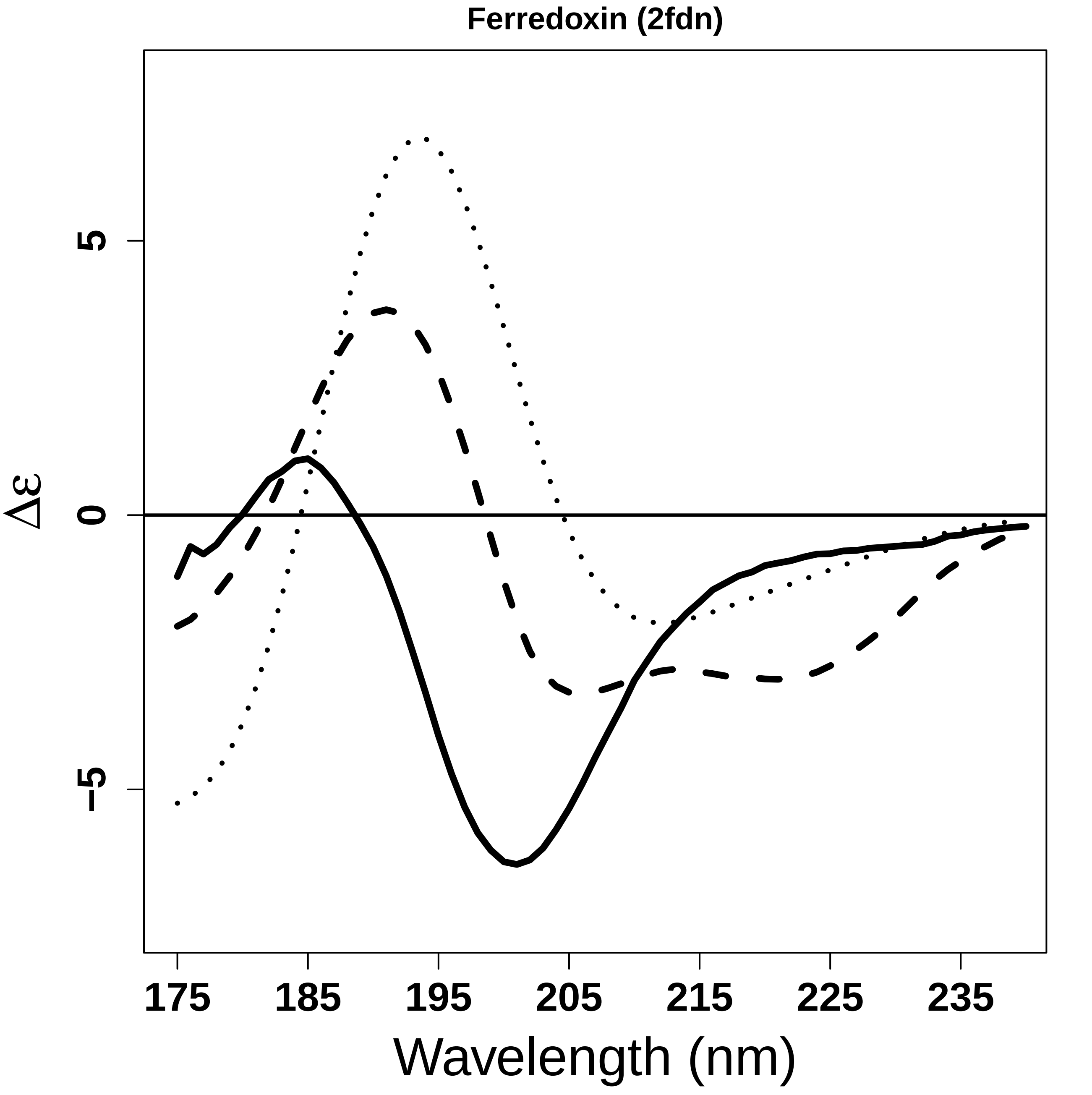

Ferredoxin (2fdn)
5
0
−5
Δε
175
185
195
205
215
225
235
Wavelength (nm)

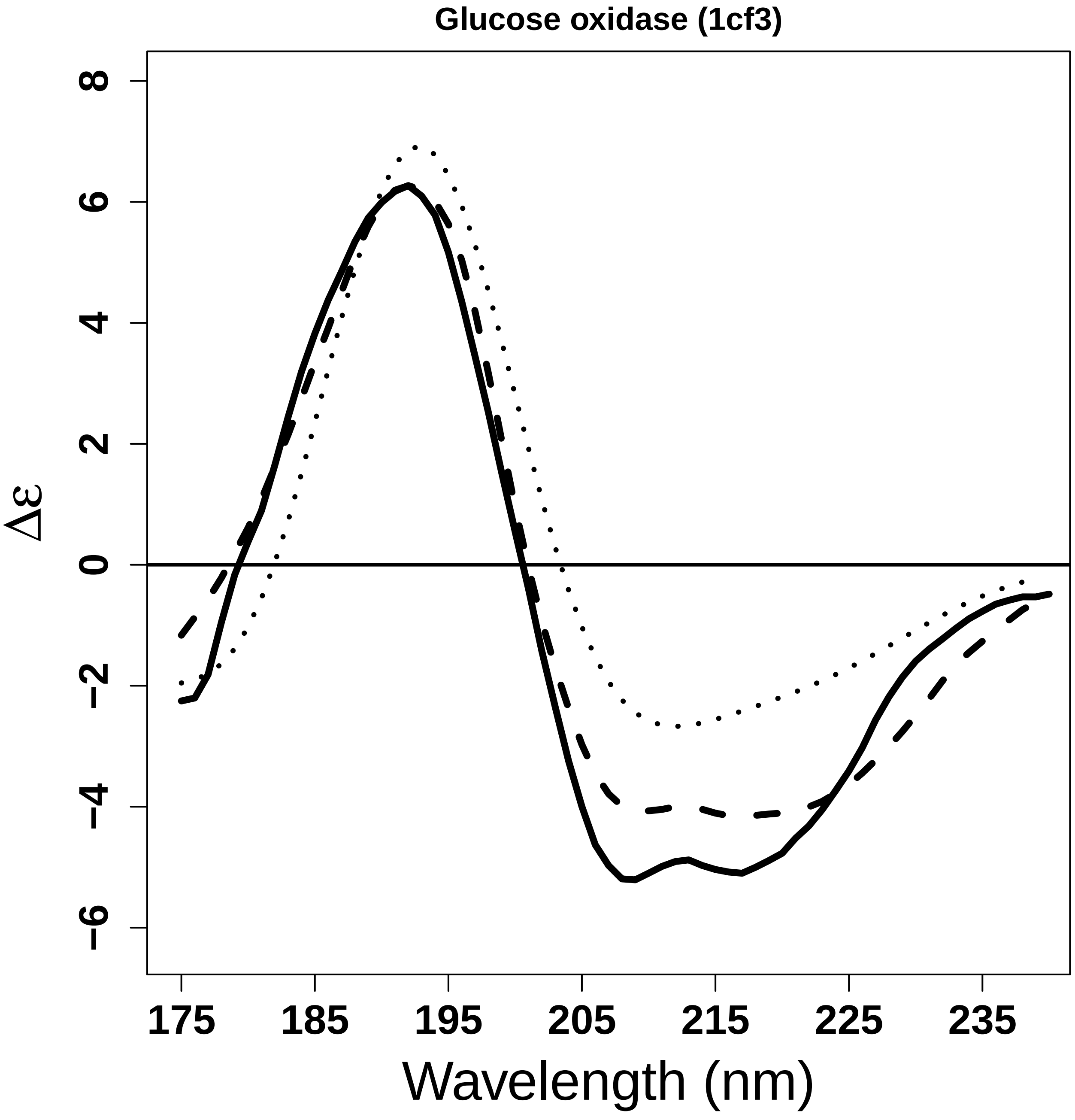

Glucose oxidase (1cf3)
Δε
8
6
4
2
0
−2
−4
−6
175
185
195
205
215
225
235
Wavelength (nm)

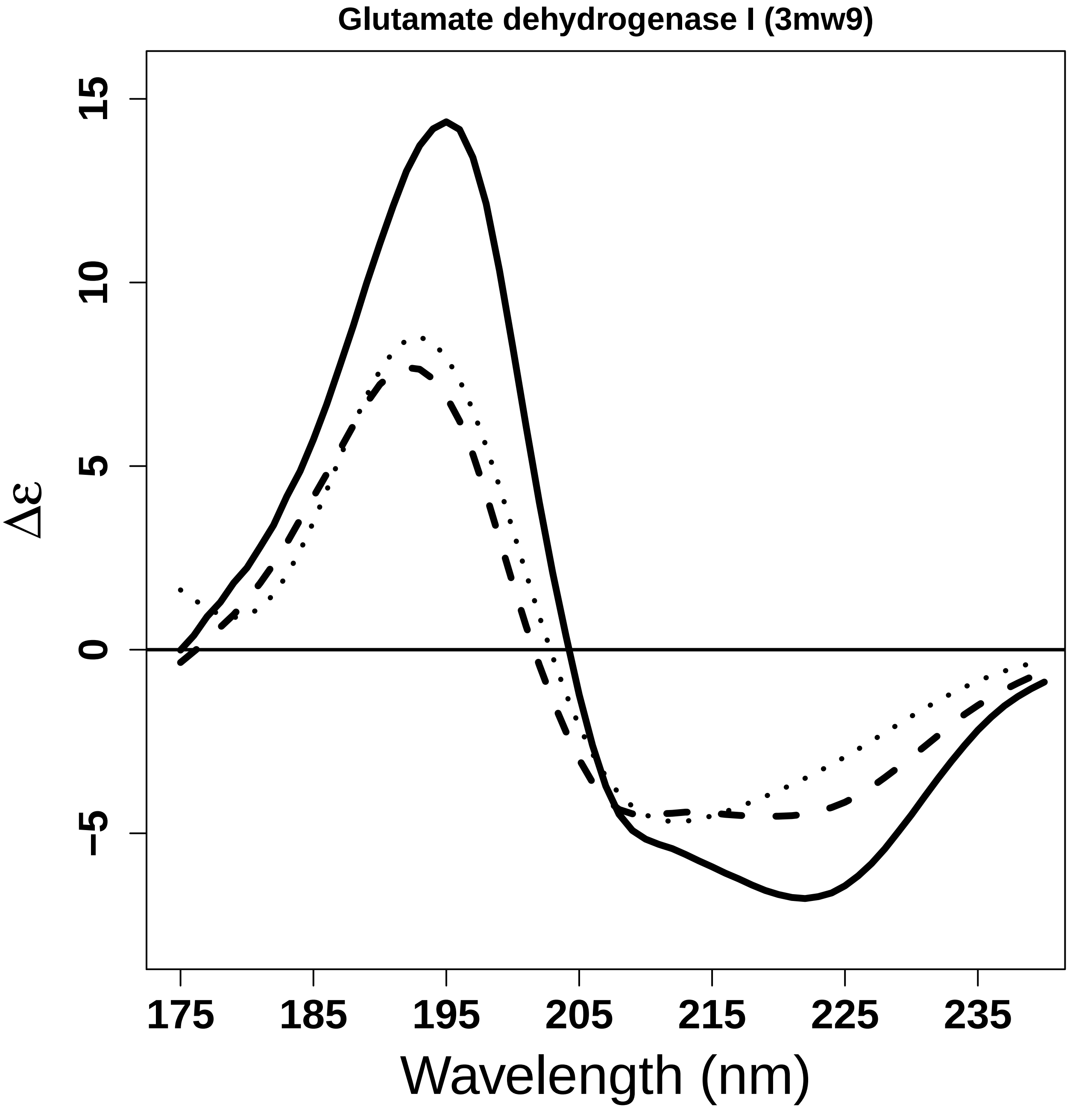

Glutamate dehydrogenase I (3mw9)
Δε
15
10
5
0
−5
175
185
195
205
215
225
235
Wavelength (nm)

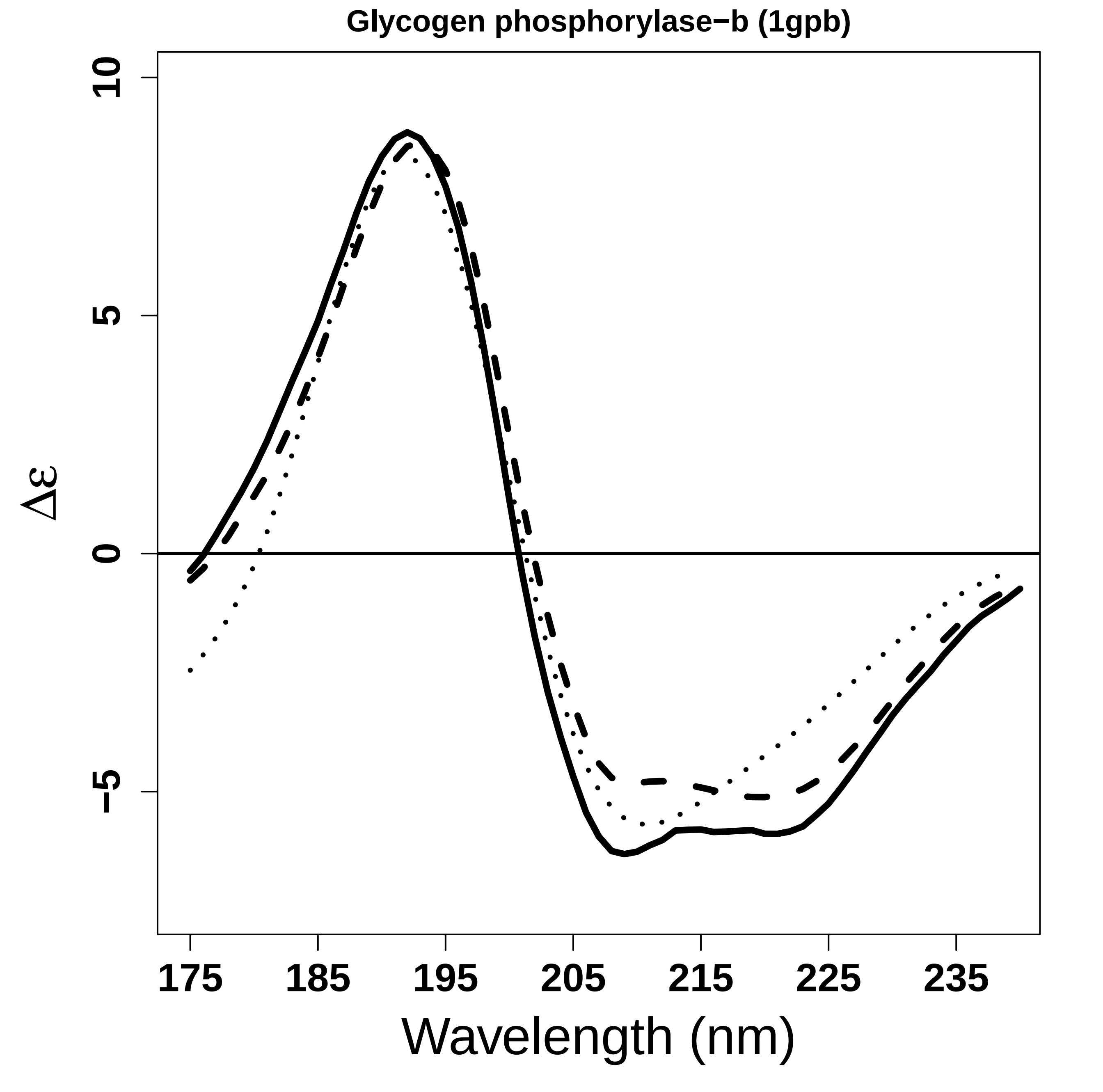

Glycogen phosphorylase−b (1gpb)
10
5
0
−5
Δε
175
185
195
205
215
225
235
Wavelength (nm)

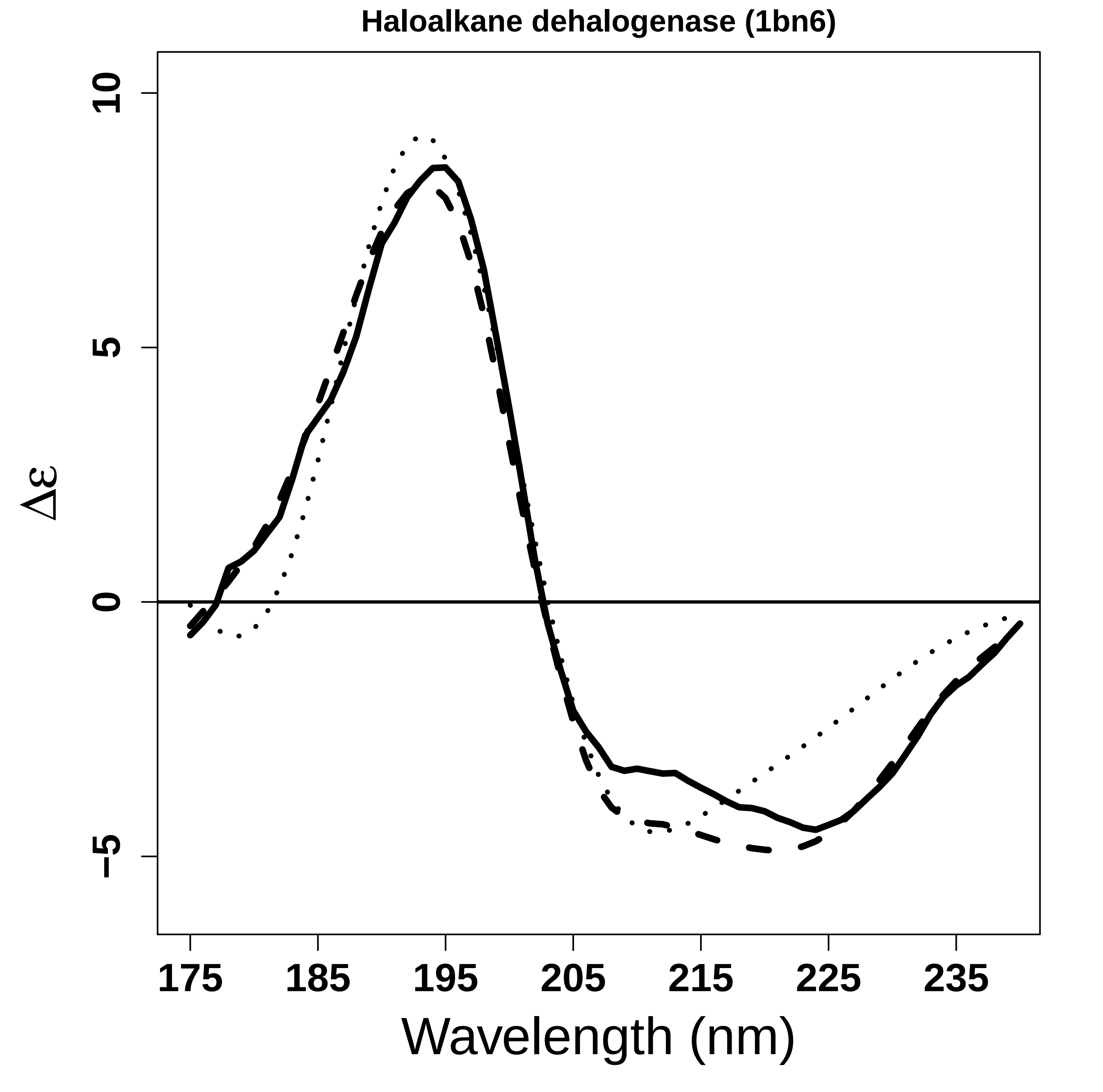

Haloalkane dehalogenase (1bn6)
10
5
0
−5
Δε
175
185
195
205
215
225
235
Wavelength (nm)

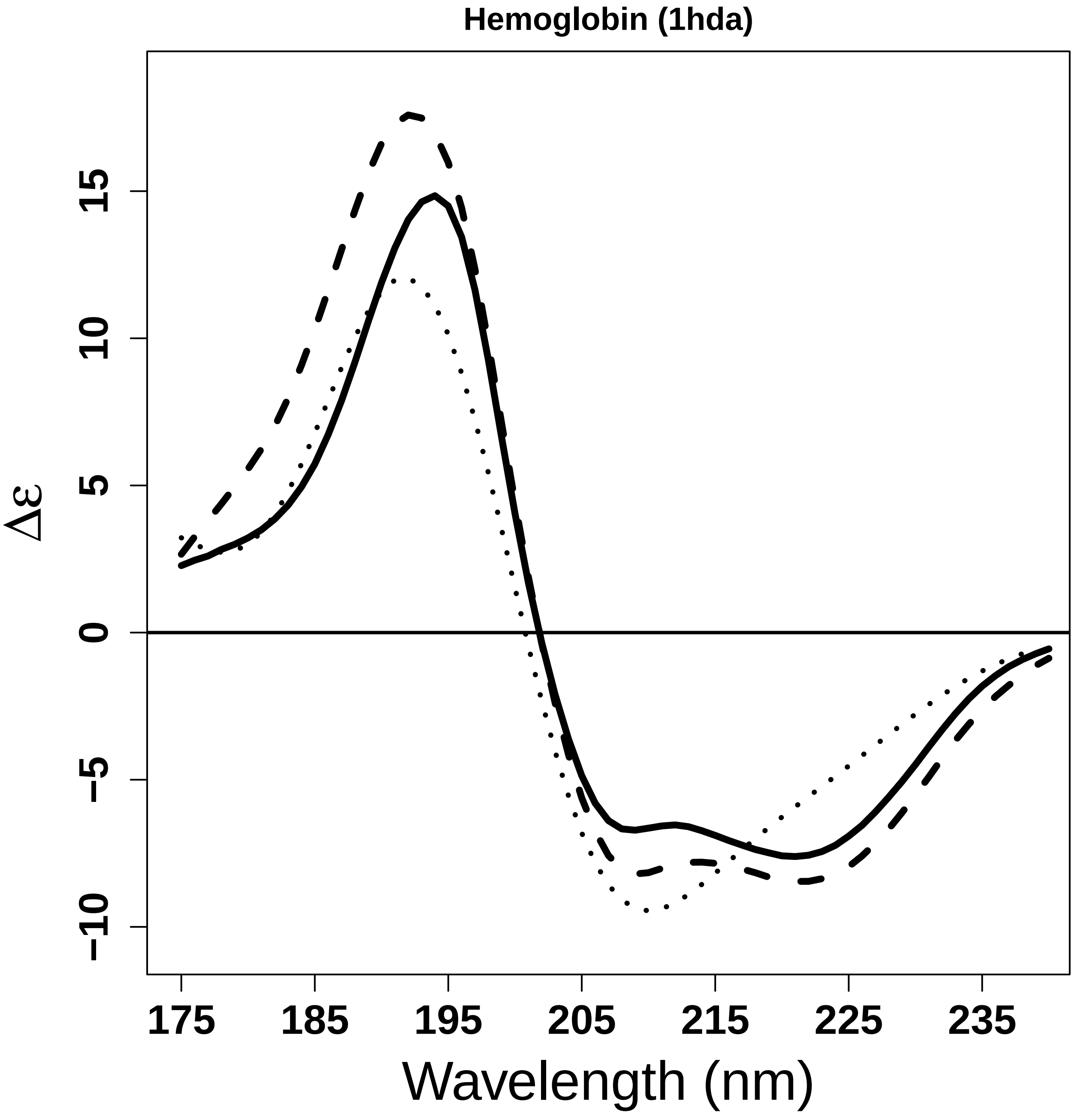

Hemoglobin (1hda)
15
10
5
0
−5
−10
Δε
175
185
195
205
215
225
235
Wavelength (nm)

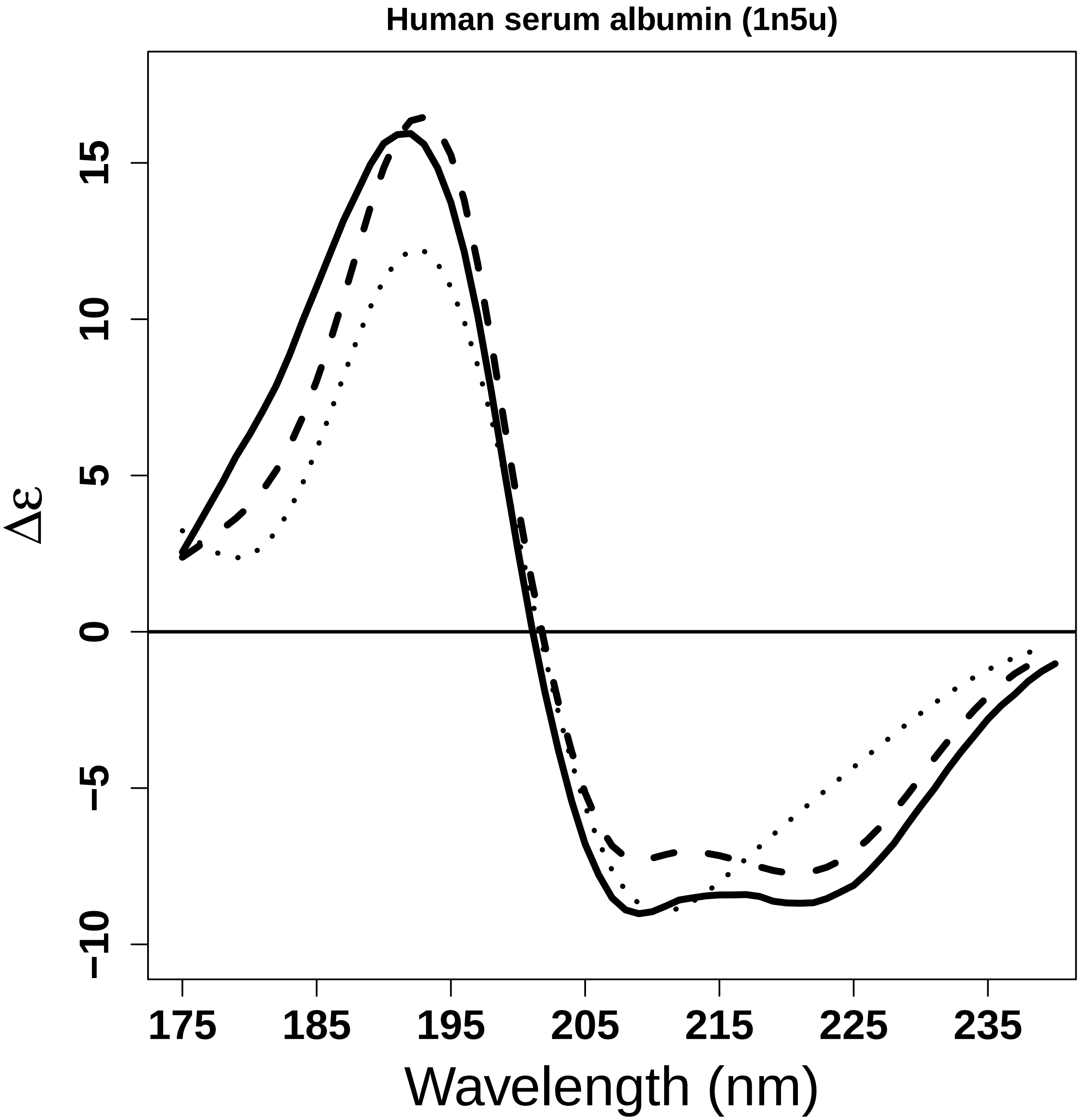

Human serum albumin (1n5u)
Δε
15
10
5
0
−5
−10
175
185
195
205
215
225
235
Wavelength (nm)

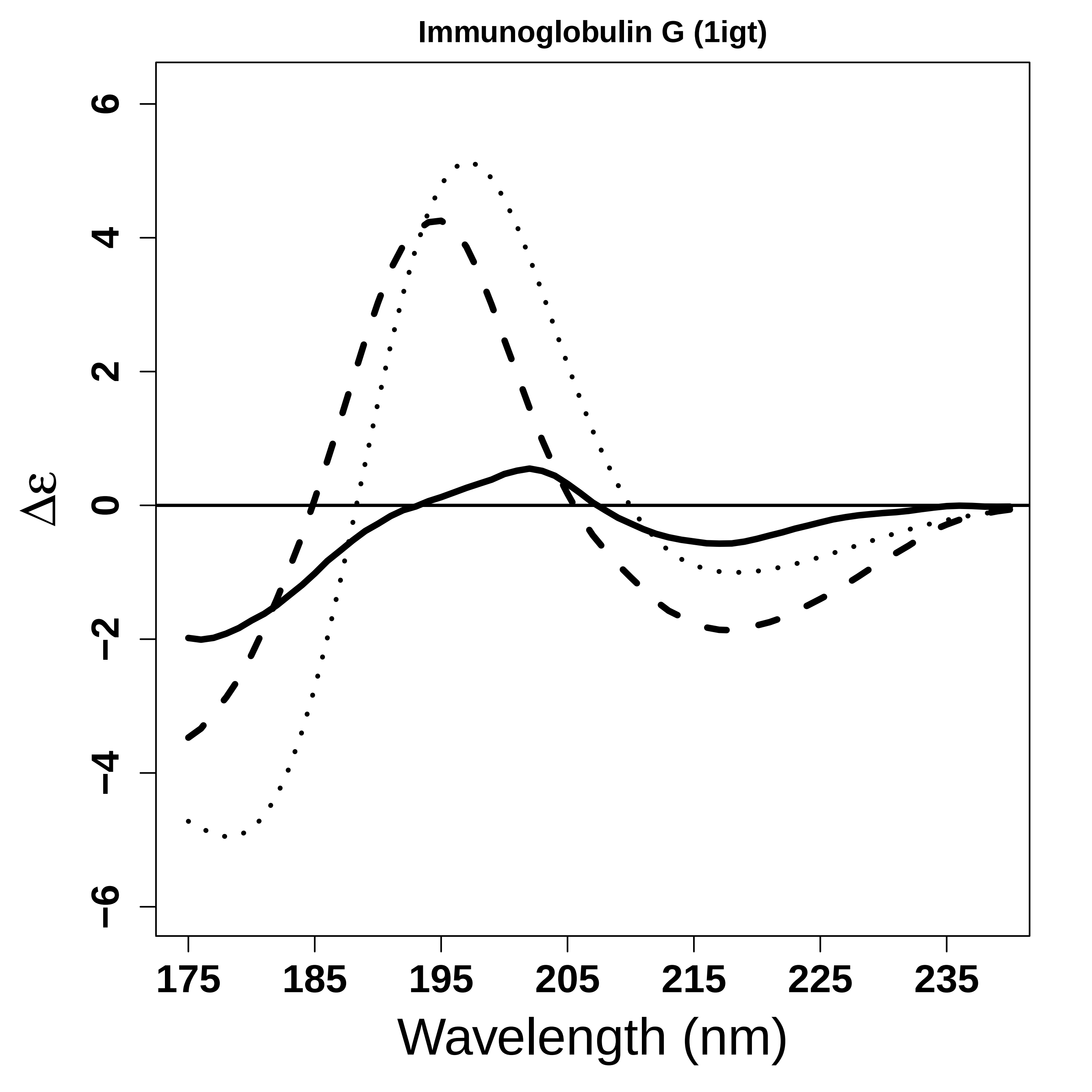

Immunoglobulin G (1igt)
Δε
6
4
2
0
−2
−4
−6
175
185
195
205
215
225
235
Wavelength (nm)

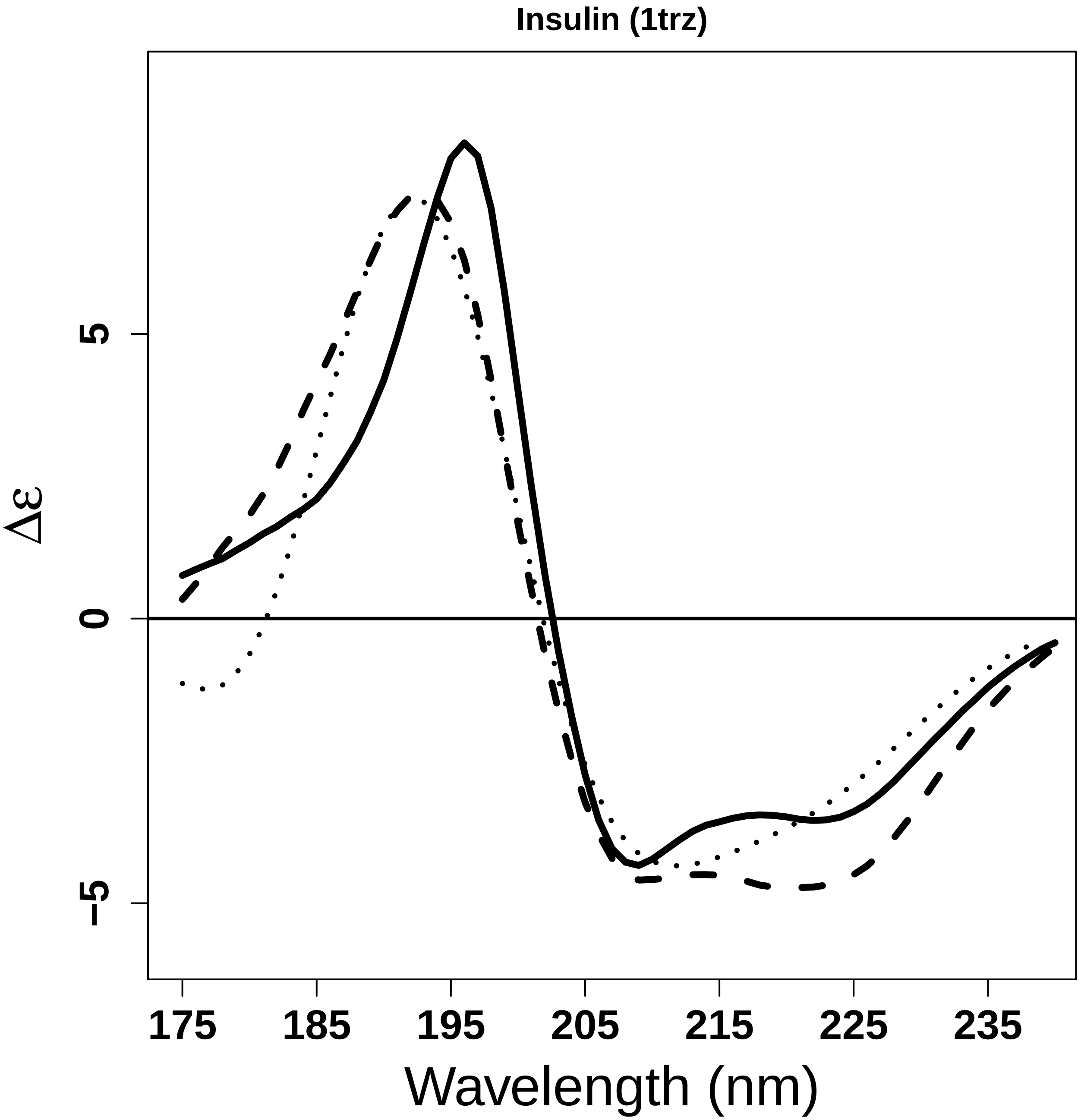

Insulin (1trz)
Δε
5
0
−5
175
185
195
205
215
225
235
Wavelength (nm)

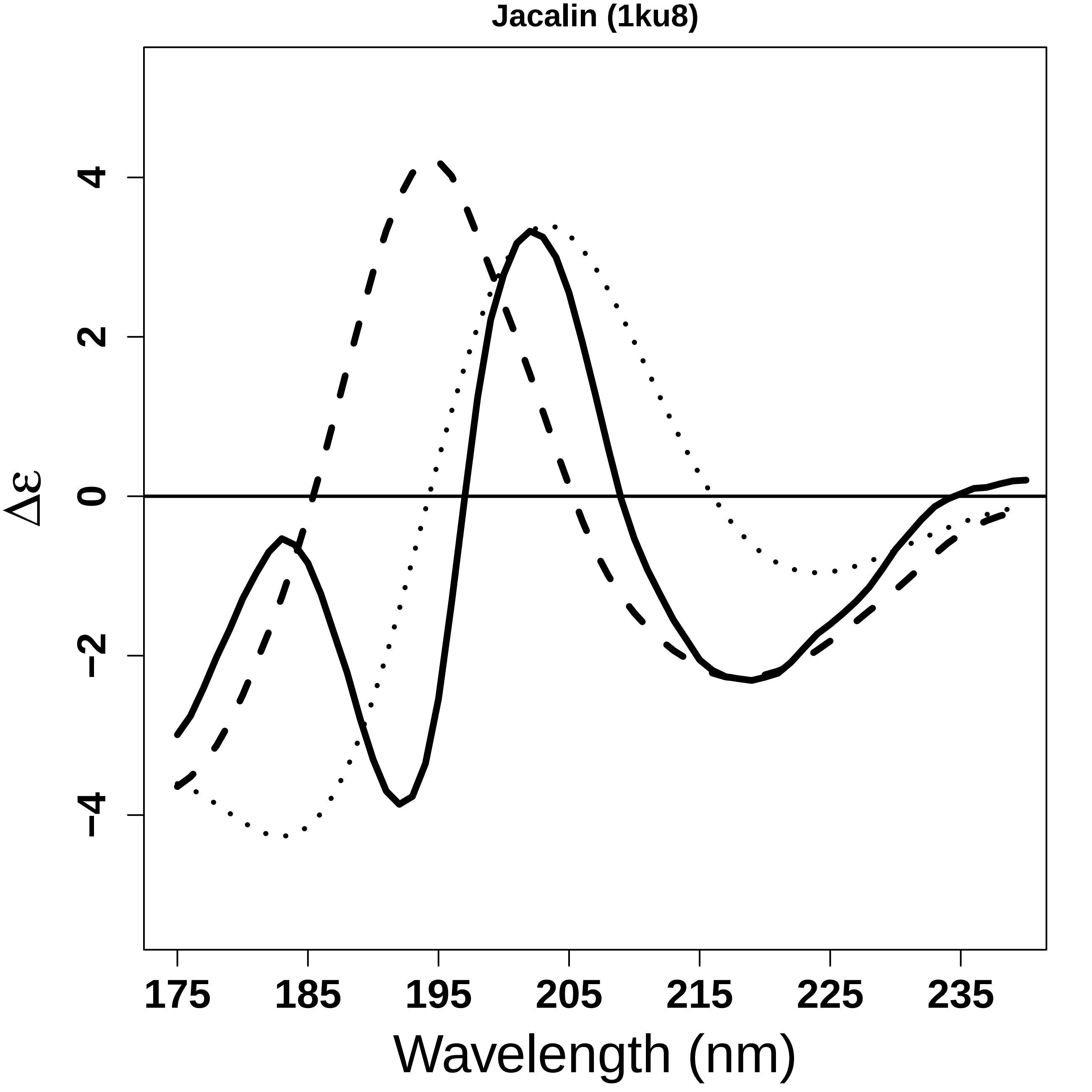

Jacalin (1ku8)
Δε
4
2
0
−2
−4
175
185
195
205
215
225
235
Wavelength (nm)

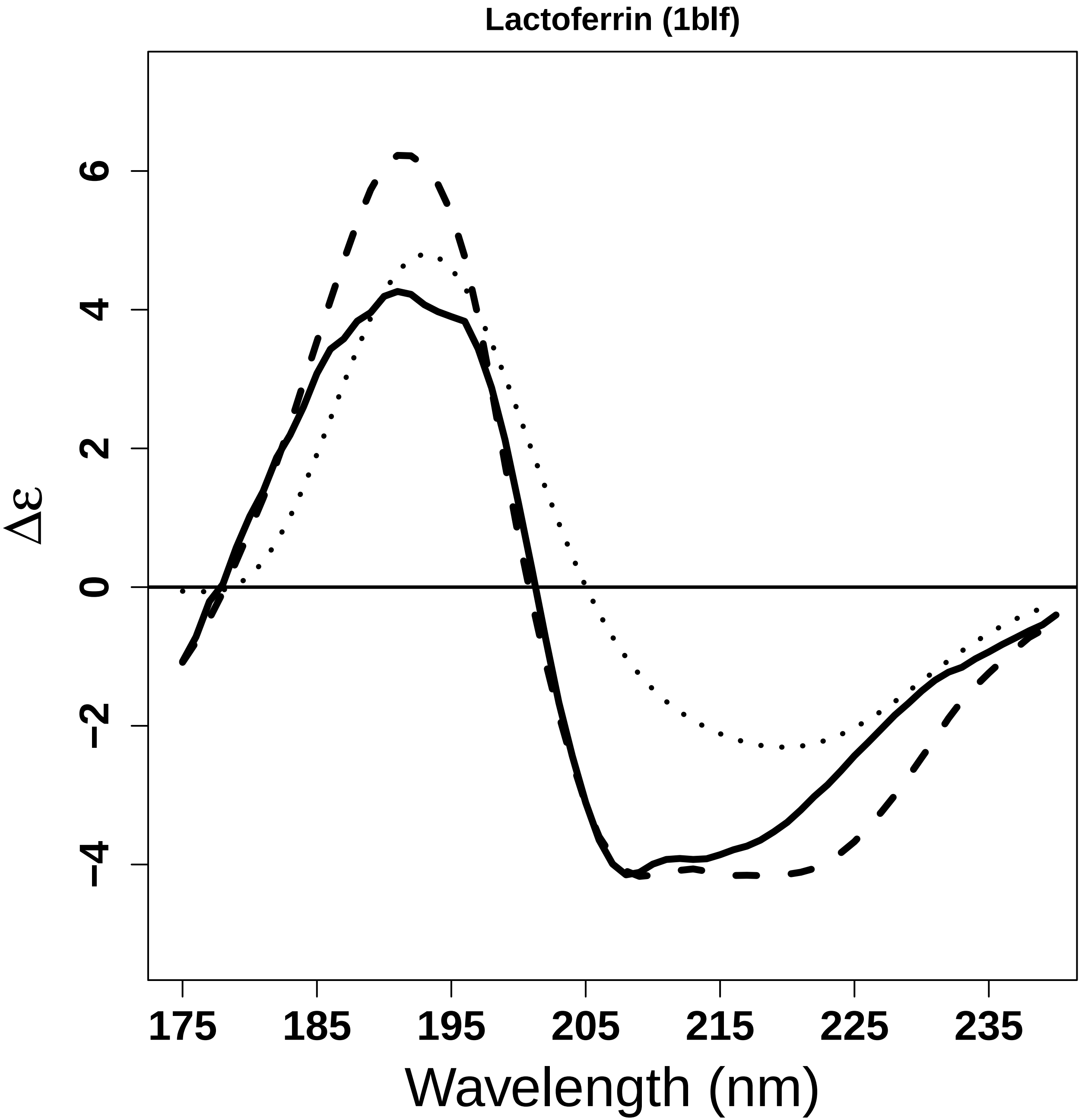
Lactoferrin (1blf)
6
4
2
0
−2
−4
Δε
175
185
195
205
215
225
235
Wavelength (nm)

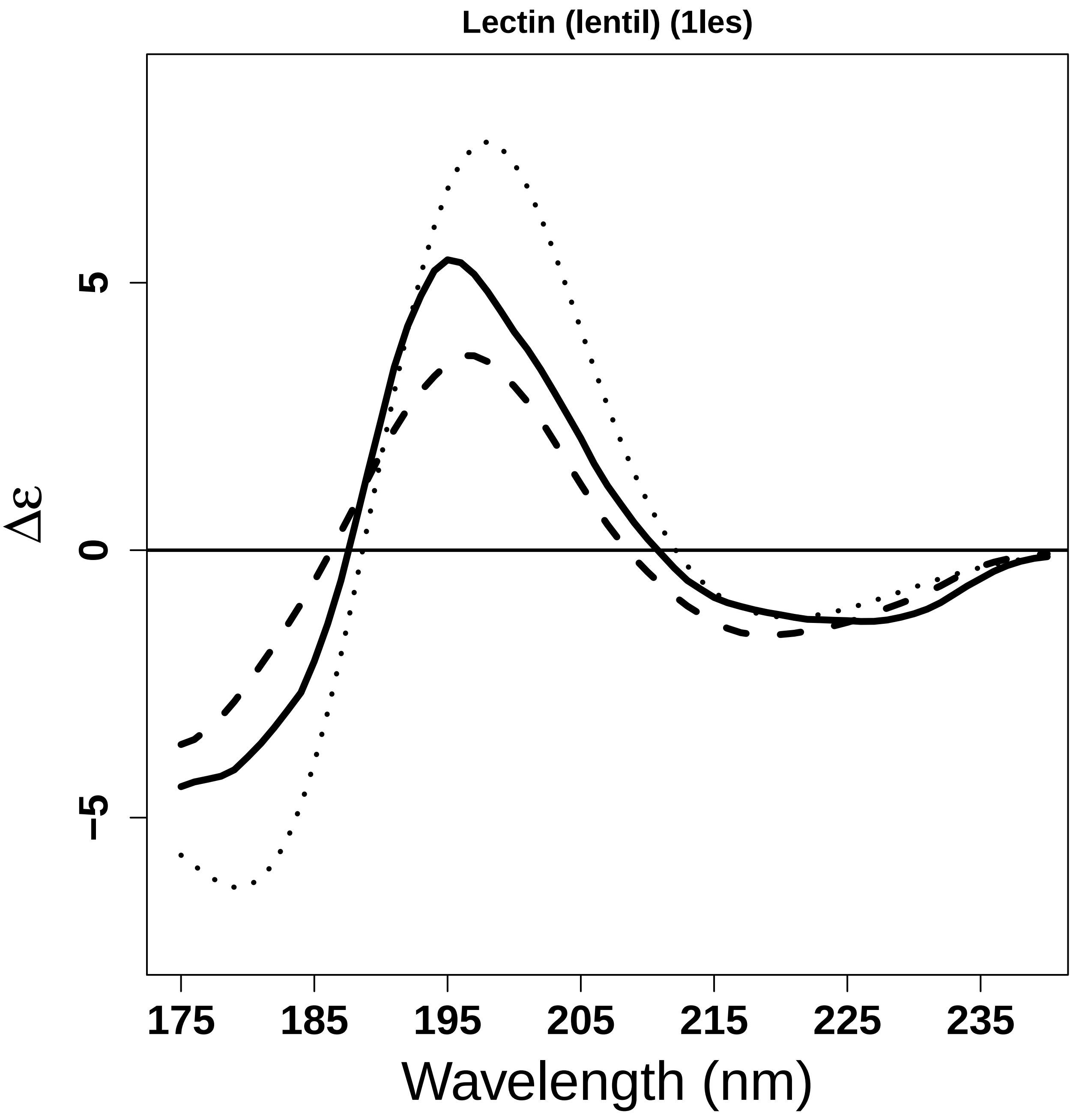

Lectin (lentil) (1les)
5
0
−5
Δε
175
185
195
205
215
225
235
Wavelength (nm)

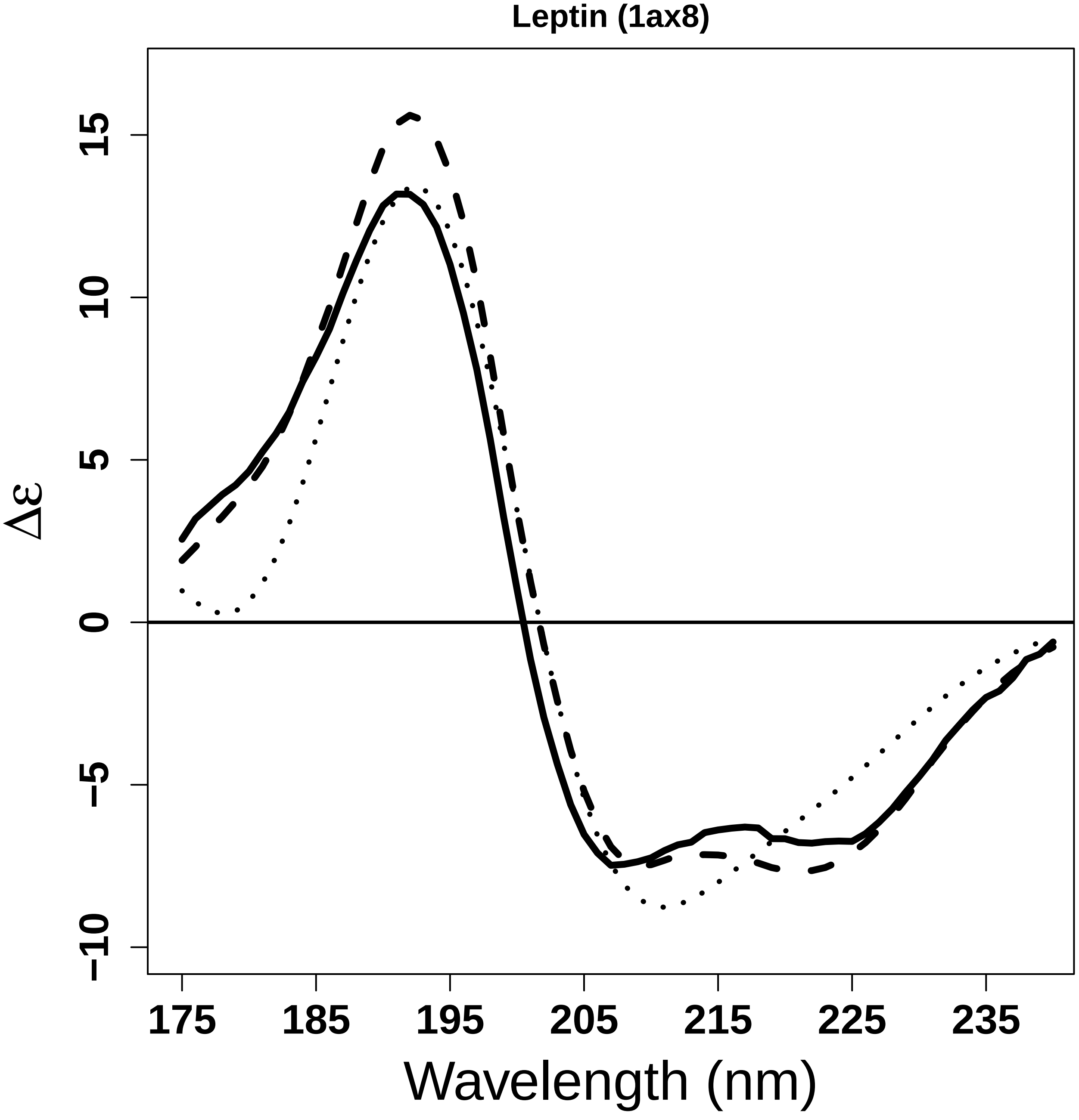

Leptin (1ax8)
Δε
15
10
5
0
−5
−10
175
185
195
205
215
225
235
Wavelength (nm)

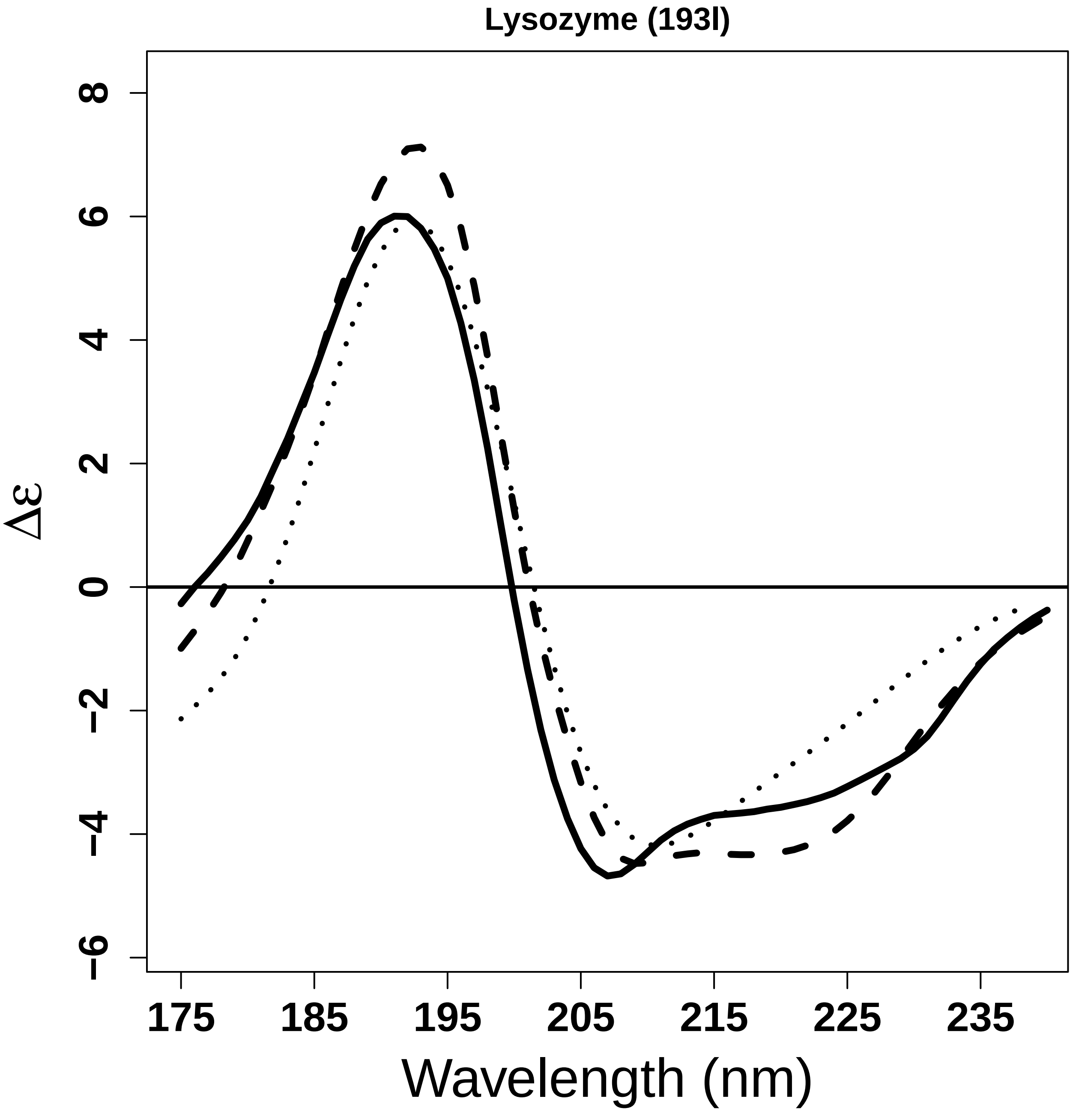

Lysozyme (193l)
Δε
8
6
4
2
0
−2
−4
−6
175
185
195
205
215
225
235
Wavelength (nm)

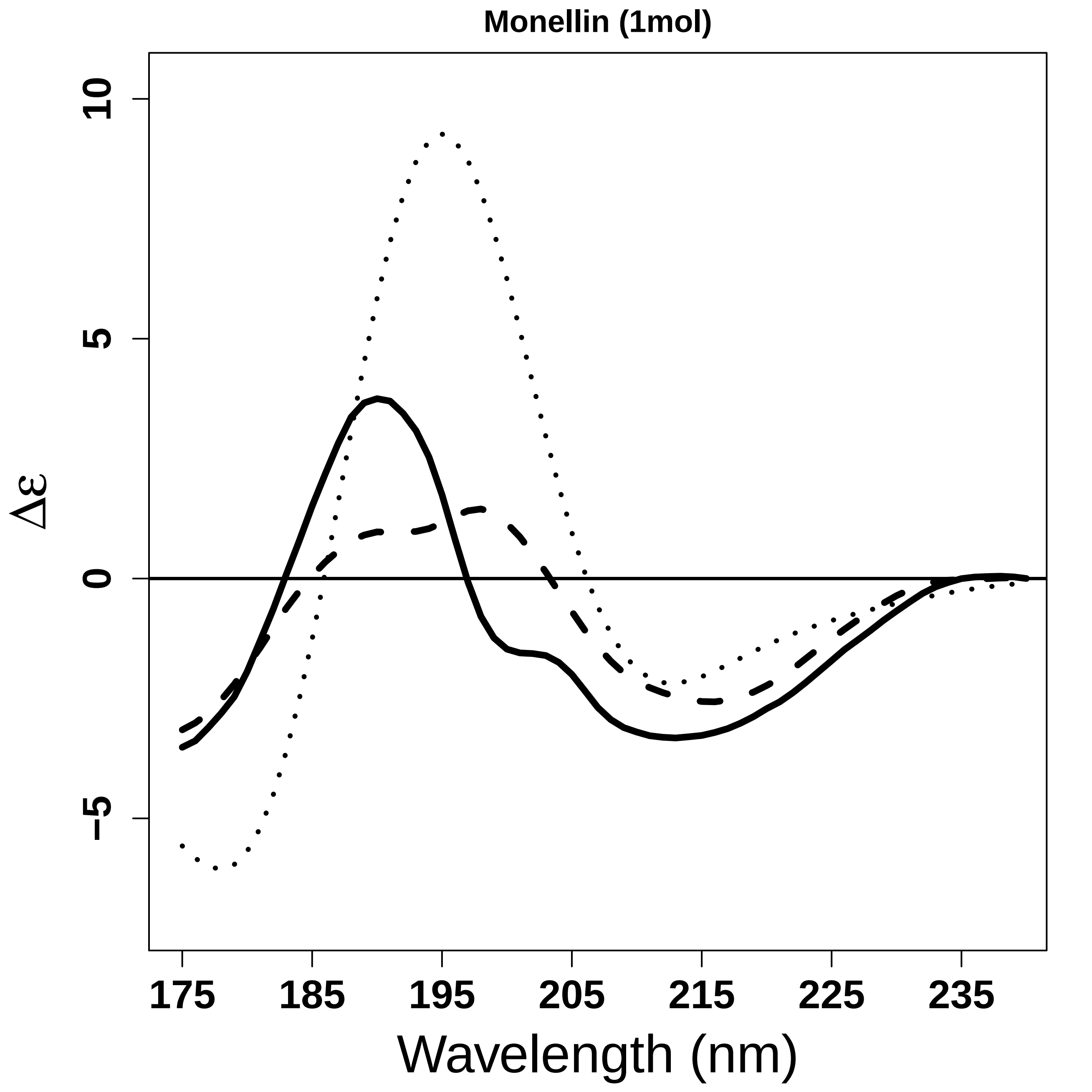

Monellin (1mol)
Δε
10
5
0
−5
175
185
195
205
215
225
235
Wavelength (nm)

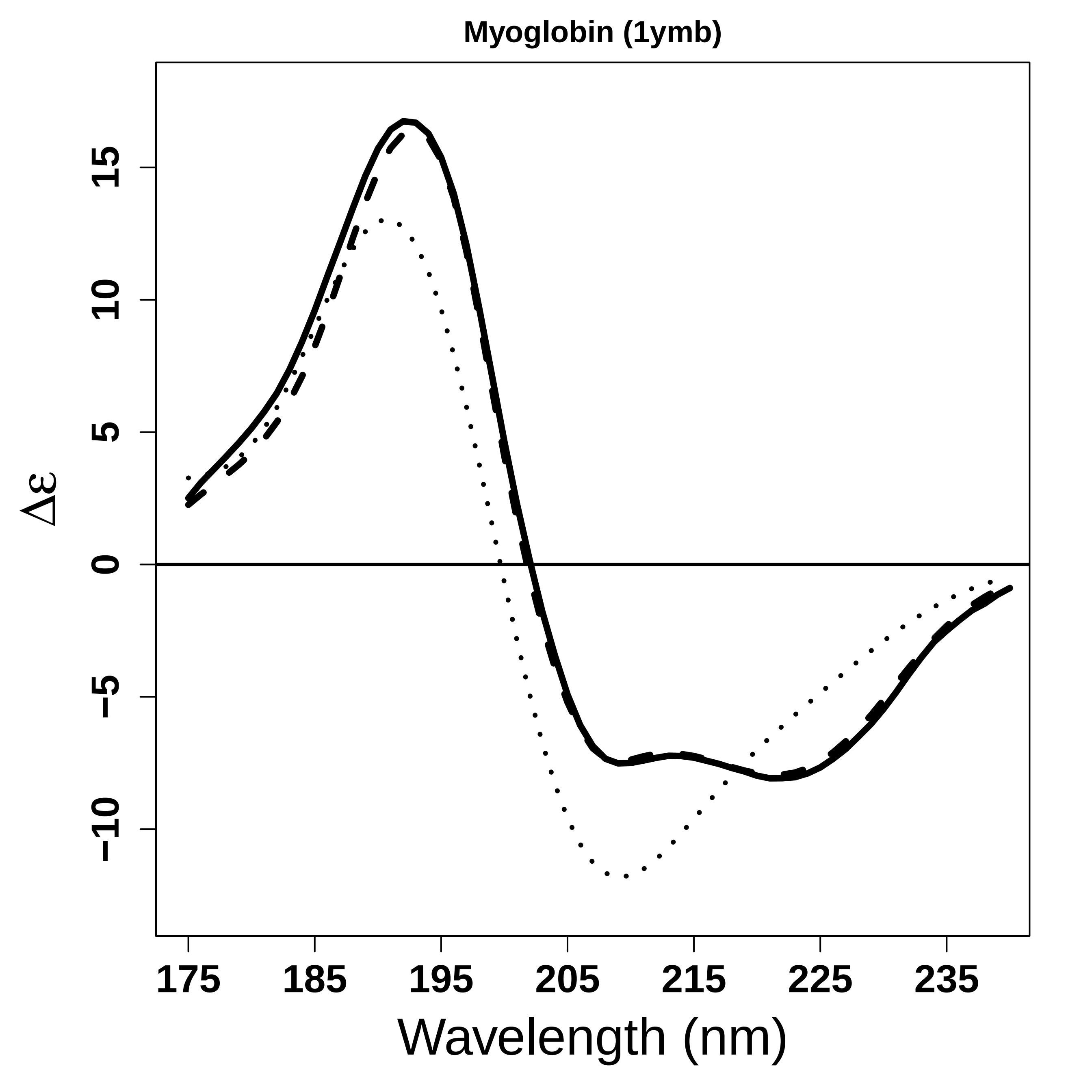

Myoglobin (1ymb)
15
10
5
0
−5
−10
Δε
175
185
195
205
215
225
235
Wavelength (nm)

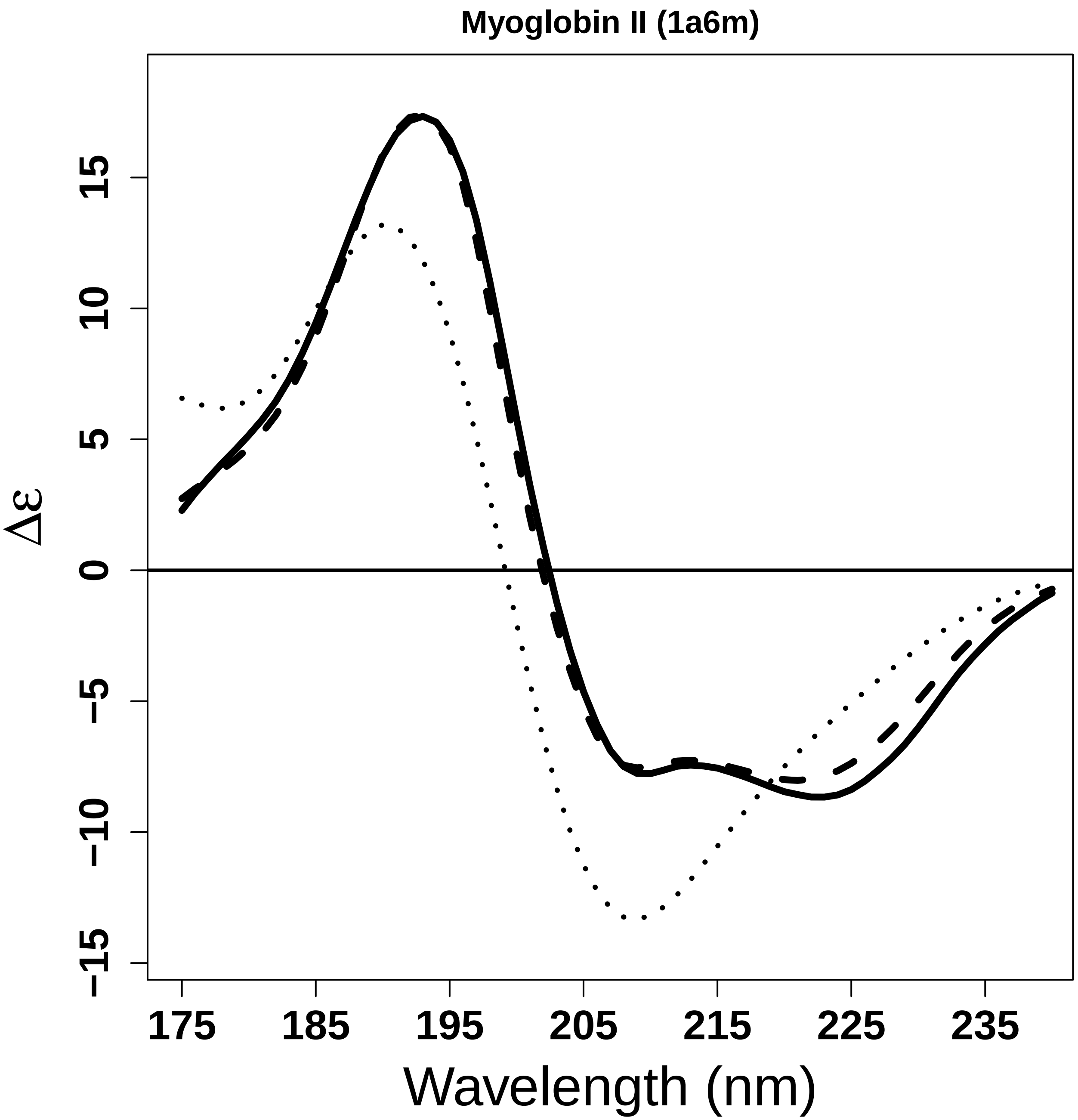

Myoglobin II (1a6m)
Δε
15
10
5
0
−5
−10
−15
175
185
195
205
215
225
235
Wavelength (nm)

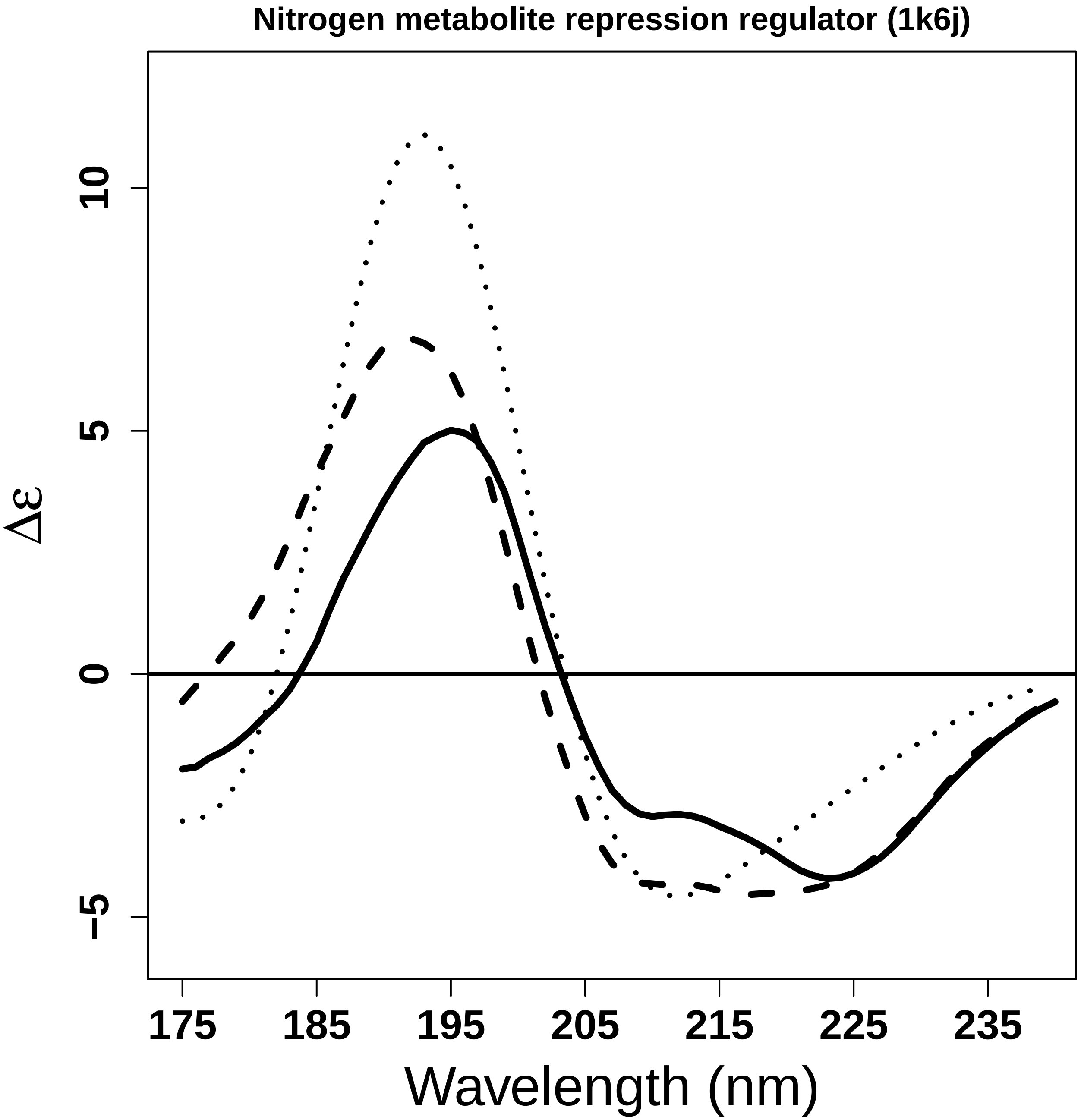

Nitrogen metabolite repression regulator (1k6j)
10
5
0
−5
Δε
175
185
195
205
215
225
235
Wavelength (nm)

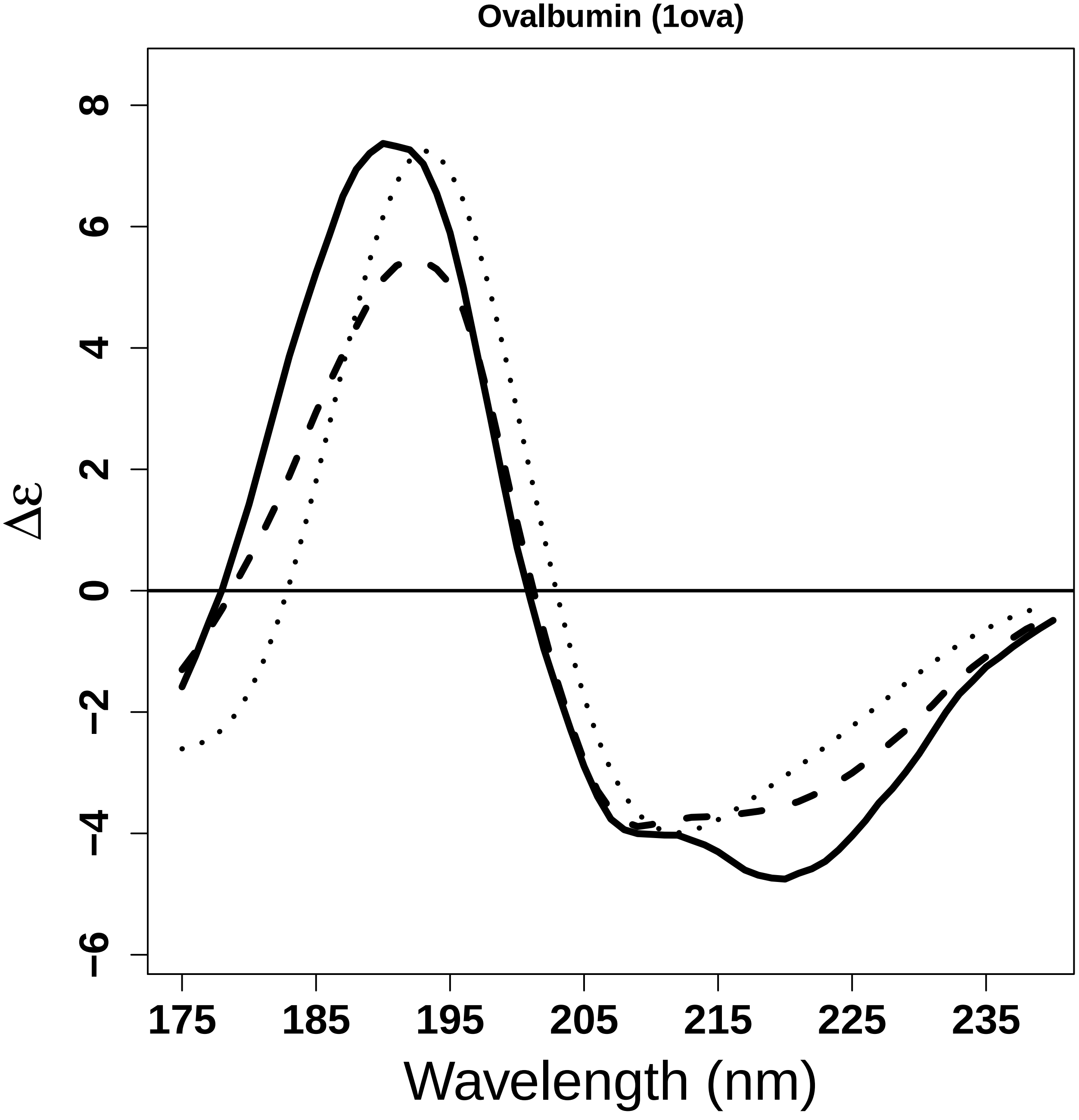

Ovalbumin (1ova)
Δε
8
6
4
2
0
−2
−4
−6
175
185
195
205
215
225
235
Wavelength (nm)

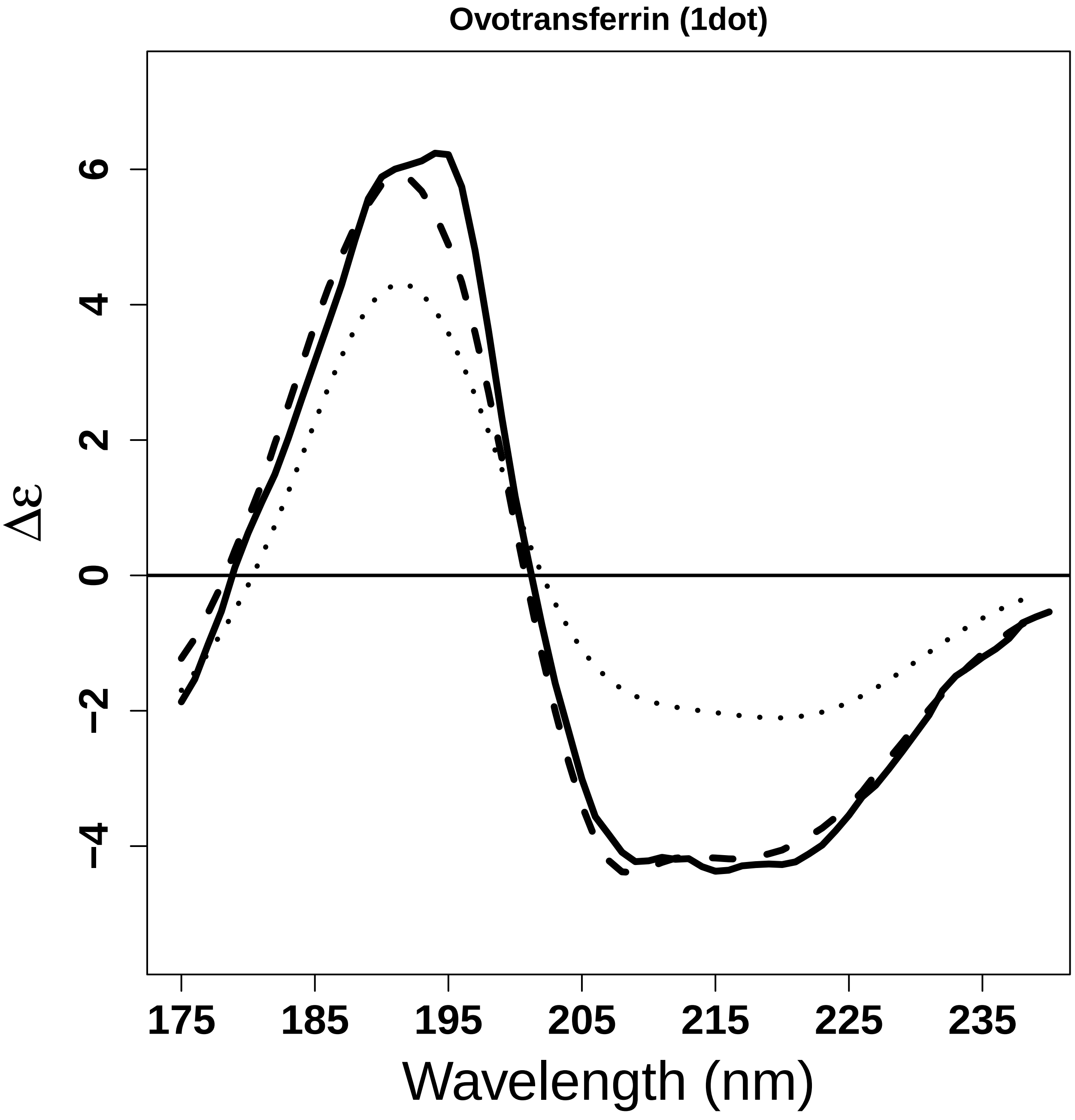

Ovotransferrin (1dot)
Δε
6
4
2
0
−2
−4
175
185
195
205
215
225
235
Wavelength (nm)

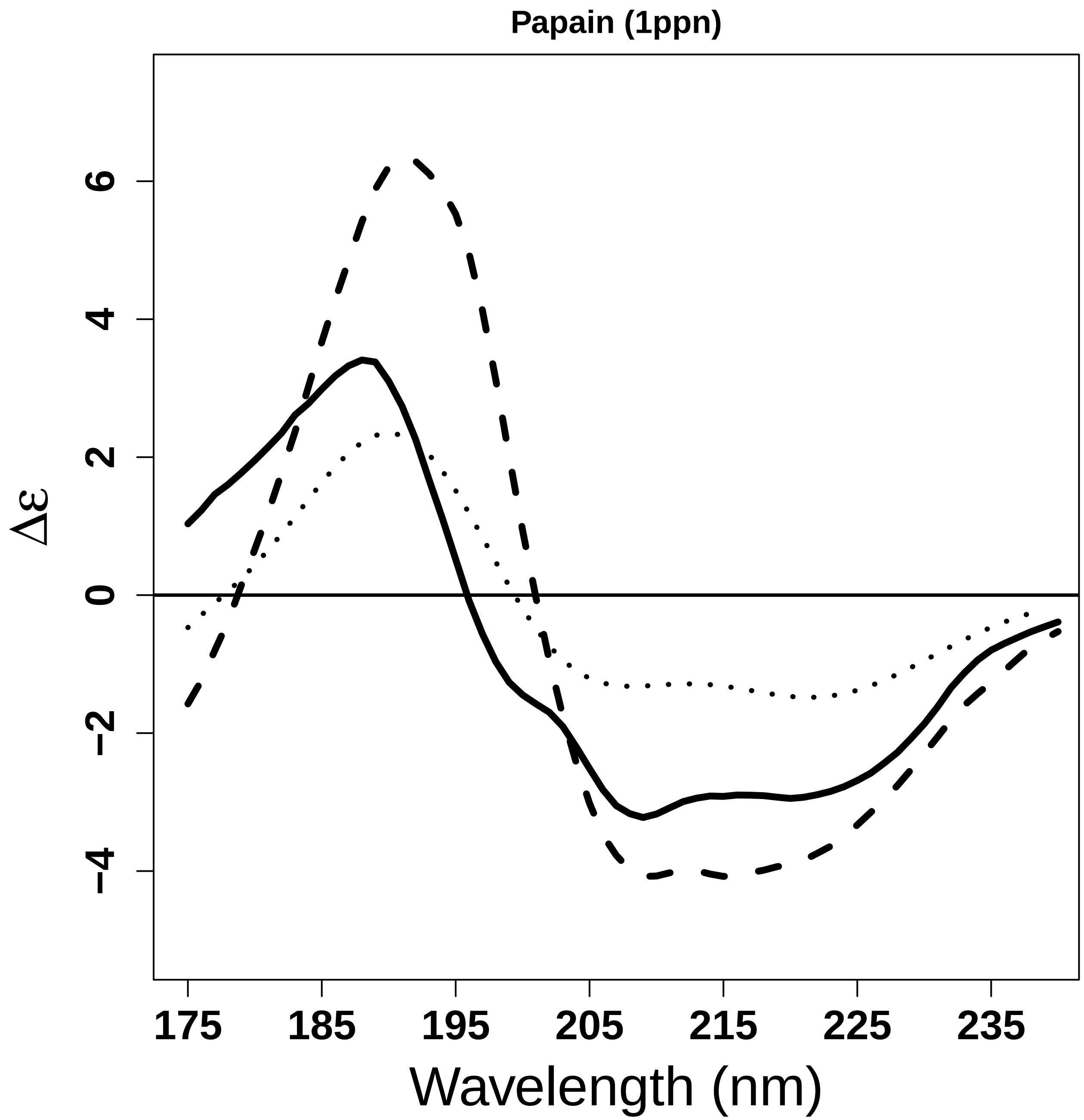

Papain (1ppn)
Δε
6
4
2
0
−2
−4
175
185
195
205
215
225
235
Wavelength (nm)

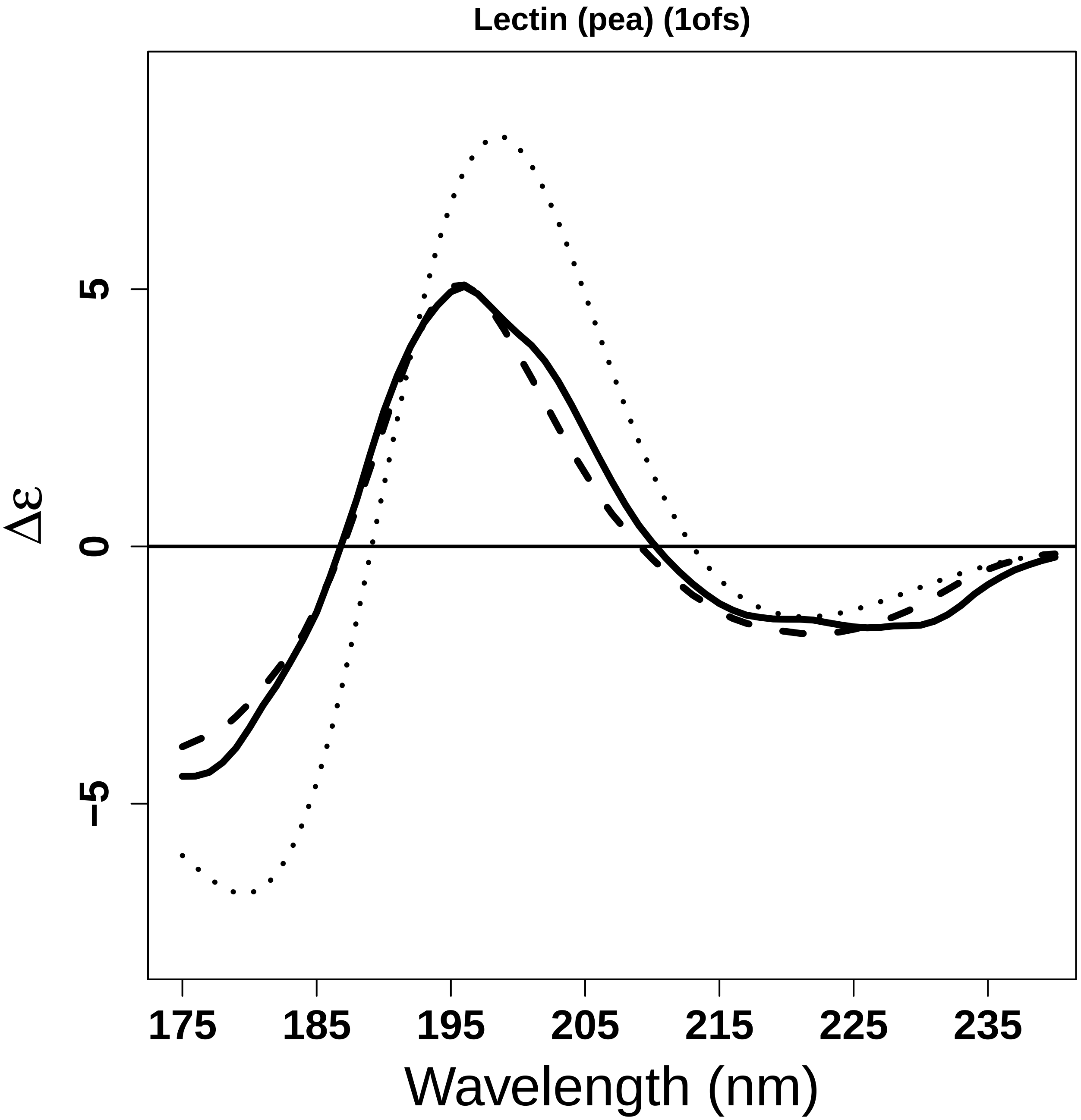

Lectin (pea) (1ofs)
Δε
5
0
−5
175
185
195
205
215
225
235
Wavelength (nm)

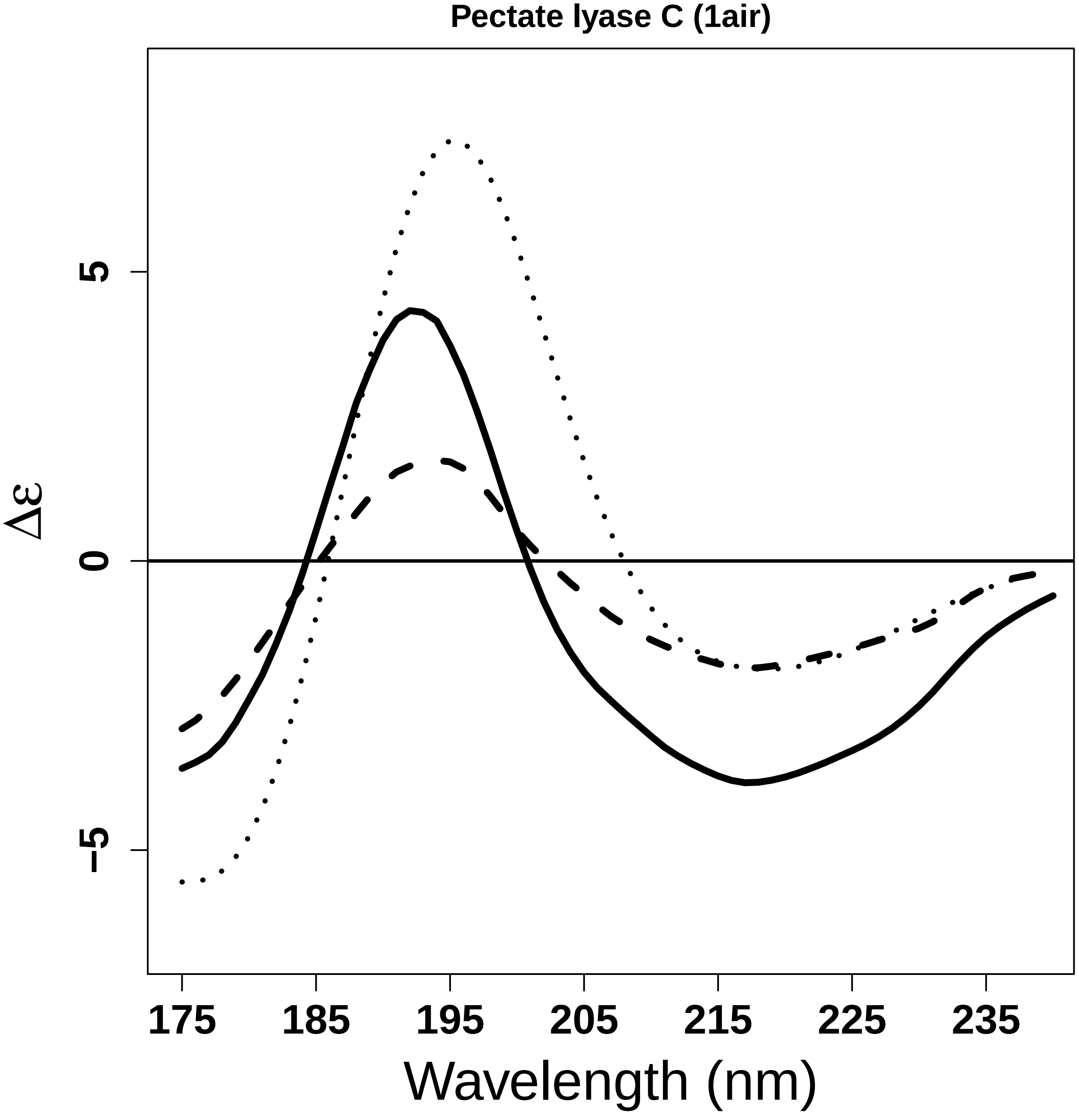

Pectate lyase C (1air)
5
0
−5
Δε
175
185
195
205
215
225
235
Wavelength (nm)

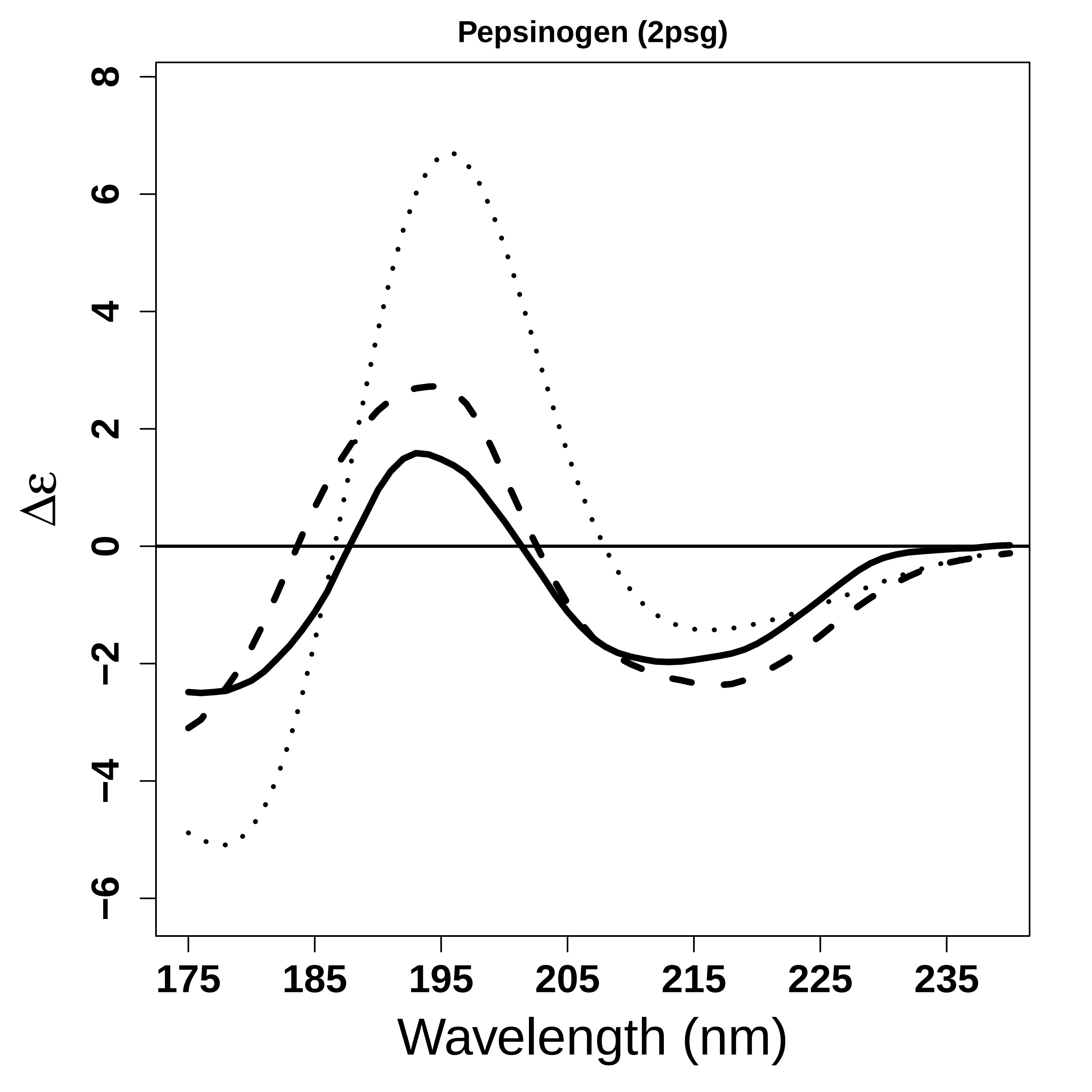

Pepsinogen (2psg)
8
6
4
2
0
−2
−4
−6
Δε
175
185
195
205
215
225
235
Wavelength (nm)

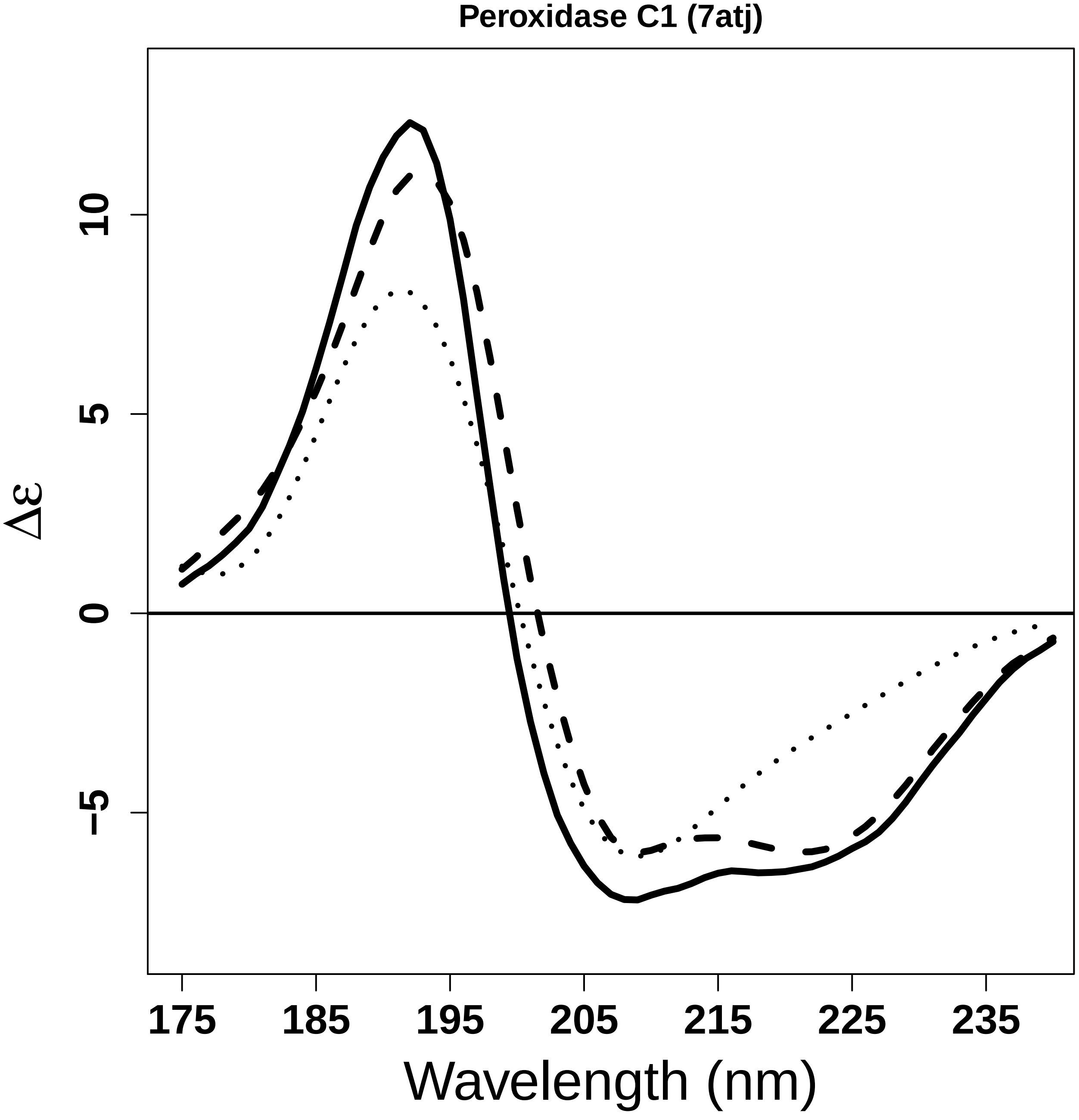

Peroxidase C1 (7atj)
Δε
10
5
0
−5
175
185
195
205
215
225
235
Wavelength (nm)

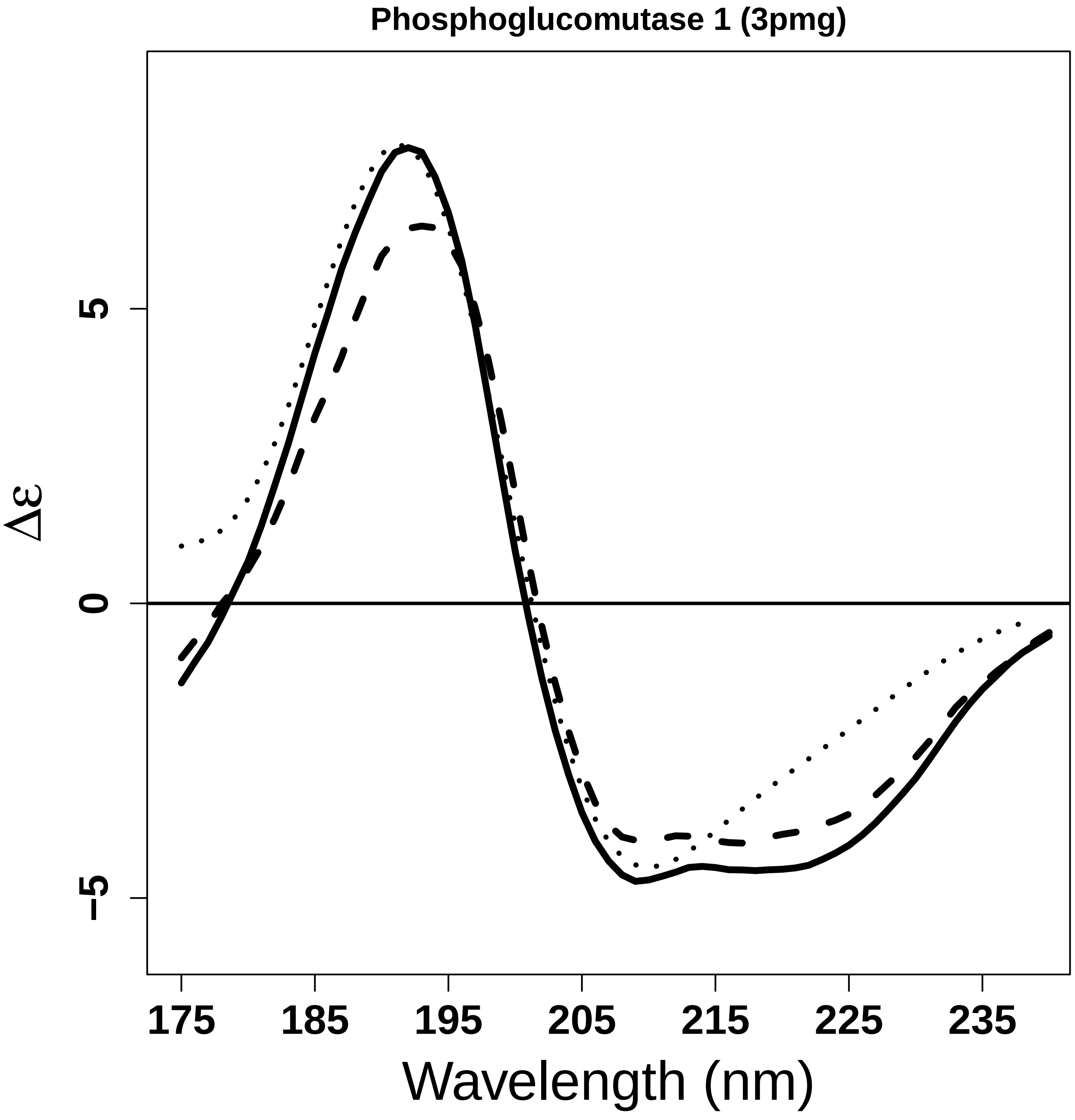

Phosphoglucomutase 1 (3pmg)
Δε
5
0
−5
175
185
195
205
215
225
235
Wavelength (nm)

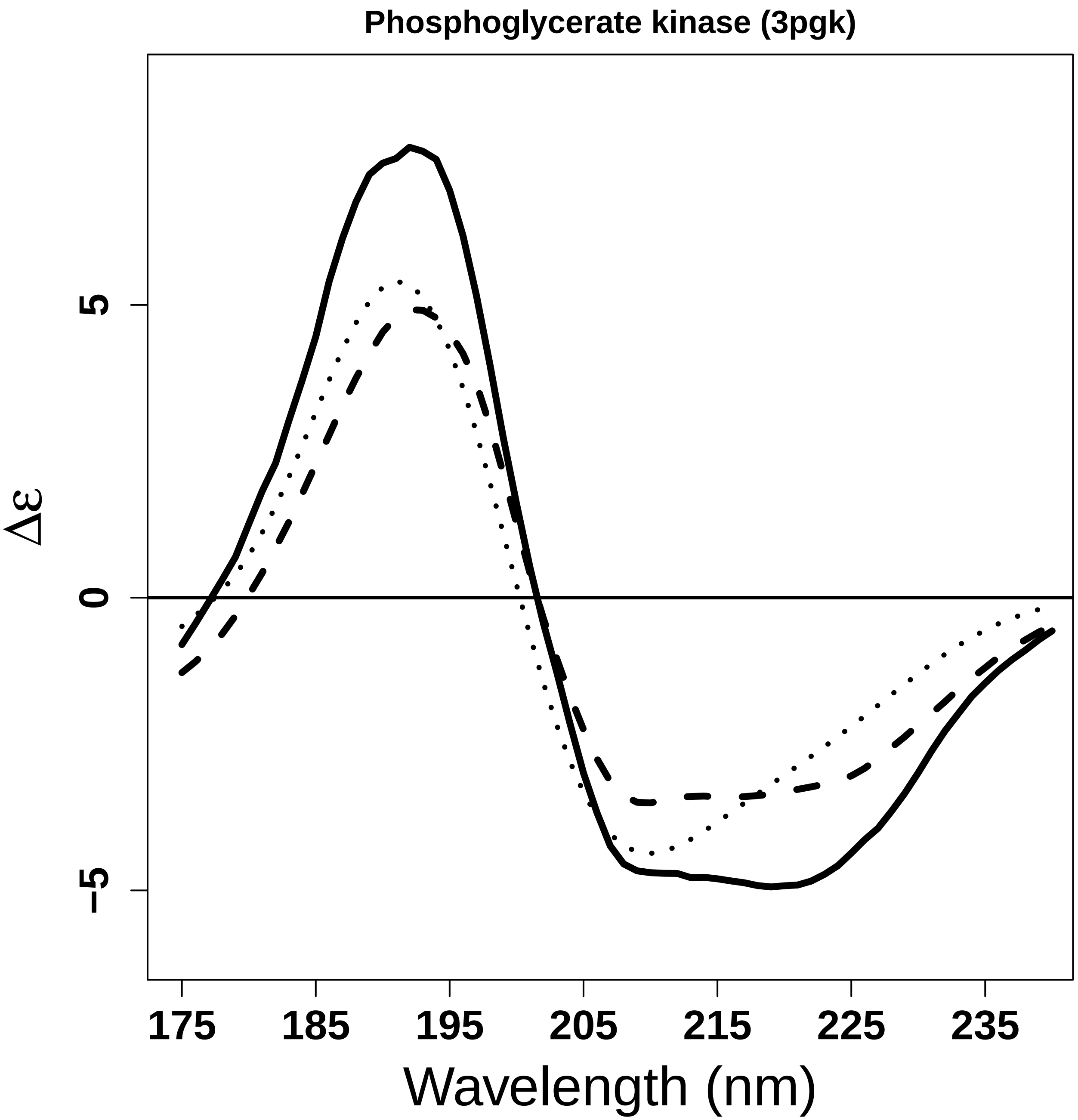

Phosphoglycerate kinase (3pgk)
Δε
5
0
−5
175
185
195
205
215
225
235
Wavelength (nm)

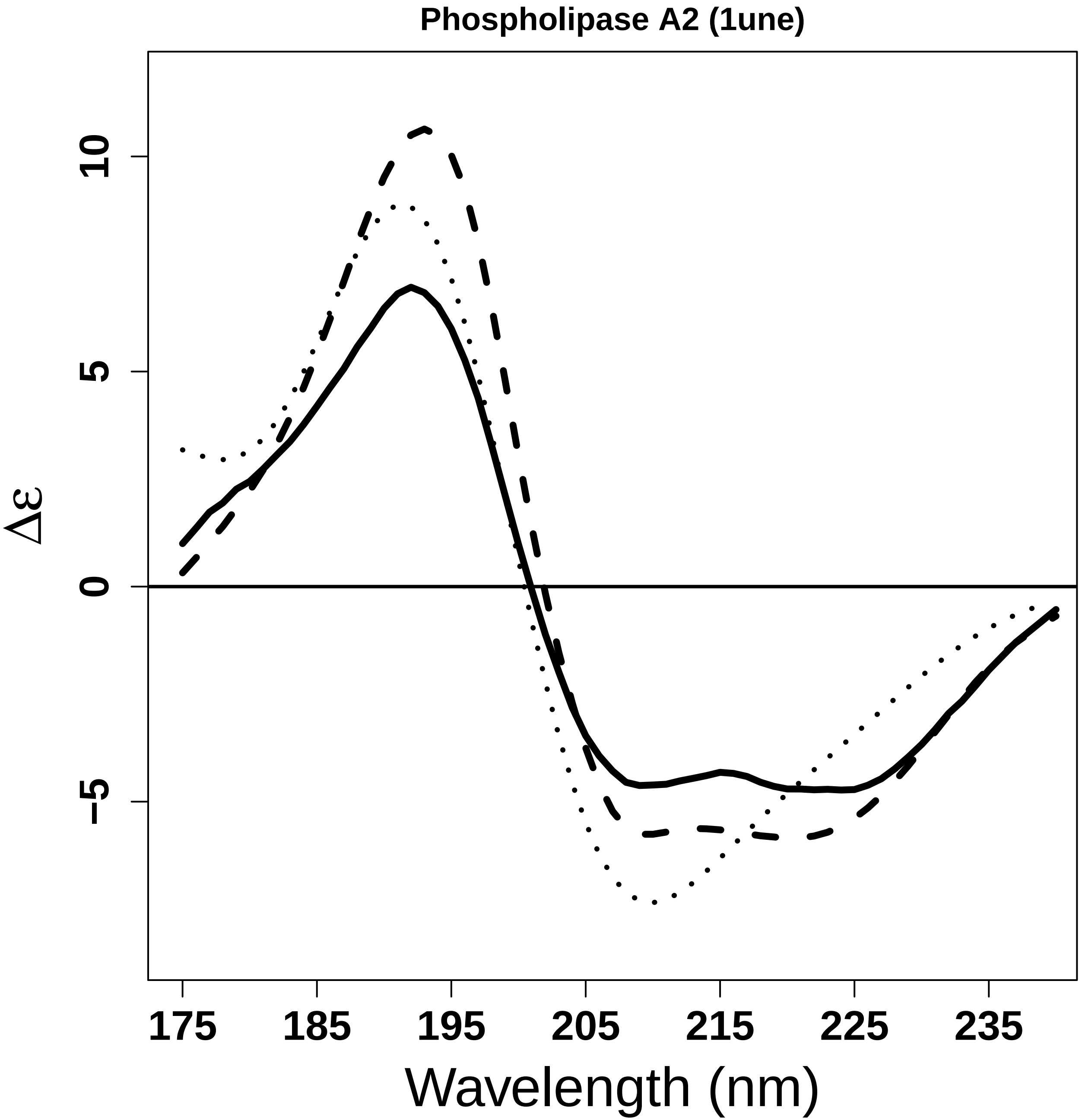

Phospholipase A2 (1une)
10
5
0
−5
Δε
175
185
195
205
215
225
235
Wavelength (nm)

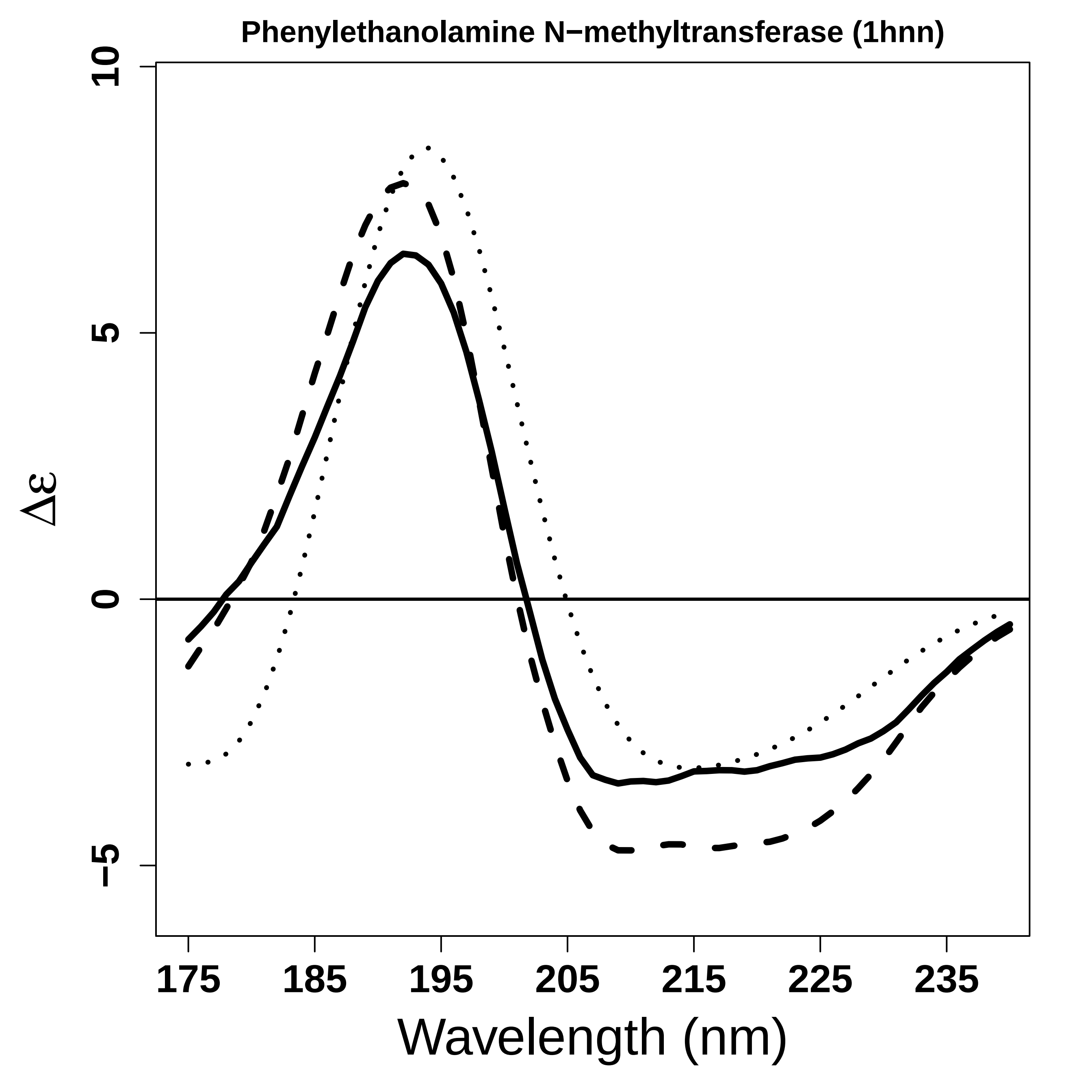

Phenylethanolamine N−methyltransferase (1hnn)
10
5
0
−5
Δε
175
185
195
205
215
225
235
Wavelength (nm)

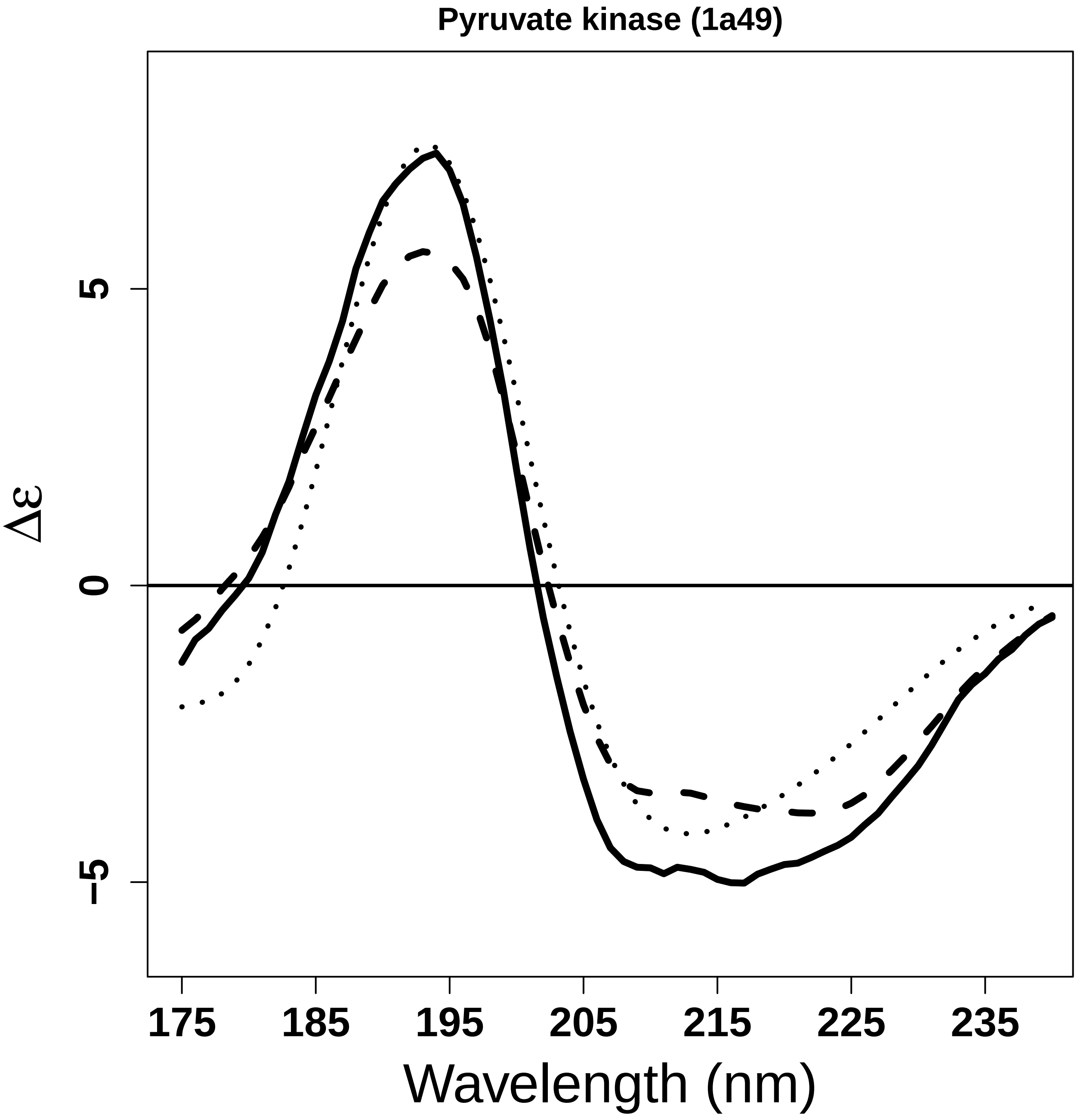

Pyruvate kinase (1a49)
Δε
5
0
−5
175
185
195
205
215
225
235
Wavelength (nm)

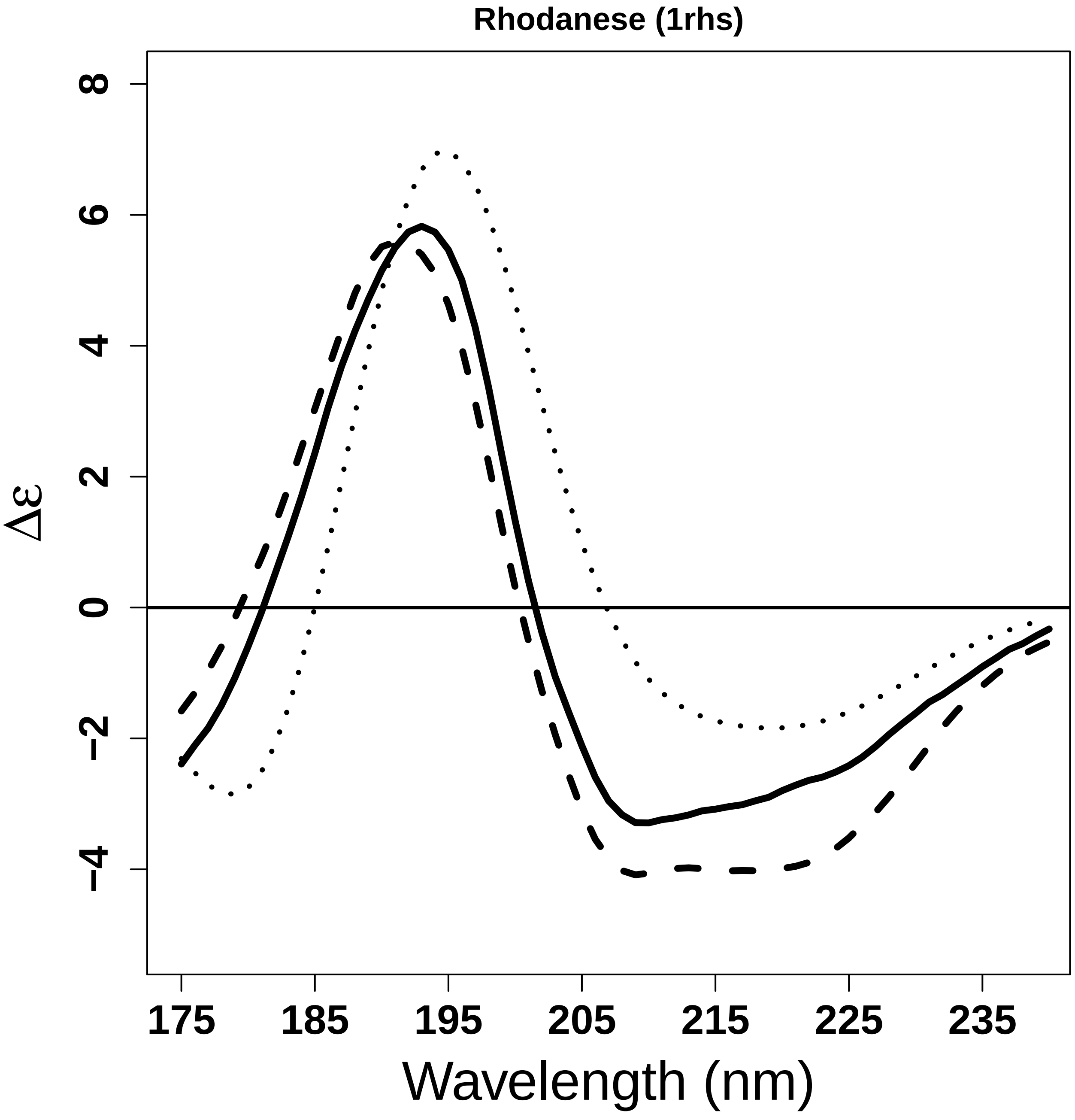

Rhodanese (1rhs)
Δε
8
6
4
2
0
−2
−4
175
185
195
205
215
225
235
Wavelength (nm)

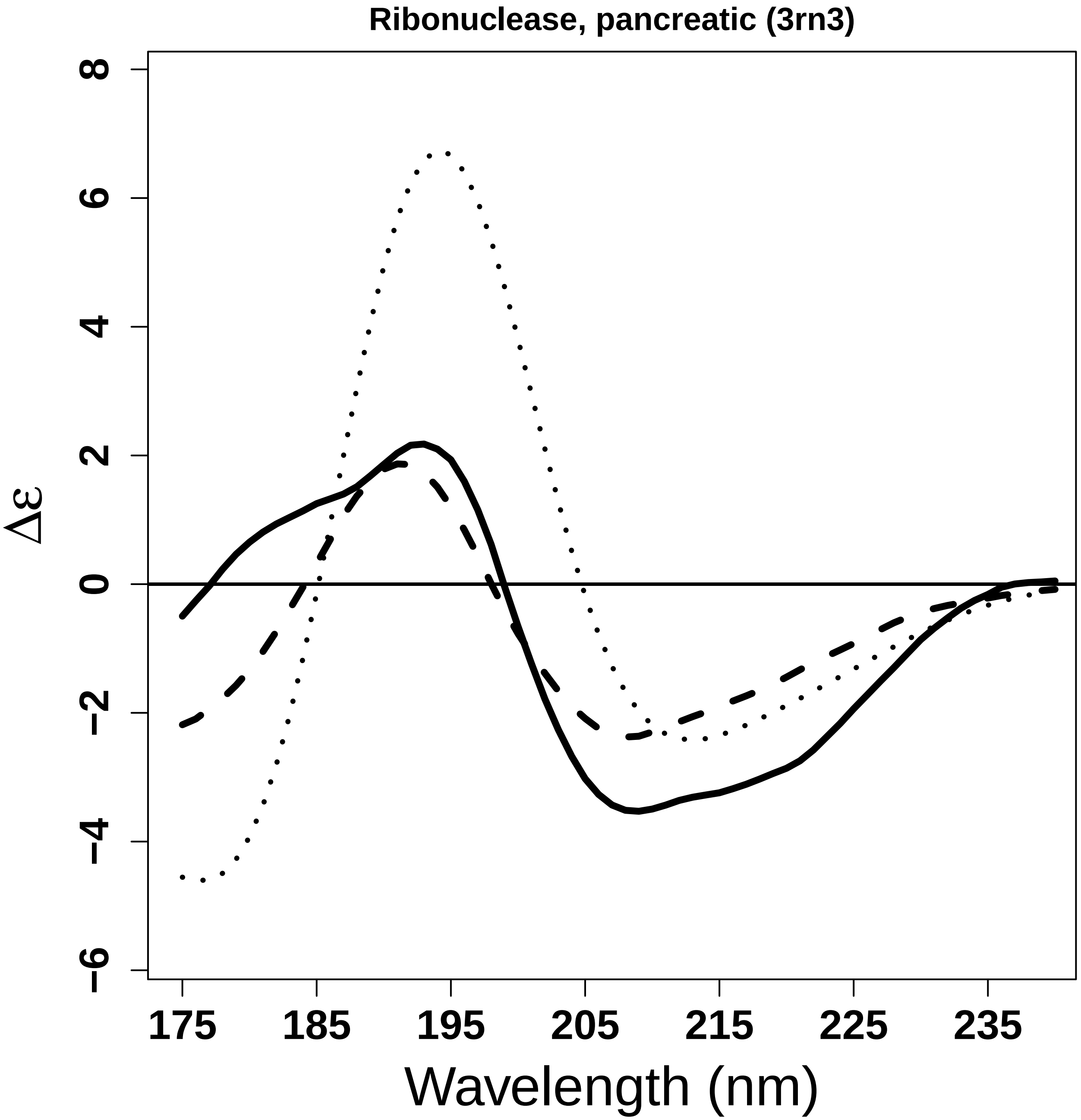

Ribonuclease, pancreatic (3rn3)
Δε
8
6
4
2
0
−2
−4
−6
175
185
195
205
215
225
235
Wavelength (nm)

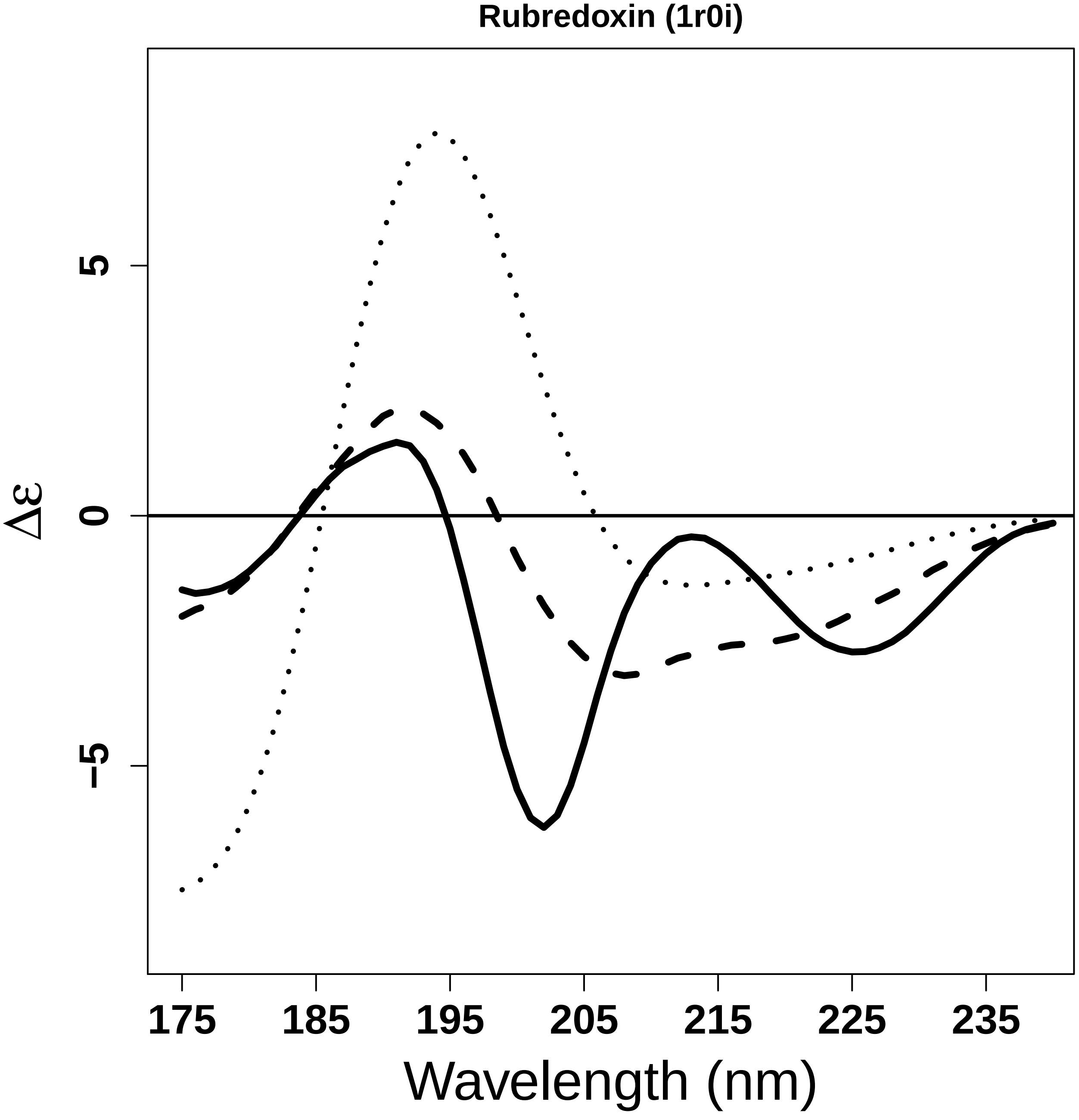

Rubredoxin (1r0i)
Δε
5
0
−5
175
185
195
205
215
225
235
Wavelength (nm)

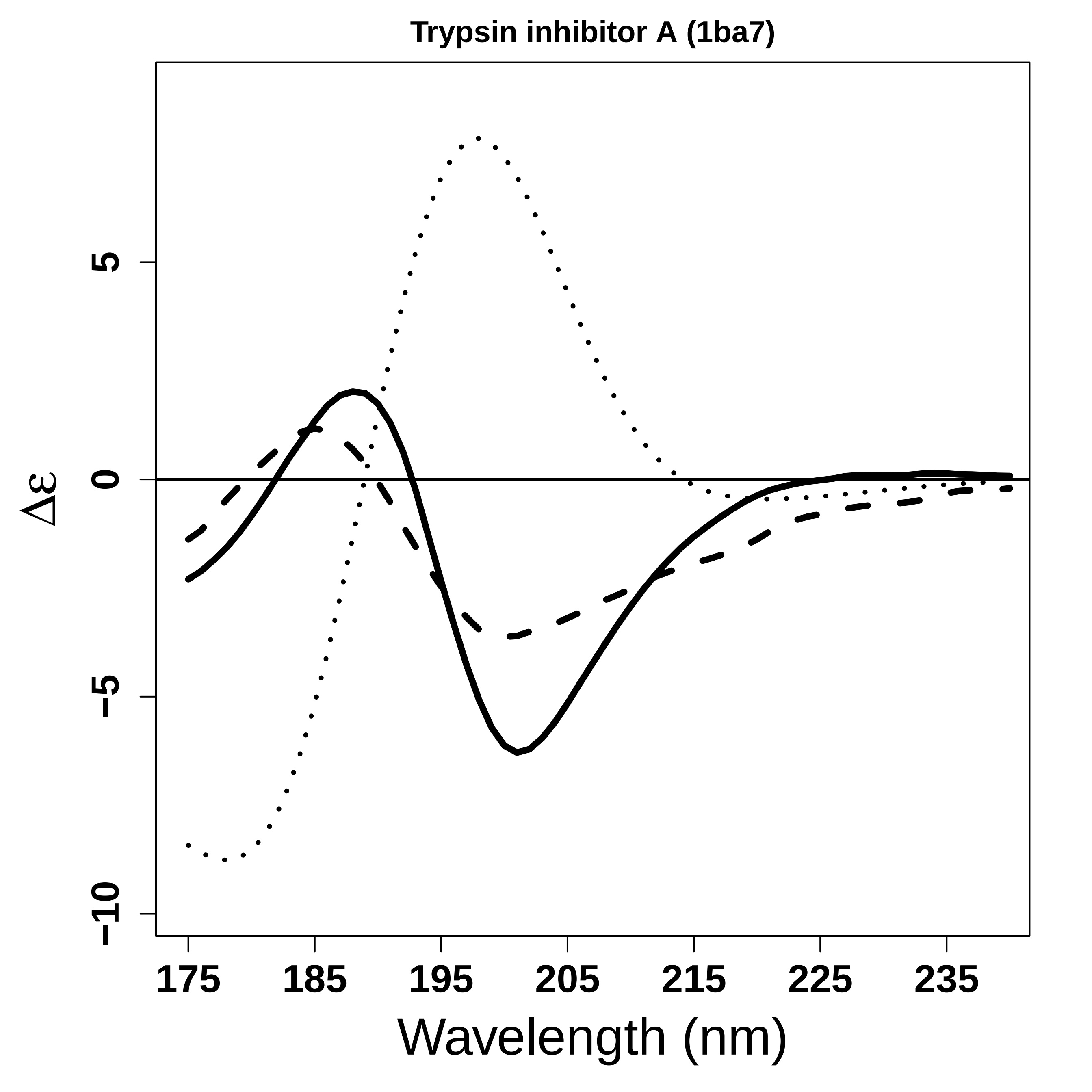
Trypsin inhibitor A (1ba7)
5
0
−5
−10
Δε
175
185
195
205
215
225
235
Wavelength (nm)

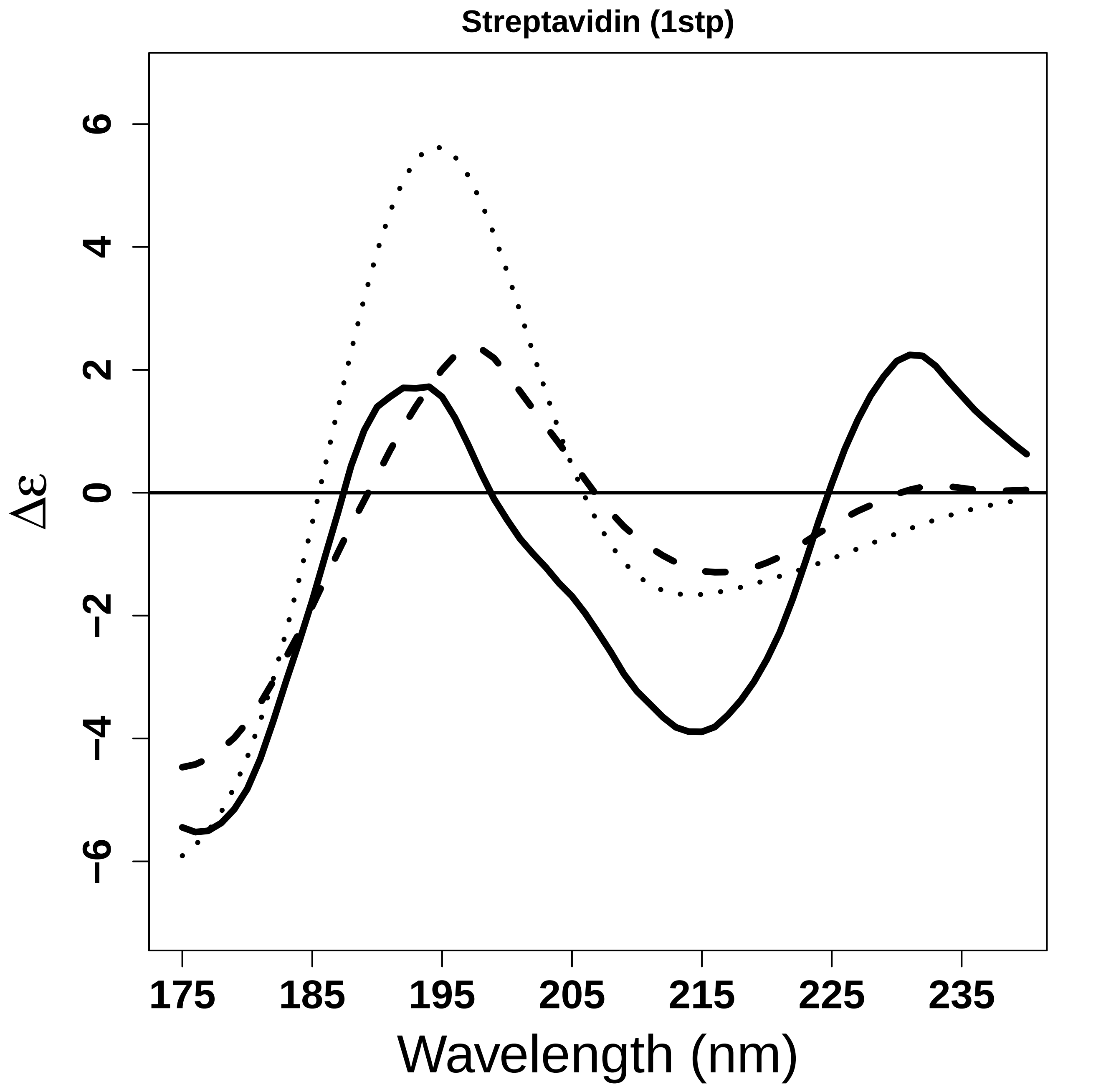

Streptavidin (1stp)
Δε
6
4
2
0
−2
−4
−6
175
185
195
205
215
225
235
Wavelength (nm)

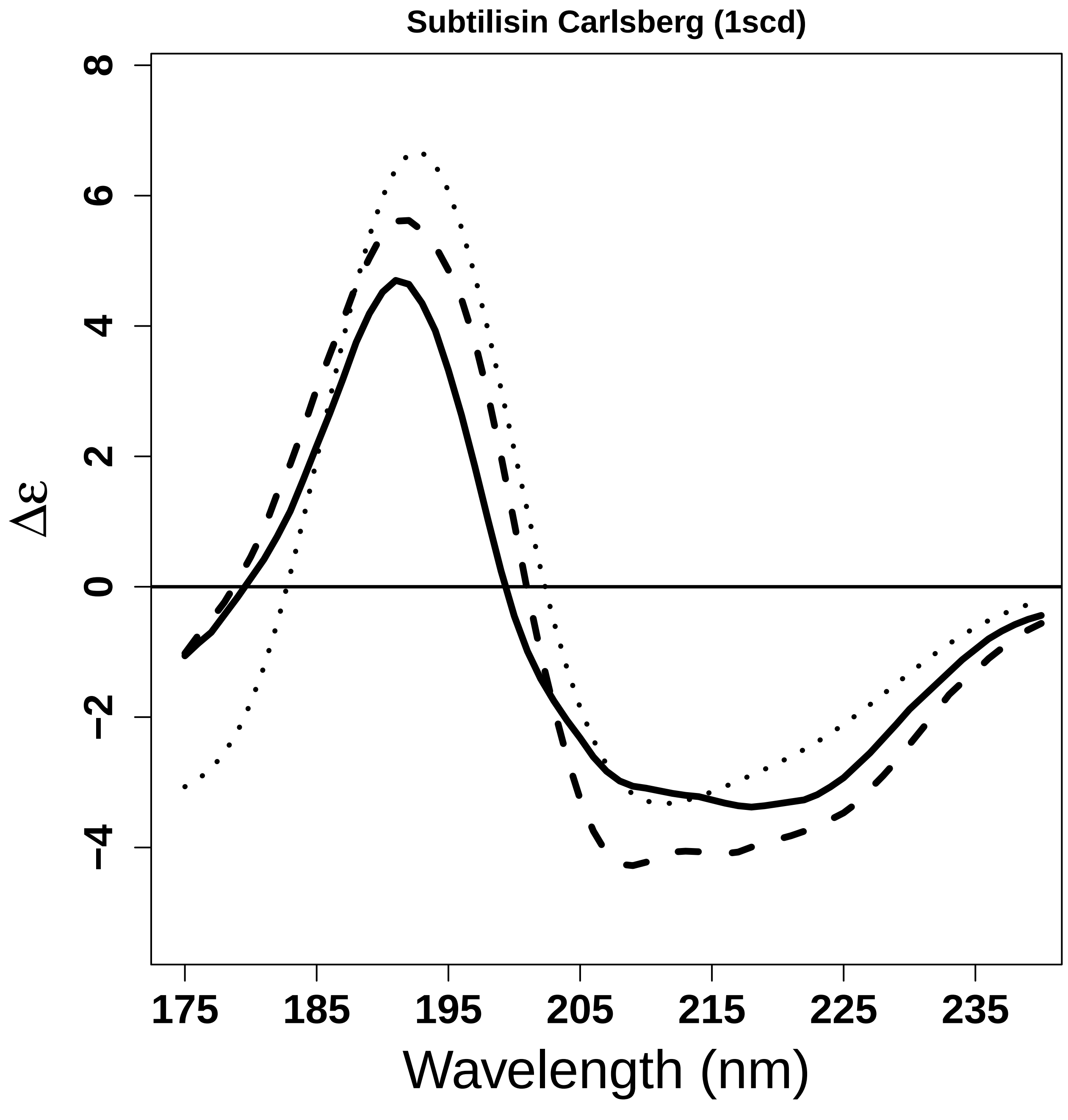

Subtilisin Carlsberg (1scd)
8
6
4
2
0
−2
−4
Δε
175
185
195
205
215
225
235
Wavelength (nm)

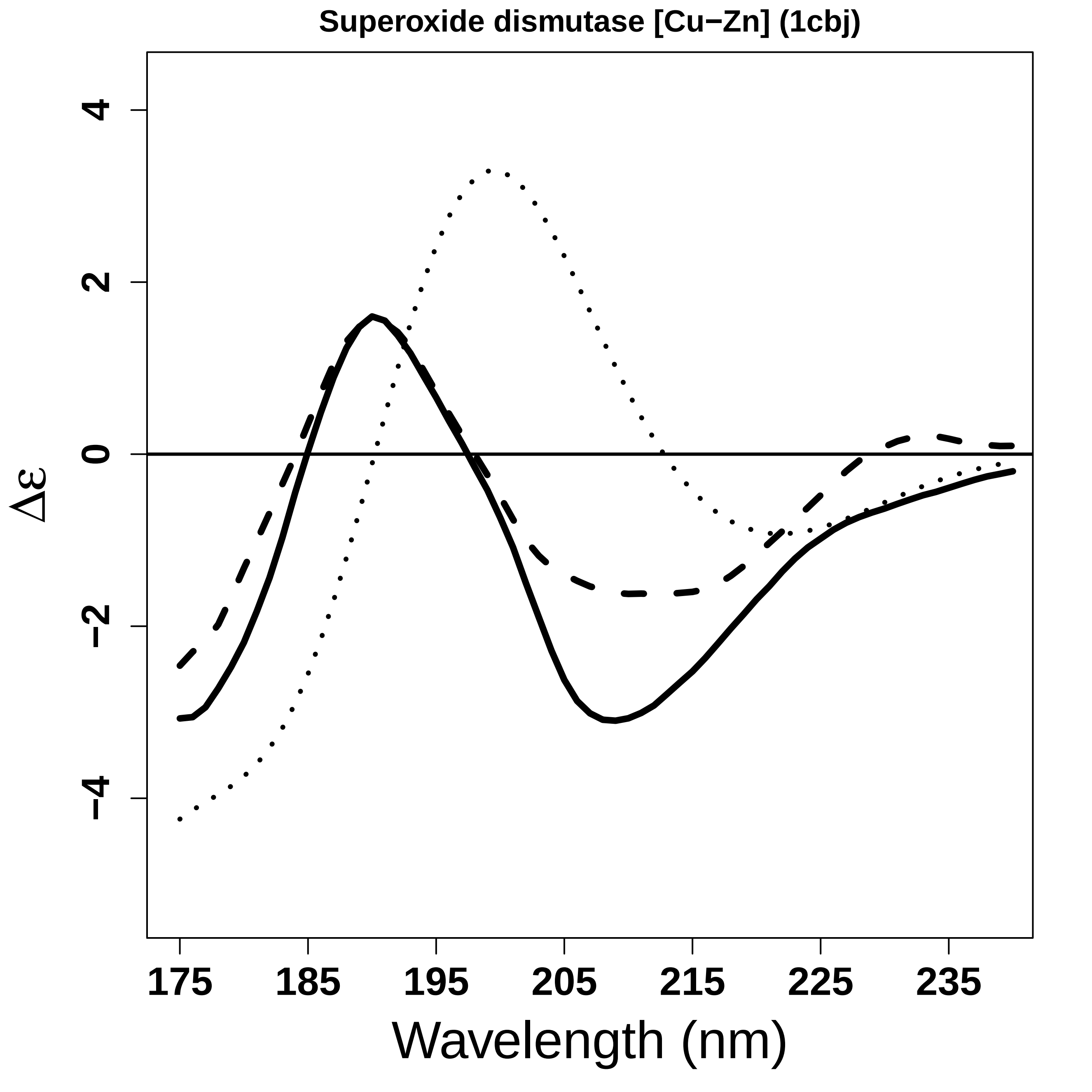

Superoxide dismutase [Cu−Zn] (1cbj)
4
2
0
−2
−4
Δε
175
185
195
205
215
225
235
Wavelength (nm)

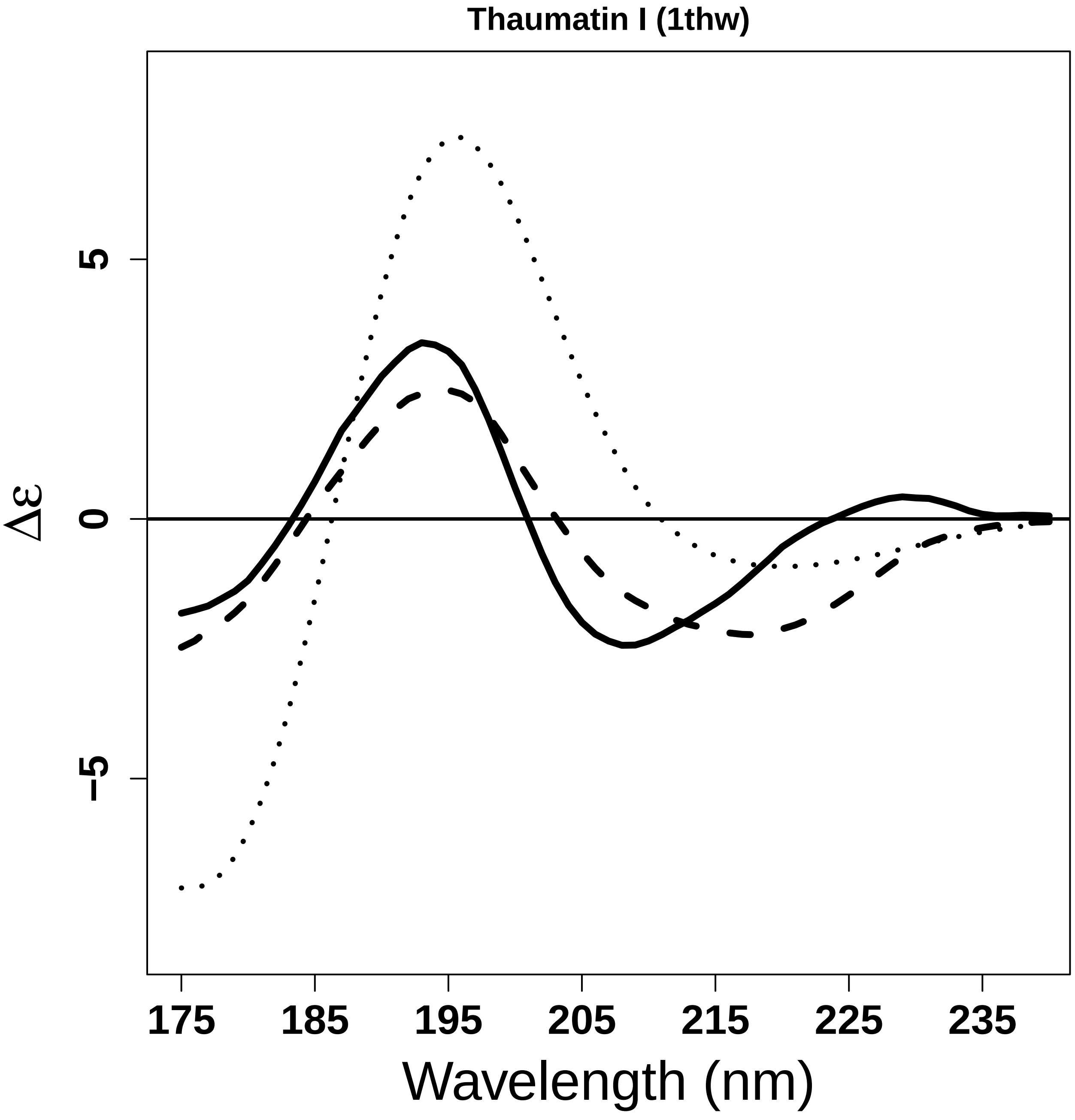

Thaumatin I (1thw)
Δε
5
0
−5
175
185
195
205
215
225
235
Wavelength (nm)

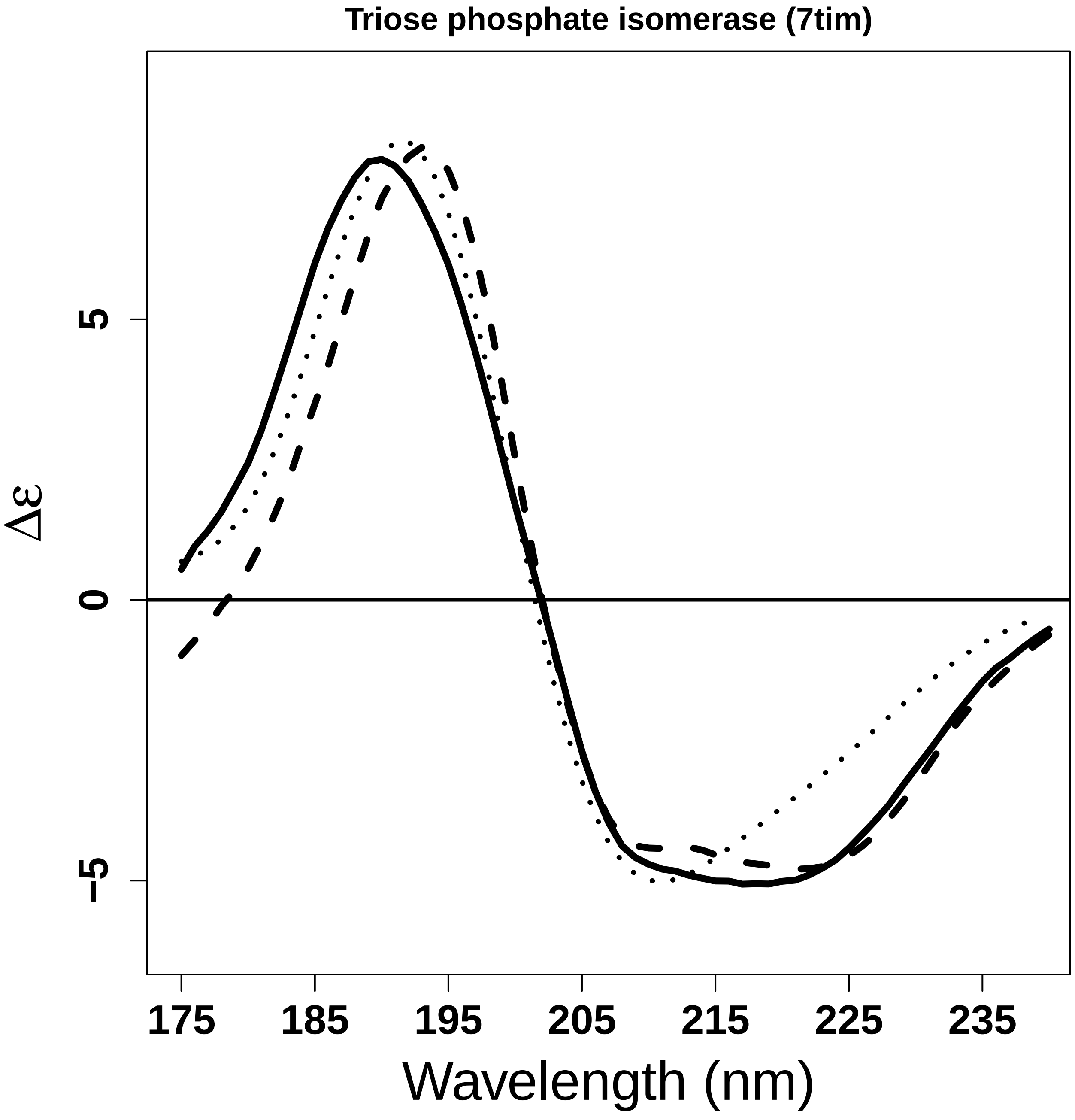
Triose phosphate isomerase (7tim)
Δε
5
0
−5
175
185
195
205
215
225
235
Wavelength (nm)

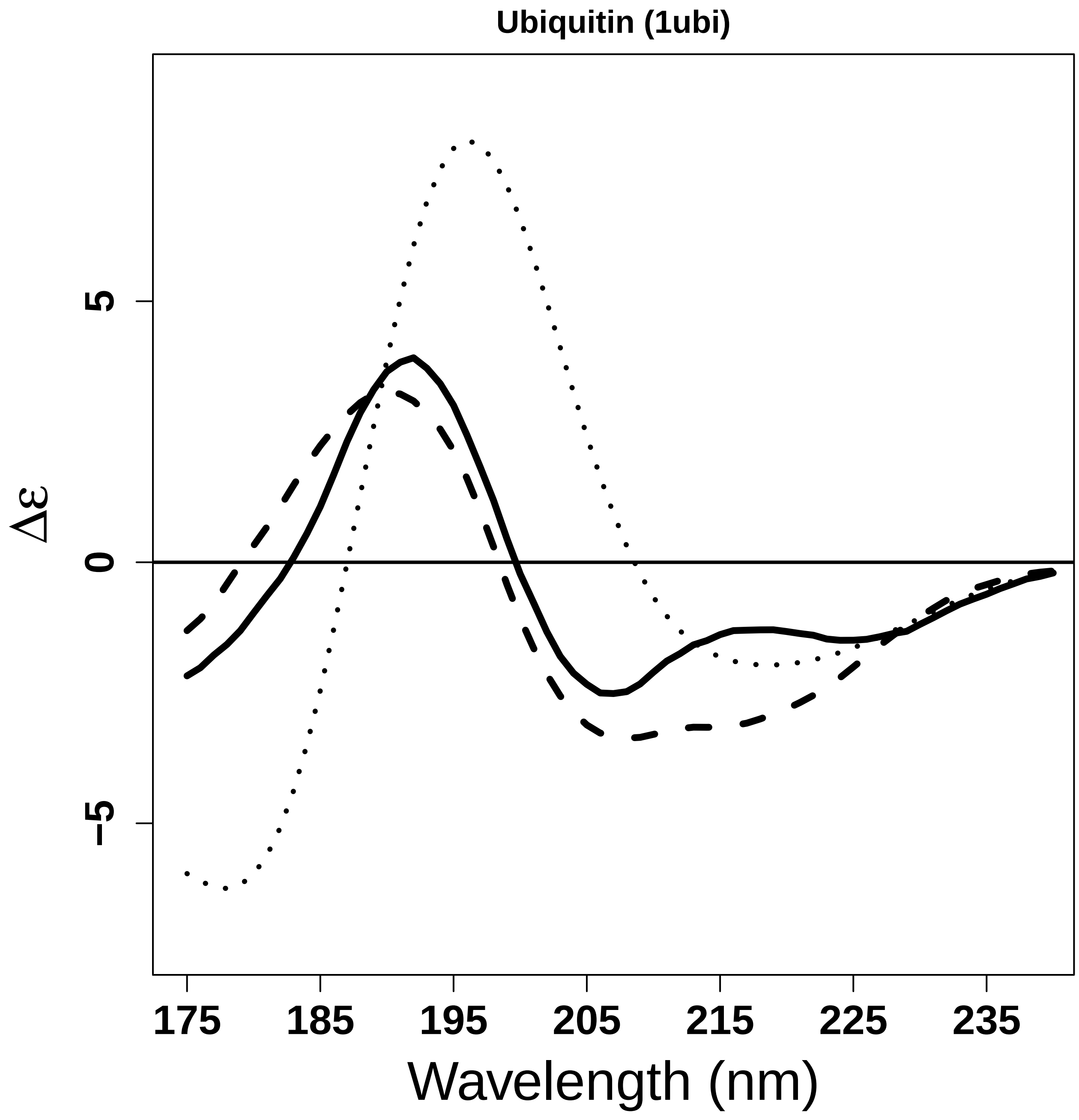
Ubiquitin (1ubi)
Δε
5
0
−5
175
185
195
205
215
225
235
Wavelength (nm)
