# Supplementary material for: PDB2CD: a web-based application for the generation of circular dichroism spectra from protein atomic coordinates
Source: Bioinformatics. 2016 Sep 20;33(1):56–63. doi: 10.1093/bioinformatics/btw554 (PMC5408769; doi:10.1093/bioinformatics/btw554)
Supplement: Supplementary Data [file btw554_supp.zip › Protein_CATH.pdf]

Protein Name	PDB Code	CATH CLASSES
Aldolase	1ado	3.20.20.70
Alkaline_phosphatase	1ed9	3.40.720.10
Alpha_amylase	1vjs	2.60.40.1180
Alpha_bungarotoxin	1hc9	2.10.60.10
Alpha_chymotrypsin	5cha	2.40.10.10
Alpha_chymotrypsinogen	2cga	2.40.10.10
Aprotinin	5pti	4.10.410.10
Avidin	1rav	2.40.128.30
Beta_amylase	1fa2	3.20.20.80
Beta_galactosidase	1bgl	2.60.120.260<br>2.60.40.320<br>3.20.20.80<br>2.60.40.320<br>2.70.98.10
Beta_lactoglobulin	1b8e	2.40.128.20
c-Phycocyanin	1ha7	1.10.490.20
Calmodulin	1lin	1.10.238.10
Carbonic_anhydrase_I	1hcb	3.10.200.10
Carbonic_anhydrase_II	1v9e	3.50.50.60<br>3.30.519.10<br>1.10.405.10
Carboxypeptidase_A1	5cpa	3.40.630.10
Catalase	1dgf	-
Ceruloplasmin	1kcw	2.60.40.420
Citrate_synthase	2cts	1.10.580.10<br>1.10.230.10
Concanavalin_A	1nls	2.60.120.200
Cytochrome_C	1hrc	1.10.760.10
Beta-B2_crystallin	2bb2	2.60.20.10
Gamma-B_crystallin	4gcr	2.60.20.10
Gamma-D_crystallin	1elp	2.60.20.10
Gamma-E-crystallin	1m8u	2.60.20.10
Gamma-s-crystallin_C_terminus	1ha4	2.60.20.10
Gamma_D_crystallin_(WT)	1hk0	2.60.20.10
3-Dehydroquinate_dehydratase	1qfe	3.20.20.70
3-Dehydroquinate_dehydratase_0	2dhq	3.40.50.9100
Deoxyribonuclease-1	3dni	3.60.10.10
Elastase	3est	2.40.10.10
Ferredoxin	2fdn	3.30.70.20
Glucose_oxidase	1cf3	3.50.50.60<br>4.10.450.10<br>3.30.560.10

Glutamate_dehydrogenase_I	3mw9	1.10.287.140<br>3.40.192.10<br>3.40.50.720
Glycogen_phosphorylase_b	1gpb	3.40.50.2000
Haloalkane_dehalogenase	1bn6	3.40.50.1820
Haemoglobin	1hda	1.10.490.10
HSA	1n5u	1.10.246.10
IgG	1igt	2.60.40.10
Insulin	1trz	1.10.100.10
Jacalin	1ku8	2.100.10.30
Lactoferrin	1blf	3.40.190.10
Lectin_(lentil)	1les	2.60.120.200
Leptin	1ax8	1.20.1250.10
Lysozyme	193l	1.10.530.10
Monellin	1mol	3.10.450.10
Myoglobin	1ymb	1.10.490.10
Myoglobin_1	1a6m	1.10.490.10
Nitrogen_metabolite_repression_r	1k6j	3.40.50.720<br>3.90.25.10
Ovalbumin	1ova	2.30.39.10<br>3.30.497.10
Ovotransferrin	1dot	3.40.190.10
Papain	1ppn	3.90.70.10
Lectin_(pea)	1ofs	2.60.120.200
Pectate_lyase_C	1air	2.160.20.10
Pepsinogen	2psg	2.40.70.10
Peroxidase_C1	7atj	1.10.520.10<br>1.10.420.10
Phosphoglucomutase_1	3pmg	3.40.120.10
Phosphoglycerate_kinase	3pgk	3.40.50.1260<br>3.40.50.1270
Phospholipase_A2	1une	1.20.90.10
Phenylethanolamine_N-methyltra	1hnn	3.40.50.150
Pyruvate_kinase	1a49	3.40.1380.20<br>3.20.20.60<br>2.40.33.10
Rhodanese	1rhs	3.40.250.10
Ribonuclease_A	3rn3	3.10.130.10
Rubredoxin	1r0i	2.20.28.10
Trypsin_inhibitor_A	1ba7	2.80.10.50
Streptavidin	1stp	2.40.128.30
Subtilisin_A	1scd	3.40.50.200
Superoxide_dismutase_[Cu-Zn]	1cbj	2.60.40.200
Thaumatin_I	1thw	2.60.110.10

Triose_phosphate_isomerase	7tim	3.20.20.70
Ubiquitin	1ubi	3.10.20.90
