# Supplementary figures and images for: PDB2CD: a web-based application for the generation of circular dichroism spectra from protein atomic coordinates
Source: Bioinformatics. 2016 Sep 20;33(1):56–63. doi: 10.1093/bioinformatics/btw554 (PMC5408769; doi:10.1093/bioinformatics/btw554)

## AVIDIN

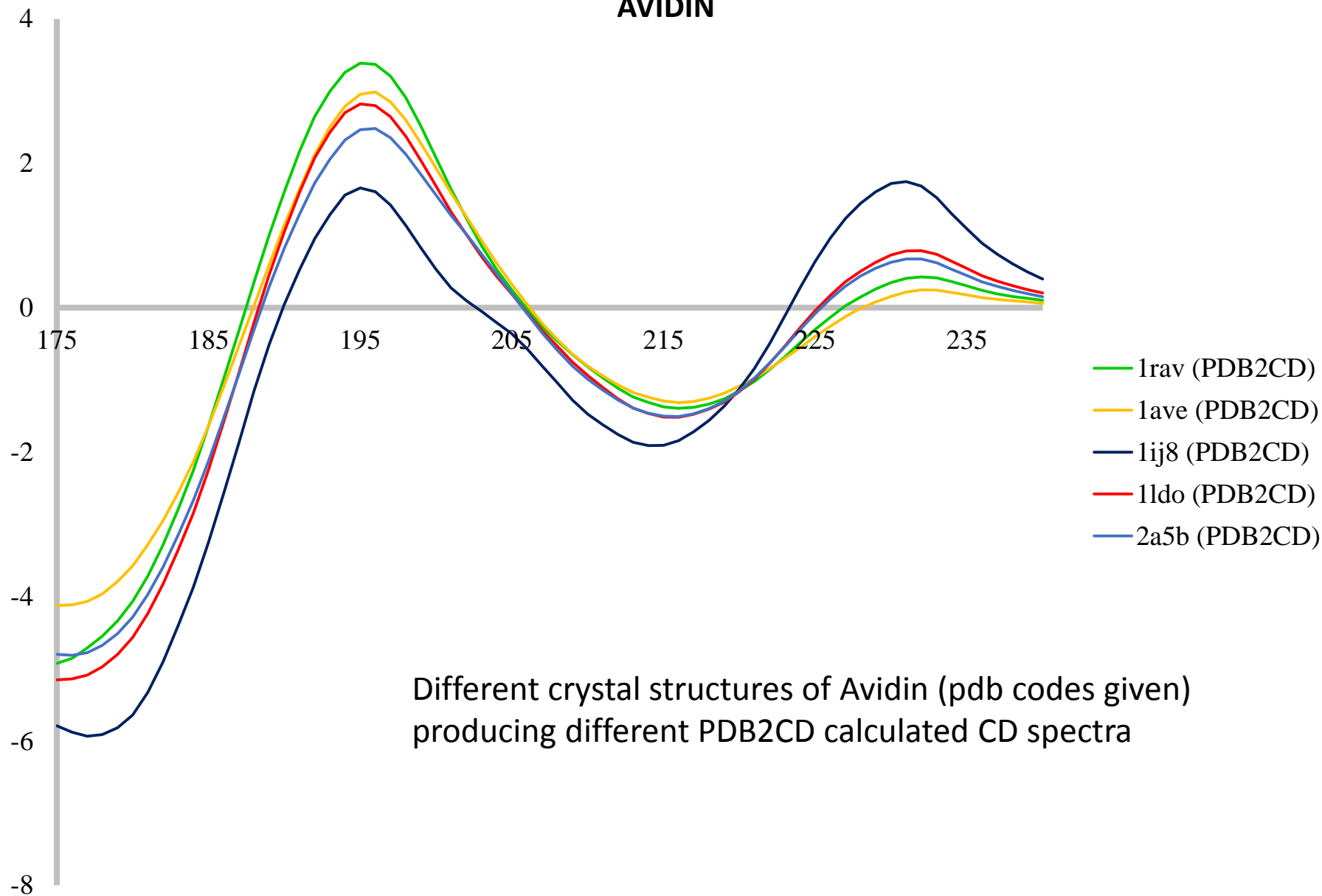

Supplement: Supplementary Data [file btw554_supp.zip › Avidin.pdf]
